# Book of Posters

**DOI:** 10.1080/24740527.2026.2685939

**Published:** 2026-07-16

## Poster Judgingévaluation de l’afficheRethinking How We Measure Pain: Global Evidence on the Influence of Wording and Design in Pain Surveys

Lindsay Neuert^1^, Matthew Fillingim^1^, Christophe Tanguay-Sabourin^1,2^, Azin Zare^1^, Jax Norman^1^, Gianluca Guglietti^1^, Lise Hobeika^1^, Etienne Vachon-Presseau^1^

^1^McGill University, ^2^University of Montreal

**Introduction**: Chronic pain affects approximately one in five people globally and remains a major source of disability, diminished quality of life, and economic strain. Although its worldwide prevalence is well recognized, efforts to measure pain consistently have been limited by substantial methodological differences across studies. As pain is a subjective experience shaped by biological, psychological, and social influences, small variations in how questions are phrased or administered can shift self-reported outcomes. Yet little is known about how these design features influence population-level pain estimates.

**Methods**: This project examines how variations in survey wording and study design shape self-reported pain prevalence, site-specific pain, and pain intensity across more than five million participants from over 400 cohorts in 40 countries. We evaluate how contextual features, such as mode of administration (oral vs. written), recruitment methods, recall periods, emotional tone of wording (e.g., “suffer/bother from pain” vs. “have/experience pain”), and intensity scale anchoring affect reported pain. Using mixed-effects models, we quantify the extent to which methodological choices contribute to between-study variability, offering the first global assessment of how question formulation and study context influence pain reporting.

**Results**: Written surveys are associated with higher pain prevalence (OR: 1.69, p < 0.05), while oral surveys show lower reporting (OR: 0.59, p < 0.05). Emotionally charged wording yields lower reporting (OR: 0.80, p < 0.05), while neutral phrasing yields higher reporting (OR: 1.25, p < 0.05).

**Discussion/Conclusions**: These insights support the development of standardized pain measurement strategies, enhancing the validity, comparability, and equity of global pain research and surveillance.

## Development of Cost-Effective 3D-Printed Lumbar and Cervical Spine Phantoms for Interventional Pain Training Using Ballistic Gel and Silicone

Jaume Nolla Suárez^1^, Diphile Iradukunda^1^

^1^Alan Edwards Pain Management Unit, McGill University Health Centre, Montreal, QC, Canada

**Introduction**: Simulation-based training allows pain fellows and residents to acquire interventional skills safely without exposing patients to risk. However, commercial phantoms for fluoroscopy- or ultrasound-guided procedures are prohibitively expensive. This project aimed to develop low-cost, durable, and anatomically realistic 3D-printed spinal models—lumbar and cervical—embedded in ballistic gel or silicone to simulate tissue resistance, radiopacity, and needle guidance for repeated procedural training.

**Methods**: Two 3D-printed spine phantoms were fabricated. The lumbar model, printed in nylon, showed no degradation when submerged in 240 °F ballistic gel and was embedded in a 10% ballistic gel mold. It tolerated more than 75 punctures per region (18-25 G needles) before requiring remelting. The cervical model, printed in nylon and embedded in Ecoflex 00-20 silicone, provided high anatomical fidelity for both ultrasound- and fluoroscopy-guided practice.

**Results**: Each phantom demonstrated excellent durability and radiopacity. The transparent ballistic gel allowed clear fluoroscopic visualization of the needle trajectory and, when handled directly, provided real-time visual feedback of needle depth and angulation, facilitating procedural reinforcement and self-correction. The models offered clear fluoroscopic contrast, realistic needle-navigation sensation, and stable structural integrity after multiple punctures. The total material and printing costs, including gel, silicone, and casting tools—were under $800 CAD for both models combined.

**Discussion/Conclusions**: Ballistic-gel and silicone-based 3D-printed phantoms offer an inexpensive, reusable, and anatomically faithful solution for interventional pain training. Their adoption could enhance procedural competence, reduce the learning-curve risk, and make high-quality simulation accessible to resource-limited academic programs.

## The intersection of chronic pain and transition experiences in shaping Veterans’ post-service needs: A mixed methods study

Erin Collins^1,2^, Jenny JW Liu^1,2^, Dominic Gargala^2^, J. Don Richardson^1,2,3,4^

^1^Western University, ^2^MacDonald Franklin OSI Research and Innovation Centre, ^3^St. Joseph’s Health Care London, ^4^McMaster University

**Introduction**: Veterans experience disproportionately high rates of chronic pain, often co-occurring with PTSD and other complex conditions. Chronic pain affects physical, psychological, and social domains of life, intensifying the challenges of military-to-civilian transition. This study examines how pain influences post-service well-being and identifies persistent gaps in critical services and support.

**Methods**: Quantitative data were drawn from a 2023 survey of 233 Canadian Veterans assessing demographics, health, and quality of life. Transition experiences were measured using the Transitioning to Civilian Life Scale (α = .915). Linear regressions adjusted for age, sex, duty status, and service years. Qualitative data were collected through interviews with 10 Veterans, 10 family members, and 11 service providers, and analyzed using a deductive-inductive approach guided by the Veteran Well-being Surveillance Framework.

**Results**: Two-thirds of participants reported moderate to severe pain-related impairments; 55.5% of these Veterans were medically released, compared to 19.4% of those without impairments. Veterans with pain reported lower life satisfaction (p < .05) and greater transition difficulty (M = 48.04 vs. 36.48; t(231) = -6.90, p < .001). After adjusting for covariates, pain remained a strong predictor of transition difficulty (B = 11.25, p < .001). Interviews revealed that chronic pain disrupted Veterans’ sense of identity, daily functioning, relationships, and mental health, compounded by fragmented care, stigma, and inadequate preparation.

**Discussion/Conclusions**: Chronic pain compounds transition challenges, predicting poorer adjustment and lower well-being. Findings highlight the need for early identification, cross-sector coordination, caregiver education, peer support, and trauma-informed pain care to strengthen continuity and civilian reintegration.

## Rurality and Remoteness: Distinct Concepts for Understanding Disparities in Chronic Pain Treatment

Assad Nassoma^1^, Claudie Audet^1^, Anaïs Lacasse^1^

^1^Département des sciences de la santé, Université du Québec en Abitibi-Témiscamingue (UQAT)

**Introduction**: Rurality (low population density) and remoteness (distance from metropolitan centres) are often used interchangeably. However, a person may live in a rural area near a metropolitan centre, or in an urban area in remote regions. This study analyzed how rurality and remoteness intersect in their association with prescribed pain medication use, outcome selected as a proof-of-concept.

**Methods**: We analyzed data from the COPE cohort, an online survey of individuals living with chronic pain in Quebec (n=1,935), of whom a subset had their questionnaire data successfully linked to administrative health databases (n=895). Rurality was defined using postal codes (a “0” in the second position) and remoteness was based on self-reported residence in one of six government-defined remote regions.

**Results**: Among 873 participants with geographic data, 11.2% lived in ‘Rural-Not Remote’, 65.6% in ‘Urban-Not Remote’, 6.4% in ‘Rural-Remote’, and 16.7% in ‘Urban-Remote’ areas. The proportion of prescribed pain medication users in ‘Rural-Not Remote’ (90.7%) and ‘Urban-Not Remote’ (86.5%) areas did not differ significantly, but both were higher than in ‘Rural-Remote’ (80.4%) areas, all three were higher than in ‘Urban-Remote’ (72.2%) areas (p < .001). Multivariable regression showed that remoteness was associated with prescribed pain medication use, whereas rurality was not, and their statistical interaction was not significant, indicating no effect modification.

**Discussion/Conclusions**: Prescribed pain medication use varied across geographic groups, highlighting the importance of measuring rurality and remoteness distinctly. The simple rural/urban distinction adds no information once remoteness is accounted for, and rurality does not modify the relationship between remoteness and use.

## Comparing statistical methods for examining time-dependent variability in chronic pain in the CircaPain study

Hailey GM Gowdy^1^, Doriana Taccardi^1^, Amanda M Zacharias^1^, Élisabeth Lamoureux^2^, Jennifer Daly-Cyr^3^, Jennifer Lorca^3^, Lesley Norris Singer^3^, Manon Choinière^4,5^, Qingling Duan^1^, Zihang Lu^6^, M Gabrielle Pagé^2,4,5^, Nader Ghasemlou^1,7,8^

^1^Department of Biomedical and Molecular Sciences, Queen’s University, Kingston, Ontario, Canada, ^2^Department of Psychology, Université de Montréal, Montreal, Quebec, Canada, ^3^Chronic Pain Network, McMaster University, Hamilton, Ontario, Canada, ^4^Department of Anesthesiology and Pain Medicine, Université de Montréal, Montreal, Quebec, Canada, ^5^Centre hospitalier de l’Université de Montréal (CHUM) Research Center, Montreal, Quebec, Canada, ^6^Department of Public Health Sciences, Queen’s University, Kingston, Ontario, Canada, ^7^Department of Anesthesiology and Perioperative Medicine, Queen’s University, Kingston, Ontario, Canada, ^8^Centre for Neuroscience Studies, Queen’s University, Kingston, Ontario, Canada

**Introduction**: It is crucial to know *why* and *when* someone experiences pain to develop individualized management strategies for chronic pain. Rhythmic low back pain fluctuations that increase in intensity throughout the day have been found to be associated with reduced opioid use, better biopsychosocial profiles, and differentially expressed transcripts. Our study explores whether distinct temporal patterns or rhythmic trends exist within self-reported pain intensity in other chronic pain conditions.

**Methods**: Following an initial questionnaire, recruited participants (N = 907) completed electronic symptom-tracking diaries, where they rated their pain intensity, affect, and fatigue using 0-10 scales daily at 3 timepoints (08:00, 14:00, 20:00) for 7 days. We applied different statistical models to this longitudinal dataset (e.g., functional principal component analysis, latent class mixed effect model).

**Results**: Compliant diaries of 1 or more full days (diary entry submitted within 2 hours of being sent) were used for the analyses (n = 585). Statistical methods identified between 2 (with latent class mixed effect modeling) and 12 clusters (with group-based trajectory modeling) based on pain intensity and patterns of variation. Rhythmic pain fluctuations emerged as a common cluster between each statistical method and were associated with more favourable biopsychosocial profiles (e.g., less severe depressive symptoms and fatigue, p < 0.01 or less).

**Discussion/Conclusions**: The inter-individual differences in pain variability observed in our sample present a tool to characterize chronic pain. The biopsychosocial profiles associated with rhythmic pain suggest it may be an important phenotype in the clinical setting.

This work is supported by CIHR and the CIHR-SPOR Chronic Pain Network.

## Defining Usual Care: Variability in Knee Osteoarthritis Trial Comparators

Victoria D’Alessandro^1^, Dimitra Pouliopoulou^1^, Marjan Saeedi^1^, Joy MacDermid^1^, Nicole Billias^1^, Aidan Loh^1^, Jessica Wong^1^, Trevor Birmingham^1^, Lauren King^2^, Tiago Pereira^3^, Bruno da Costa^3^, Pavlos Bobos^1^

^1^University of Western Ontario, ^2^University of Toronto, ^3^Oxford University

**Introduction:** “Usual care” is a common comparator in randomized trials (RCTs) evaluating interventions for knee osteoarthritis (OA), yet it lacks a standardized definition. This review mapped how usual care is defined and reported in knee OA RCTs, focusing on terminology, components, and adherence to reporting standards.

**Methods**: We searched MEDLINE, EMBASE, CENTRAL, CINAHL, and ClinicalTrials.gov for RCTs involving adults with knee OA that compared a non-surgical intervention to usual care or similar terms. Paired reviewers independently screened citations and extracted data on study characteristics, terminology used, components of usual care, care setting, intervention type, and references to external guidelines. Reporting quality was assessed using the Template for Intervention Description and Replication (TIDieR) and Consensus on Exercise Reporting Template (CERT).

**Results**: Of 11,804 citations screened, 154 RCTs were included. “Usual care” was the most common descriptor (53.9%), but definitions varied widely. While 68.2% of trials provided detailed descriptions, nearly one-third reported usual care only vaguely, and just 22.7% referenced external guidelines. Usual care ranged from minimal advice to multimodal programs, with substantial variation by intervention category. Reporting was suboptimal: trials reported just over half of TIDieR items and roughly two thirds of CERT items. Overall, usual care lacked transparency, standardization, and alignment with best practice recommendations.

**Discussion/Conclusions**: One in three knee OA trials did not clearly define their usual care comparator. Inconsistent definitions and poor reporting reduce trial interpretability, limit evidence synthesis, and hinder clinical translation. Improved standardization of detailed reporting of usual care in knee OA trials is urgently needed.

## Pain Management in Heritable Connective Tissue Disorders: Insights from a Systematic Review of Clinical Guidelines in Ehlers-Danlos, Loeys-Dietz, and Marfan syndromes

Catherine Isadora Côté^1,2^, Élisabeth Lamoureux^1,2^, Karen Ghoussoub^1,2^, Tania Augière^2^, Vera Granikov^2^, Annie-Danielle Grenier^3,4^, Mizaël Bilodeau^1^, Hance Clarke^5,6^, Audrey L’Espérance^7^, Anne-Marie Laberge^1,8^, Anaïs Lacasse^9^, Camille Laflamme^1,10^, Marie-Pascale Pomey^1,2^, Élise Develey^2^, M Gabrielle Pagé^1,2^

^1^Université de Montréal, ^2^Centre de recherche du centre hospitalier de l’Université de Montréal (CRCHUM), ^3^Centre d’excellence sur le partenariat avec les patients et le public, ^4^Institut de recherches cliniques de Montréal (IRCM), ^5^University of Toronto, ^6^GoodHope Ehlers Danlos Syndrome Clinic, ^7^École nationale d’administration publique (ENAP), ^8^Centre hospitalier universitaire Sainte-Justine (CHUSJ), ^9^Université du Québec en Abitibi-Témiscamingue, ^10^Centre hospitalier de l’Université de Montréal

**Introduction**: Pain is common and disabling in heritable connective tissue disorders (HCTDs), affecting up to 90% of people with Ehlers-Danlos (EDS), Loeys-Dietz (LDS), or Marfan (MFS) syndromes. It often begins early and takes many forms, such as musculoskeletal, neuropathic, visceral, or pelvic pain, and is frequently coexisting with fatigue and psychological distress. Yet, clinical guidance on pain assessment and management across HCTDs remains sparse and inconsistent.

**Aim**: To identify and compare pain assessment and management recommendations in clinical practice guidelines and consensus statements for MFS, LDS, and EDS.

**Methods**: This analysis stems from a broader systematic review of all recommendations on these syndromes (PROSPERO: CRD42024590069). Following PRISMA standards, major databases (MEDLINE, Embase, PsycINFO, EBM Reviews, CINAHL) and grey literature were systematically searched. All stages—screening, full-text review, data extraction, and quality appraisal—were independently completed by two of five reviewers using AGREE-II for methodological quality and GRIPP-2 for patient involvement. Conflicts were solved by discussion between raters or a third reviewer.

**Results**: Among 4,893 records screened, 85 guidelines were included; 20 contained pain-related recommendations. Most focused on hypermobile EDS (hEDS), while guidance for MFS, LDS, and other EDS subtypes was minimal or absent. Existing recommendations (n=5) for vascular EDS, LDS, and MFS only addressed chest pain linked to aortic dissection, while hEDS guidance emphasized multidisciplinary care, graded exercise, and joint protection.

**Conclusion/Discussions**: Pain guidance in HCTDs is largely limited to hEDS. Expanding evidence-based, multidisciplinary pain guidelines to all HCTDs—while integrating cardiovascular considerations—is a key unmet need.

## Quality Indicators for Chronic Pain Management: An Umbrella Review

Claudie Audet^1^, M. Gabrielle Pagé^2^, Nancy Julien^1^, Marimée Godbout-Parent^1^, Andréanne Bernier^1^, Florian Alatorre^3^, Catherine Héroux^1^, Hermine Lore Nguena Nguefack^1^, Isabelle Dufour^4^, Line Guénette^5^, Lucie Blais^6^, Manon Choinière^2^, Mark A. Ware^7^, Matthew Menear^8^, Regina Visca^9^, Yannick Tousignant-Laflamme^10^, Yohann Moanahere Chiu^11^, Anaïs Lacasse^1^

^1^Département des sciences de la santé, Université du Québec en Abitibi-Témiscamingue, ^2^Research Center of the Centre hospitalier de l’Université de Montréal (CRCHUM); Department of anesthesiology and pain medicine, Faculty of Medicine, Université de Montréal, ^3^Centre hospitalier de l’Université de Montréal (CRCHUM), ^4^École des sciences infirmières, Faculté de médecine et des sciences de la santé, Université de Sherbrooke, ^5^Faculté de pharmacie, Université Laval; Axe Santé des populations et pratiques optimales en santé, Centre de recherche du CHU de Québec - Université Laval, ^6^Faculté de pharmacie, Université de Montréal, ^7^Department of Family Medicine, Mc Gill University, ^8^Département de médecine familiale et médecine d’urgence de la Faculté de médecine de l’Université Laval; VITAM - Centre de recherche en santé durable, ^9^Mc Gill’s Centre of Expertise in Chronic Pain, ^10^École de réadaptation, Faculté de médecine et des sciences de la santé, Université de Sherbrooke, ^11^Département de médecine de famille, Faculté de médecine et des sciences de la santé, Université de Sherbrooke

**Introduction**: Measuring quality indicators helps evaluate and improve care, inform policy, and support effective, patient-centred management of chronic pain. Although numerous reviews have proposed quality indicators for chronic pain management, the evidence remains fragmented, and their practical application is not yet well defined. This umbrella review (overview of reviews) synthesized existing evidence on quality indicators for chronic pain management.

**Methods**: Systematic and non-systematic reviews listing quality indicators relevant to the management of chronic pain and published in English or French were included. Articles were retrieved in July 2025 through searches of MEDLINE, CINAHL, PsycINFO, EMBASE, and EBM Reviews All. Two independent reviewers completed article selection. Results were summarized narratively (tables and figures). The quality of reviews included was assessed using the *JBI Critical Appraisal Checklist for Systematic Reviews and Research Syntheses*. The review protocol was registered with INPLASY® (202570050).

**Results**: Of the 3503 records identified, 148 were reviewed in full-text, and 53 were included. Among the indicators identified, several domains emerged (e.g., patient education, pain and functional assessment, pharmacological and non-pharmacological treatments, referrals to other professionals or to surgery, follow-up, multidisciplinary services, patient satisfaction, staff education). These indicators were measurable across various data sources, including health administrative data, electronic health records, patient registries, and patient questionnaires or interviews.

**Discussion/Conclusions**: This literature synthesis is an important first step in identifying quality indicators for chronic pain management. The next step is to validate these indicators for use by decision-makers, health system managers, and knowledge users.

## Increased synaptic drive onto polymodal Tac1-lineage dorsal horn neurons after nerve injury

Pauline Larqué^1^, Louison Brochoire^1^, Yves de Koninck^1,2^, Feng Wang^1,3^

^1^CERVO research center, Université Laval, ^2^Faculté de Médecine, Université Laval, ^3^Faculté de Médecine Dentaire, Université Laval

**Introduction**: The spinal cord plays a crucial role in relaying sensory information. Yet, the precise neuronal circuits mediating this processing remain incompletely understood, due to the scarcity of specific genetic markers. Studies have highlighted neurons expressing the Tachykinin 1 (Tac1) gene to be essential for coping behaviors in response to intense noxious stimuli. In this study, we will study their contribution to somatosensory processing.

**Methods**: We used immunochemistry to study the distribution of Tac1-lineage neurons across the dorsal horn of the spinal cord. Then we used a combination of whole-cell patch-clamp recordings from parasagittal spinal cord slices and *in vivo* two-photon calcium imaging to respectively study physiology and function of these neurons. These experiments were performed in both naïve and neuropathic pain conditions (spared nerve injury).

**Results**: Immunohistochemistry using Tac1-tdTomato mice revealed that Tac1-lineage neurons are scattered across multiple lamina in the dorsal horn. These neurons display diverse intrinsic firing properties. However, the majority exhibited single-spike or phasic firing patterns, suggesting a potential role as coincidence detectors in information processing. The majority of superficial Tac1-lineage neurons are polymodal nociceptive, responding to both noxious mechanical and thermal stimuli. In neuropathic pain mice, preliminary data showed that the frequency of spontaneous excitatory postsynaptic current was increased in Tac1-lineage neurons compared to naïve condition. Intriguingly, we did not observe significant changes in their intrinsic firing properties or responsiveness to peripheral mechanical and thermal stimuli, suggesting that nerve injury alters surrounding spinal circuitry rather than directly affecting Tac1-lineage neurons.

**Discussion/Conclusions**: Together, our findings demonstrated that superficial Tac1-lineage neurons are primarily polymodal nociceptive and may integrate elevated synaptic drive from local spinal circuits after nerve-injury, potentially contributing to the neuropathic pain.

## Endogenous Modulation Reduces Pain Without Reducing Its Informational Value

Antoine Cyr Bouchard^1^, Nicolas Roy^1^, Laurence Lagadec-Gaulin^1^, Mégane Déry^1^, Jacob Schink^1^, Michel-Pierre Coll^1^

^1^Université Laval

**Introduction: Pain** provides essential informational value that supports learning and adaptive behaviour. Theories of pain and learning propose that endogenous modulation, such as placebo analgesia, reduces pain without impairing the perceptual processes necessary for learning. However, whether placebo analgesia alters fine-grained discrimination remains unclear. This study tested whether endogenous modulation affects the ability to detect subtle increases in thermal pain.

**Methods**: 59 participants completed an individually calibrated thermal discrimination task. Placebo analgesia was induced through combined verbal suggestion and conditioning using a sham TENS device. Participants received painful thermal stimulations at a constant, individually determined intensity, with or without a brief temperature pulse. They indicated whether they perceived the pulse. Pain ratings and discrimination accuracy were compared between placebo (TENS “active”) and control (TENS “inactive”) conditions.

**Results: Most** participants showed a placebo effect, reporting significantly lower pain during placebo trials. Despite this endogenous modulation, discrimination accuracy did not significantly differ between placebo and control conditions (73.45% vs. 77.40%). Bayesian analyses and equivalence testing provided no evidence for a meaningful impairment in discrimination performance under placebo analgesia, although very small effects cannot be fully excluded.

**Discussion/Conclusions**: Placebo analgesia reduced perceived pain without compromising fine-grained discrimination of nociceptive intensity. These findings support theoretical accounts proposing that endogenous modulation preserves the informational value of pain in order to maintain effective learning and adaptive behaviour. Further work should examine whether similar results generalize across body sites and other forms of endogenous modulation.

## Sex differences in the diurnal variability of pain sensitivity in healthy individuals

Doriana Taccardi^1^, Hailey GM Gowdy^1^, Amanda M Zacharias^1^, Zihang Lu^2^, Tim V Salomons^3^, Nader Ghasemlou^1,4,5^

^1^Department of Biomedical and Molecular Sciences, Queen’s University, Ontario, Canada, ^2^Department of Public Health Sciences, Queen’s University, Ontario, Canada, ^3^Department of Psychology, Queen’s University, Ontario, Canada, ^4^Department of Anesthesiology & Perioperative Medicine, Queen’s University, Ontario, Canada, ^5^Centre for Neuroscience Studies, Queen’s University, Ontario, Canada.

**Introduction**: The sensation of pain is a universal human experience, with variable and dynamic intensity within the day and between individuals. Recent work using a controlled environment found circadian (24-hour) fluctuations in pain sensitivity in a small cohort of healthy males, independent of sleep. We sought to determine whether sex differences exist in pain sensitivity assessed within a day in healthy individuals.

**Methods**: Adults (N=101, 50 males, 51 females) were recruited to investigate differences in sensitivity to thermal heat stimulation using Quantitative Sensory Testing (QST). Participants completed two QST sessions within 24 hours: one session in the morning (10:00-12:00) and another in the evening (22:00-00:00), randomized to time of first stimulation. Each session included assessment of thermal sensitivity (heat pain threshold; HPT, averaged across four stimuli) and pain intensity (0-10 pain rating after stimulation corresponding to threshold +2 °C). A mixed-effect model considering HPT, sex, time of day, order of time point, and interaction between sex and time of day was used.

**Results**: Fixed effects were significant for HPT and female sex (p<0.001). Individuals with higher HPTs experienced a more painful pain stimulus, independent of sex or time of day. Furthermore, females consistently reported greater pain intensity than males, even when accounting for differences in HPT and time of day.

**Discussion/Conclusions**: Our study suggests that both thermal sensitivity and biological sex influence pain perception. While we did not observe time effects, additional work is needed to understand whether considering temporal components could be important to explain individual differences in pain experiences.

## Seeking and accessing care for chronic pain related brain fog: A constructivist grounded theory study

Ronessa Dass^1^, Sandra Vanderkaay^1^, Lyn Turkstra^1^, Kalee Dass^2^, Susan Clarke-Tizzard^2^, Max Farrell^2^, Ed Cruz^2^, Steve Button^2^, Tara Packham^1^

^1^McMaster University, ^2^None

**Introduction**: Veterans with chronic pain may experience brain fog, a phenomenon which may influence one’s self-perception, engagement in social roles, and ability to manage their health. Few studies have explored these impacts within the context of accessing and seeking healthcare services. The objective of this study was to develop a conceptual model of brain fog in Veterans with chronic pain to guide self-management and treatment planning for brain fog. The research questions were: 1) how do Veterans with chronic pain experience brain fog? And 2) how do Veterans with chronic pain and brain fog access and seek health care services to manage brain fog?

**Methods**: This study used a constructivist grounded theory approach. Data was generated using semi-structured interviews and analysis was facilitated using Quirkos^TM^ software.

**Results**: A total of 21 participants (15 Veterans, 6 care providers) were included in this study. This study integrated lived experiences and provider perspectives to describe a conceptual model of the core features of the experiences of accessing and seeking management strategies for brain fog, as well as the complex relationships embedded within these features.

**Discussion/Conclusions**: The constructed model represents how Veterans with chronic pain-related brain fog make sense of their experiences, describe brain fog to others and seek management strategies. Researchers and healthcare professionals can utilize these findings to identify potential mechanisms of brain fog and strategies for treatment, prioritizing strength-based solutions incorporating individual values and identity.

## Illustrating Chronic Pain: Art Workshops Exploring Its Meanings Among Indigenous People

Joséanne Desrosiers^1^, Hugo Asselin^1^, Amélie Blanchet Garneau^2^, Stéphane Laroche^3^, Elizabeth Larouche^3^, Nancy Julien^1^

^1^UQAT, ^2^UdeM, ^3^CAAVD

**Introduction: Research** has long been a colonial undertaking. For many years, researchers have conducted studies *on* rather than *with* Indigenous people, positioning themselves as experts and rarely seeking Indigenous perspectives. These practices caused harm, perpetuated injustices, and fostered lasting mistrust toward research. Fortunately, research practices are evolving, with new guidelines promoting studies conducted *with* and *by* Indigenous People.

**Methods**: Guided by these principles, we co-led a decolonial, collaborative, and creative qualitative project exploring, through art workshops, the meanings of chronic pain among Indigenous people. Our team is composed of university researchers and practitioners from the Val-d’Or Indigenous Friendship Centre (Québec, Canada) collaborated throughout all stages of the project. We conducted two art workshops facilitated by a local professional Eeyou artist.

**Results**: Thirteen Anicinapek and Eeyouch participants used painting to illustrate their chronic pain. We conducted a thematic analysis including the narrative explanations provided by participants about their artworks. Through their narrative, we were able to understand how participants make sense of their chronic pain, often using analogies rooted in Indigenous cultures. Their visual representations also illustrate how chronic pain relates to time, invisibility, and burden.

**Discussion/Conclusions**: Our collaborative project highlighted the usefulness of art to express the meanings and complexity of chronic pain. Participating in art workshops helped Indigenous participants communicate more effectively. Our findings suggest that integrating art and Indigenous cultural elements can help with pain assessment, improve communication and understanding between clinicians and Indigenous patients, and contribute to culturally safe healthcare practices.

## Cannabis craving and cannabis-related problems among persons with chronic pain: Investigation with an ecological momentary assessment (EMA) study

Amanda Sirois^1,2^, Lucas Frankel^3^, Gabriella Spiegler^4^, Jiaqi Bi^5^, Daniel Rosenthal^6^, Maria Verner^2^, Abdulelah Binshihah^2^, Jonathan Hudon^2,7,8,9,10^, Maayan Ben-Sasson^2^, M. Gabrielle Pagé^2,11^, Jordi Perez^2,8^, Mark Ware^2,9^, Mary-Ann Fitzcharles^2,12^, Marc O. Martel^1,2,8^

^1^Faculty of Dental Medicine and Oral Health Sciences, McGill University, ^2^Alan Edwards Pain Management Unit, McGill University Health Centre, ^3^Department of Psychology, McGill University, ^4^Department of Epidemiology, Biostatistics, and Occupational Health, McGill University, ^5^Department of Epidemiology and Biostatistics, Schulich School of Medicine & Dentistry, Western University, ^6^Department of Psychology, Concordia University, ^7^Division of Supportive and Palliative Care, Jewish General Hospital, ^8^Department of Anesthesia, McGill University, ^9^Department of Family Medicine, McGill University, ^10^Edwards Family Interdisciplinary Centre for Complex Pain, Montréal Children’s Hospital, ^11^Department of Anesthesiology and Pain Medicine, Université de Montréal, ^12^Division of Rheumatology, McGill University

**Introduction**: Patients with chronic pain have increasingly turned to cannabis for the management of their symptoms. To date, little is known on the day-to-day factors contributing to patients’ desires (i.e., cravings) to use cannabis and cannabis-related problems in this population.

**Objectives**: To examine the contribution of pain intensity, psychological states, and cannabis subjective effects to cannabis craving and cannabis intake. We also examined the influence of cannabis use patterns and cannabinoid types (i.e., THC, CBD) on cannabis-related problems.

**Methods**: In this ecological momentary assessment (EMA) study, patients (n = 103) with chronic pain completed electronic diaries multiple times per day over 10 consecutive days. Diaries assessed a host of pain, psychological, and cannabis-related variables.

**Results**: Multilevel analyses indicated that intra-day elevations in pain intensity, nervousness, and depressed mood were associated with heightened cannabis craving (all p’s < .001). Intra-day elevations in cannabis craving were also associated with a greater likelihood of momentary cannabis use (p < .01). In addition, higher momentary cannabis craving was associated with greater standardized THC intake (p < .05). Poisson regression analyses indicated that higher pain intensity ratings were associated with greater frequency of cannabis use across the 10-day period (p < .01). Furthermore, multilevel analyses indicated that higher daily frequency of cannabis use was associated with greater cannabis-related problems across the 10 diary days (p < .001).

**Discussion/Conclusions**: Our findings provide new insights into the factors contributing to cannabis craving and day-to-day cannabis use problems among patients with chronic pain.

## Solace Safety Framework for Responsible Use of Generative AI when Providing Mental Health Support for Chronic Pain

Binh Nguyen^1^, Tahir Janmohamed1^1^, Hance Clarke^1,2,3,4^, Joel Katz^1,2,3,4,5^, P. Maxwell Slepian^1,2,3,4^

^1^ManagingLife, Inc., Toronto, ON, ^2^Department of Anesthesia and Pain Management, Toronto General Hospital, University Health Network, Toronto, ON, ^3^Department of Anesthesiology and Pain Medicine, University of Toronto, Toronto, ON, ^4^University of Toronto Centre for the Study of Pain, University of Toronto, Toronto, ON, ^5^Department of Psychology, York University

**Introduction**: When generative artificial intelligence (Gen-AI) is used to provide psychological support, ensuring user safety is paramount. Otherwise, such systems risk offering inaccurate/misleading guidance, mishandling high-risk situations (e.g., suicidal ideation), or being mistaken as a substitute for professional care. These risks can compromise well-being, erode trust, and discourage seeking professional help.

**Objectives**: We developed a safety framework for the responsible use of Gen-AI to guide the design, testing, and deployment of Solace, a first-of-its-kind, expert-trained AI companion that delivers real-time, evidence-based pain psychology support. The framework aims to minimize potential harm, build trust, and establish a replicable model for safe and ethical Gen-AI engagement in mental health contexts.

**Methods**: The framework integrates five pillars: guardrails and fail-safes to detect high-risk situations and enable crisis off-ramps; validation through automated regression testing, simulated conversations, and expert review to identify bias, stigma, and safety issues; ecosystem monitoring to track academic, regulatory, and industry developments; transparency and trust through disclaimers and clear communication of limitations; and governance by an AI Ethics Review Committee of clinicians, researchers, and individuals with lived experience.

**Result**: Solace successfully passed 44 automated tests designed by clinical and technical experts to assess high-risk scenarios, 11 additional tests identified through ecosystem monitoring, 16 bias and stigma simulation tests, where AI-generated personas were used to elicit biased/stigmatizing responses. All outputs were reviewed by clinical experts to confirm acceptable responses.

**Discussion/Conclusions**: The safety framework provides a replicable model for safe and responsible Gen-AI use in supporting mental health, ensuring user safety and well-being.

## No effect of rhythmic visual stimulation on experimental pain perception

Nicolas Roy^1,2^, Thaliane Côté-Cazes^1^, Coralie Deslauriers^1^, Audrey Etcheverry^1^, Michel-Pierre Coll^1,2^

^1^Université Laval, ^2^CIRRIS

**Introduction**: Brain oscillations, particularly in the alpha, beta and gamma bands have been implicated in pain perception through correlational studies. Rhythmic visual stimulation (RVS) offers a non-invasive method to manipulate these oscillations and investigate their causal role in pain perception. We conducted two experiments to assess whether RVS modulates acute pain perception and pain-related neural oscillations. ‘

**Methods**: In Experiment 1, 41 participants either received brief (13s) RVS at alpha (10 Hz), beta (18 Hz), or gamma (42 Hz) frequencies, or an arrhythmic control stimulation, while experiencing calibrated thermal pain with concurrent electroencephalography (EEG) recordings. In Experiment 2, 49 participants underwent 10-minute RVS at either 10 Hz or at a 1 Hz control before rating electrical or laser pain stimuli, attempting to replicate previous positive findings.

**Results**: In Experiment 1, despite successfully increased neural oscillations at targeted frequencies confirmed by EEG, pain-evoked oscillatory responses remained mainly unchanged across RVS conditions in alpha, beta, and gamma bands. Across the two experiments using different stimulation protocols and pain modalities, we found no evidence that RVS modulates acute pain perception or pain-related neural oscillations, with Bayesian analyses providing strong evidence for the null hypothesis.

**Discussion/Conclusions**: These null findings contrast with some previous reports but align with emerging mixed evidence in the literature, suggesting that pain-modulatory effects of rhythmic sensory stimulation may be less robust or more context-dependent than initially proposed.

## Animal or Non-Human pain

### La douleur animale ou non humaine

## Orexin antagonism attenuates pain-related and withdrawal-related negative affect without altering opioid analgesic efficacy

Alicia Zumbusch^1^, Kimberly Newman^1^, Kelly Huang^1^, Gary Aston-Jones^1^

^1^Rutgers Brain Health Institute

**Introduction**: Two-thirds of those who misuse opioids do so for pain management, and up to 12% go on to develop an opioid use disorder. The negative reinforcing qualities of opioid withdrawal are one of the ways that opioid addiction is perpetuated. The neuropeptide orexin (OX) influences opioid withdrawal severity and pain processing, though the way that orexin influences pain and opioid withdrawal-related affect is unclear. This study aims to determine if administration of the orexin antagonist suvorexant during opioid withdrawal impacts mechanical hypersensitivity, as well as pain- and withdrawal-related negative affect, and opioid analgesia. We hypothesize that suvorexant will attenuate negative affect associated with both pain and withdrawal and will not alter nociception or disrupt the analgesic efficacy of opioids.

**Methods**: We assigned male and female Long-Evans rats to either the vehicle or suvorexant group, then tested them for mechanical hypersensitivity (von Frey), pain-related negative affect (Rat Grimace Scale), and anhedonia (saccharin preference) before and during chronic inflammatory paw pain (Complete Freund’s Adjuvant) and opioid withdrawal from oxycodone as well as after a 3mg/kg dose of oxycodone.

**Results**: Suvorexant significantly increased paw withdrawal thresholds on the contralateral paw but did not alter nociception on the ipsilateral paw. Suvorexant also decreased the amount of pain-related facial grimacing and anhedonia without impeding opioid analgesia.

**Discussion/Conclusions**: These data indicate that when opioids are used to treat pain, suvorexant may be a useful adjunct therapy for attenuating negative affect and thus decreasing the risk of opioid use disorder.

## Monitoring Pain-Related Neural Activity Through a Minimally Invasive Blood-Based Reporter System

Sandra J. Poulson^1^, Emily Linz^1^, Shirin Nouraein^2^, Sangsin Lee^2^, Jerzy Szablowski^2^, Gregory Corder^1^

^1^University of Pennsylvania, ^2^Rice University

**Introduction**: Common approaches to determine whether specific brain regions are engaged by painful stimuli are 1) the binary counting of activated cells revealing only a snapshot in time, or 2) calcium imaging techniques that rely on an invasive implant with only small areas of examination. Here, we present the use of a novel method to measure neural activity in vivo through a simple blood test, allowing for temporal monitoring of neural activity in rodents.

**Methods**: We employ the use of protein reporters called released markers of activity (RMAs), a technology developed by the Szablowski Lab at Rice University. RMAs are packaged in gene delivery vectors, can be delivered to a CNS region of choice, and consist of an easily detectable luciferase protein and receptor-binding domain allowing transcytosis of luciferase across the blood-brain barrier. After vector expression, we used a retro-orbital method to collect blood samples at different time points after pain stimulus and measured the change in light released by the reporter compared to baseline.

**Results**: We show increased fold change in RMAs from baseline in the formalin model of inflammatory pain and the spared nerve injury model of chronic pain, as well as histological analysis of RMA expressing neurons. We also demonstrate RMA technology in genetically targeted neuron populations.

**Discussion/Conclusions**: RMA technology represents a novel, minimally invasive in vivo method that allows detection of neural activity due to painful stimuli. Increased understanding of how CNS regions shape the perception of pain will guide development of targeted therapies to alleviate pain in real-time.

## Examining the sex specific contribution of TNFR1 in nociceptors for pain in autoimmune disease

Rory H. Karbonik^1^, Andrea G. Klassen^1^, Gustavo Tenorio^1^, Jason R. Plemel^1^, John R. Bethea^2^, Bradley J. Kerr^1^

^1^University of Alberta, ^2^George Washington University (D.C.)

**Introduction**: Inflammatory mediators such as TNF can significantly impact neural plasticity and pain hypersensitivity in a sex-specific manner. Whether TNFa signalling leads to sex specific changes in autoimmune disease is not yet fully understood. This study examined the impact of deleting the TNFa receptor, TNFR1, in nociceptors on pain and neural plasticity responses in a mouse model of autoimmune disease in male and female mice.

**Methods**: A mouse *Nav1.8Cre;TNFR1 fl/fl* line was generated to selectively delete TNFR1 from nociceptors (Nav1.8-TNFR1-/-). Baseline mechanical, thermal and motor behaviours were assessed prior to experimental autoimmune encephalomyelitis (EAE) to confirm that TNFR1 loss did not alter normal sensory or motor function. Following EAE induction, these same behavioural assays were used to evaluate sex dependent changes in pain sensitivity in autoimmune related pain.

**Results**: We observed no baseline differences between control and Nav1.8-TNFR1-/- mice for thermal and mechanical sensitivity, indicating that TNFR1 deletion in nociceptors does not impact basal pain thresholds. In the EAE model, mice develop cold and mechanical hypersensitivity at the onset of clinical disease. Male Nav1.8-TNFR1-/- with EAE were however, protected from developing cold allodynia. In contrast, cold allodynia was *exacerbated* in female Nav1.8-TNFR1-/- compared to wild type controls. Spontaneous pain measured using the PainFace algorithm is currently being assessed.

**Discussion/Conclusions**: Our findings reveal a clear sex-specific role for TNFR1 in autoimmune pain. TNFR1 deletion decreases hypersensitivity in males but exacerbates pain in females. These results highlight the importance of considering sex as a biological variable in the treatment of autoimmune pain.

## Forebrain to Midbrain Cholecystokinin Inputs Mediate Nocebo Hyperalgesia from Contextual Sources in Mice

Sandra J. Poulson^1^, Aleksandrina Skvortsova^2^, Damien C. Boorman^1^, Seyed Asaad Karimi^1^, Fatama T. Zahra^1^, Lisiê Paz^3^, Wanning Cui^1^, Antonietta Mandatori^1^, Jacob Burek^1^, Anton Dinh^1^, Lianfang Liang^2^, Robert Contofalsky^2^, Jeffrey S. Mogil^2^, Loren J. Martin^1^

^1^University of Toronto, ^2^McGill University, ^3^Pontifical Catholic University of Rio Grande do Sul

**Introduction**: The nocebo effect—the phenomenon where negative expectations lead to harmful outcomes, such as increased pain sensitivity (hyperalgesia)is a significant challenge in clinical practice. In humans, nocebo hyperalgesia is known to be blocked by proglumide, a cholecystokinin (CCK) receptor antagonist. However, the specific underlying neural circuitry responsible for this effect has remained largely unknown due to a lack of robust animal models. Identifying this circuit is crucial for developing targeted pain interventions.

**Methods**: To address this gap, our two independent laboratories developed convergent mouse models of CCK-dependent nocebo hyperalgesia, generated either through environmental conditioning or social observation cues. Using these models, we employed a combination of optogenetic and pharmacological approaches, alongside behavioral assessments, to map and functionally characterize the neural pathway mediating the nocebo effect.

**Results**: We discovered that both environmentally conditioned and socially transmitted nocebo hyperalgesia share a common mechanism. Specifically, we investigated CCK-expressing neural projections from the anterior cingulate cortex (ACC) to the lateral column of the periaqueductal gray (lPAG). Both pharmacological blockade of CCK receptors in the lPAG and optogenetic inhibition of ACC CCK+ projections at the lPAG significantly attenuated nocebo hyperalgesia across both paradigms. Further, optogenetic activation of ACC-lPAG projections enhanced mechanical sensitivity.

**Discission/Conclusions**: Our findings reveal the CCK-expressing ACC-lPAG projection as a shared neural circuit for pain anticipation and nocebo hyperalgesia, regardless of whether the expectation is derived from environmental or social cues. This provides a crucial foundation for future research aimed at understanding how contextual information is integrated to enhance painful experiences.

## Assessment, Diagnosis and Measurement of Pain

### Évaluation le diagnostic et la mesure de la douleur

## Evaluating the Feasibility of AI-Driven Technology to Enhance Pain Assessment in Hospitalized Older Adults

Danielle Dunwoody^1^, Dawn Prentice^1^, Greg Thomson^2^, Callie Loveday^1^

^1^Brock University, ^2^Halton Healthcare

**Introduction**: The prevalence of dementia is rising worldwide while our approach to assessing pain within the population remains stagnant. Pain is a significant issue for people with dementia, with nurses’ approaches lacking consistency and standardization further compounding the challenges faced by this population. The objectives for this study were to examine the acceptability and feasibility of using the AI-driven application PainChek® to identify pain within older adults with cognitive impairment in the inpatient setting within Ontario.

**Methods**: A prospective cohort design was employed. Six nurses were recruited and completed standardized training. Following the recruitment and onboarding of nurse participants, 25 patient participants were enrolled and assessed using the App. Once patient data collection was complete, qualitative interviews with nursing participants were collected. Descriptive statistics were used for patient and nurse participant data, and descriptive content analysis captured nurses’ experiences with PainChek® during the study.

**Results**: Participant demographics included 16% with delirium, 20% with cognitive impairment and 64% with dementia. Average length of stay on study was 7.8 days, with an average pain score while being assessed as mild pain. Thematic coding was conducted using the nursing metaparadigm as the guiding conceptual framework.

**Discussion/Conclusions**: Our study demonstrates that it is acceptable and feasible to use PainChek® within the older adult hospital setting; however, additional research is warranted to evaluate its feasibility and scalability in real-world environments.

## Discordance between a stress biomarker and self-reported stress in a population with chronic low back pain: preliminary results

Karen Ghoussoub^1,2^, Mael Gagnon-Mailhot^3^, Élise Develay^1^, Pierre Rainville^2^, Sonia Lupien^2^, Lise Dassieu^4^, Mathieu Roy^3^, Étienne Vachon-Presseau^3^, M. Gabrielle Pagé^1,2^

^1^Research Center of the CHUM, ^2^Université de Montréal, ^3^McGill University, ^4^Université du Québec à Montréal

**Introduction**: Four characteristics that activate the hypothalamic-pituitary-adrenal (HPA) axis and lead to glucocorticoid production (cortisol in humans) have been identified: Sense of low control, social-evaluative Threat, Unpredictability, and Novelty (STUN) [1, 2]. However, glucocorticoid secretion and self-reported stress are not necessarily correlated in healthy populations [3-5]. This study aimed to examine the concordance between a stress biomarker (salivary cortisol) and self-reported stress (perceived stress and STUN characteristics) in individuals with chronic low back pain (cLBP).

**Methods**: Participants (n = 129; 51.9% women; age = 49.5 ± 13.3 years) living with cLBP completed electronic diaries three times daily and provided saliva samples five times daily for three non-consecutive days. Cortisol indicators, area under the curve with respect to ground (AUC_g_) and increase (AUC_i_), were computed. Diaries assessed pain and stress intensity and the extent to which stressors were attributable to each STUN characteristic.

**Results**: No significant associations were found between perceived stress and cortisol indicators. Similarly, STUN characteristics were not associated with the AUC_g_. Social-evaluative threat was positively associated with AUC_i_ using manually entered sampling times (β = 0.030, p < 0.05, CI 95% [0.003; 0.058]), but this effect was not replicated when analyses were based on automatic timestamps (p = 0.097).

**Discussion/Conclusions**: There is a discordance between self-reported stress and a stress biomarker in cLBP populations. Self-reported stress may reflect emotional dysregulation rather than HPA-axis activation, a distinction that may be relevant to pain mechanisms. Social-evaluative threat predicted higher AUC_i_ (manual timing), suggesting a potential role of identity-relevant stressors in HPA-axis regulation in cLBP.

## Heat- vs. mechanical-based temporal summation of pain in healthy individuals and people with neuropathic pain

Xianze Meng^1^, Rima El-Sayed^1^, Natalie Rae Osborne^1^, Vaidhehi Veena Sanmugananthan^1^, Camille Fauchon^1^, Anuj Bhatia^1^, Karen Deborah Davis^1^

^1^University Health Network (UHN)

**Introduction**: Temporal summation of pain (TSP) refers to increased pain evoked by a series of brief noxious stimuli; thought to reflect dorsal horn neuronal windup and central sensitization. Experimental studies of TSP have used heat or mechanical stimuli but it is unclear whether TSP is modality-independent. Thus, the aim of our study was to compare heat- versus mechanical-based TSP in healthy individuals (HCs) and those with neuropathic pain (NP).

**Methods**: In each HC and NP study participant, we delivered a series of 10 brief stimuli at 0.3Hz to evaluate heat (48°C, TSA II, Medoc) and mechanical (256 mN probe, MRC Systems) TSP. TSP was quantified based on % and absolute change to peak pain from the first stimulus. We conducted group and within-subject comparisons of heat and mechanical-based TSP.

**Results**: In HCs, mechanical-based TSP was significantly lower than heat-based TSP. However, in the NP group, there was no significant difference between mechanical versus heat TSP.

**Discussion/Conclusions**: These data indicate that TSP may be modality-dependent in healthy individuals but is modality-independent in people with chronic pain. The findings provide support for using mechanical devices to evaluate TSP in chronic pain which also has the benefit of simplicity and low cost compared to heat stimulus systems.

## Assessing Cannabis Use Disorder in Patients with Chronic Pain Using Medicinal Cannabis: CUDIT-R vs. Clinical Interview

Greg Tippin^1,2^, Danika Quesnel^3^, James MacKillop^2,4^, Jason Bussse^2, 4^, Vikas Parihar^1,2^, Laura Katz^2^

^1^Michael G. DeGroote Pain Clinic, ^2^McMaster University, ^3^University of toronto, ^4^Centre for Medicinal Cannabis Research (CMCR)

**Introduction**: Medicinal cannabis use is a common pain management strategy among individuals with chronic pain. There is limited research into existing tools for assessing cannabis use disorder (CUD) in chronic pain populations, and measures pre-date legalization and widespread medicinal cannabis use for chronic pain. CUD assessment in chronic pain is complicated by therapeutic cannabis use, understanding if tolerance and withdrawal reflect medical or pathological use, and high comorbidity of psychological distress. This study compared outcomes from a self-report screener and a semi-structured clinical interview in identifying CUD among patients with chronic pain and comorbid psychological distress.

**Methods**: A cross-sectional design included patients from a Canadian tertiary chronic pain clinic using medicinal cannabis for pain management. Participants completed self-report mental health measures, including the Cannabis Use Disorders Identification Test-Revised (CUDIT-R), and participated in a semi-structured clinical interview conducted by a clinical psychologist using the substance use disorder module of the Diagnostic Assessment Research Tool (DART).

**Results**: Most patients using medicinal cannabis for chronic pain did not screen positive on the CUDIT-R. Of those who did screen positive on the CUDIT-R, approximately half did not meet diagnostic criteria following clinical interview, and only one-third met criteria for CUD when tolerance and withdrawal criteria were excluded from diagnostic coding, in line with DSM-5-TR criteria. During interviewing, participants generally did not perceive their cannabis use as problematic, requiring frequent clinical judgment during semi-structured interviewing.

**Discussion/Conclusions**: Findings suggest the potential limitations of CUDIT-R in accurately identifying CUD in chronic pain populations and highlight the need for careful clinical evaluation to ensure accurate diagnosis.

## Prevalence of chronic postsurgical pain after cranial surgery: a systematic review and meta-analysis of 77 studies

Ahad Daudi^1^, Mathias Wang^2^, Ayesha Yahya^3^, Nooralhuda Alshami^4^, Niket Sampalli^1^, Gohar Zakaryan^5^, Jason Busse^6^, Li Wang^6^

^1^University of British Columbia, ^2^Hospital for Sick Children, ^3^University of Ottawa, ^4^University College Cork, ^5^University of Toronto, ^6^Department of Anesthesia, McMaster University

**Introduction**: The prevalence of chronic post-surgical pain (CPSP) after cranial surgery remains uncertain. We conducted a systematic review and meta-analysis to estimate its frequency and severity.

**Methods**: We searched MEDLINE, EMBASE, CINAHL, and Web of Science up to March 2025 for observational studies and randomized controlled trials reporting CPSP (pain lasting ≥3 months) after cranial surgery. We performed random-effects meta-analysis using Freeman-Tukey double arcsine transformation and assessed the quality of evidence using the GRADE approach.

**Results**: Seventy-seven studies (13,547 patients) met the inclusion criteria. Cranial procedures included craniotomy (52%), craniectomy (16%), and cranioplasty (10%), and mixed types (22%); and primarily for tumor resection (76%). Most studies (77%) had a high risk of bias. The median follow-up was 27.6 months (range: 3 months-16 years). CPSP of any severity was reported in 68 studies (10,785 patients) with a median prevalence of 24.6% (IQR 12.3-42.2%), moderate-to-severe CPSP (pain ≥4/10) in 24 studies (4,328 patients) with median 15.7% (IQR 10.1-21.4%), and severe CPSP (pain severity ≥7/10) in 13 studies (3,281 patients) with median 5.6% (IQR 1.7-8.6%).

Pooled prevalence estimates from meta-analyses showed moderate-certainty evidence for CPSP at any severity (27.2%, 95%CI 20.7-34.2%), moderate-to-severe CPSP (16.7%, 95%CI 12.8-20.9%), and severe CPSP (5.9%, 95%CI 3.0-9.8%).

**Discussion/Conclusions**: CPSP affects over one in four patients after cranial surgery, with one in six reporting moderate-to-severe CPSP, and 6% experiencing severe CPSP. These findings underscore CPSP as a common and under-recognized complication, highlighting the need for targeted prevention and management strategies.

## Experimental study for objective pain detection

Jinani Sooriyaarachchi^1^, Di Jiang^1^

^1^National Research Council of Canada

**Introduction**: Pain affects over 1.5 billion people worldwide and is a key symptom in numerous health conditions. Pain perception is subjective, as individuals experience pain differently even when the sources of pain are the same; therefore, self-reporting remains the standard pain assessment method in clinical practice. However, this approach is not feasible with unconscious, non-verbal and cognitively-impaired patients. Additionally, self-reported pain ratings are prone to bias, as individuals may over or underestimate, leading to potential misdiagnosis. Therefore, developing objective alternatives for pain assessment is crucial.

**Methods**: To address this challenge, we designed an experimental study to safely evoke pain in healthy adults using a Cold-Pressor-Task (CPT). During the experiment, facial videos, self-reported pain scores, and vital signs from 10 participants (5 female, 5 male) were collected. Facial expressions were analyzed using facial action coding system to identify specific facial muscle movements (action units: AUs) that correspond to facial expressions of pain. We also observed increments in self-reported pain scores and heart rate (HR) signals during CPT. Based on these preliminary findings, we developed a pain detection model using Random Forest classifier with 17 AUs and HR as input features.

**Results**: The model achieved a 10-fold cross validation accuracy of 97.65% and a hold-out test accuracy of 95.76%.

**Discussion/Conclusions**: These results demonstrate the feasibility of contactless multimodal pain detection. Once developed, such technology can enhance the quality of life for patients experiencing pain, especially those who lack access to health care, and potentially reduce the burden on the healthcare system.

## Pain as a Core Symptom in Functional Neurological Disorder: Insights From a FND Subspecialist Clinic

Adriano Mollica^1^, Abby Kazdan^1^, Orit Zamir^2^, Andrea Furlan^3^, Sarah Lidstone^4^, Matthew Burke^1^

^1^Hurvitz Brain Sciences Program, Sunnybrook Health Sciences Centre, ^2^Department of Psychiatry, Women’s College Hospital, University of Toronto, ^3^Department of Physical Medicine and Rehabilitation, Toronto Rehabilitation Institute, University Health Network, ^4^Integrated Movement Disorders Program, Toronto Rehabilitation Institute, University Health Network

**Introduction**: Chronic pain is common in functional neurological disorder (FND), but its characterization remains understudied. This study explores clinical and demographic factors associated with chronic pain in FND.

**Methods**: We retrospectively reviewed 310 consecutive patients referred to a quaternary FND neuropsychiatry clinic in Toronto, Canada. Variables included FND subtype, illness duration, disability or litigation status, active pain (body pain and/or headache), chronic pain conditions, medical and psychiatric comorbidity, and trauma history. Wilcoxon rank-sum and chi-squared tests were used for continuous and categorical variables, respectively.

**Results**: Of n=282 with complete data (mean age 40; 78% female), 63% reported active pain (26% body pain, 17% headache, and 20% both), and 42% reported a chronic pain condition. Patients with chronic pain history more q reported trauma (79.6% vs 63.4%, p=0.042) and physical triggers for FND onset (68.9% vs 38.9%, p<0.0001). Patients with active pain (n=176 vs. n=104 without pain) had longer illness duration (median 2 vs. 1.2 years, p=0.009), higher litigation rates (p=0.025), and more non-motor symptoms, including fatigue, cognitive, vestibular, and sensory symptoms (all p<0.001). Groups did not differ by age or medical/psychiatric comorbidity. Females more often reported headaches (p=0.045). Functional sensory disorders were more common with active pain (p=0.02). Body pain was more frequent in functional weakness compared with other subtypes (p=0.007), whereas headache frequency did not differ by subtype (p=0.09).

**Discussion/Conclusions**: Pain is highly prevalent in FND and associated with distinct symptom patterns, subtype distribution, trauma history, and functional severity, underscoring the need for improved phenotyping and tailored interventions.

## Pain Severity and Interference 1-Year After Open Nonmesh Inguinal Hernia Repair: A Prospective, Longitudinal Study

Marguerite Mainprize^1^, Ayse Yilbas^1^, Anton Svendrovski^2^, Joel Katz^3^

^1^Shouldice Hospital, ^2^UZIK Consulting Inc, ^3^York University

**Aim**: Determine the incidence and predictors of pain severity and interference one year after open nonmesh primary unilateral inguinal hernia repair (PUIHR).

**Methods**: Following REB approval and consent, patients scheduled for PUIHR were recruited and followed for one year. Data was collected from preoperative and postsurgical surveys and patient charts. Hernia-related pain was assessed using the Brief Pain Inventory (average severity and interference). Chronic pain in body regions unrelated to hernias or surgery was called “other” pain. Descriptive statistics and multivariate analysis were performed. P<0.05 is considered statistically significant.

**Results**: Of the 1,135 participants, 929 completed the one-year postsurgical (1YP) survey. Participants were predominantly male (95.6%) with a mean (±standard deviation) age and BMI of 58.3±13.75 years and 25±2.31 kg/m^2^. At 1YP mean average pain severity (1.43±1.44) and pain interference scores (0.53±1.07) were low, but 3.4% reported moderate-to-severe pain severity and 1.4% reported clinically important pain interference. Predictors of 1YP moderate-to-severe pain severity included other pain at 2-weeks (p=0.007) and 3 months (p=0.002), higher average pain severity at both timepoints (2-weeks:p=0.011;3-months:p<0.001), and lower 2-week resilience scores (p=0.045). Predictors of 1YP clinically important pain interference were other pain at 2 weeks (p=0.016) and 3 months (p=0.018), and higher 3-month average pain severity (p=0.041). Significance observed in preoperative and 3-day post-surgery models were isolated to individual predictors, overall model fit remained poor or nonsignificant.

**Discussion/Conclusions**: One year after open nonmesh PUIHR, 3.4% of patients reported moderate-to-severe pain severity with 1.4% experiencing clinically important levels of pain interference.

## Psychometric Evaluation of the Full and Short Versions of the Pain Catastrophizing Scale in Intensive Care Survivors in Quebec

Geneviève Laporte^1,2^, Grace Al-Hakim^1^, Bachi-Ayukokang Ebob-Anya^1,2^, Robin Kagie^2^, Michael J.L. Sullivan^3^, Céline Gélinas^1,2^

^1^Ingram School of Nursing, McGill University, ^2^Centre for Nursing Research and Lady Davis Institute, Jewish General Hospital, ^3^Department of Psychology, McGill University

**Introduction**: Pain catastrophizing is an important predictor of adverse pain outcomes, which are frequent in intensive care unit (ICU) survivors. The Pain Catastrophizing Scale (PCS: 13 items) and its short version (7 items) have been validated among chronic pain populations and its use in ICU survivors is less common. We aimed to evaluate the psychometric properties of the full and short PCS versions in ICU survivors.

**Methods**: As part of a larger cohort study, 374 adult participants (n=120 women, 32%) in five mixed ICUs in Quebec completed the PCS and other questionnaires (e.g., Brief Pain Inventory, Pain Distress, Patient Health Questionnaire-4) at discharge. At 3 months post-ICU discharge, they completed the same questionnaires as well as the Impact of Event Scale (IES-6) for post-traumatic stress symptoms (PTSS).

**Results**: Cronbach’s alphas were high (>0.90) for both PCS versions. At ICU discharge, both versions positively correlated with pain intensity (r=0.24-0.26, p<0.001), pain interference (r=0.43-0.45, p<0.001), anxiety and depression (0.48-0.50, p<0.001) and pain distress (r=0.47-0.49, p<0.001). At 3 months, they also correlated with anxiety and depression (r=0.19-0.21, p<0.01), and PTSS (r=0.30-0.31, p=0.001), but not with pain. In linear regression analysis, short and full PCS were predictive of PTSS at 3 months post-ICU (R^2^=0.09-0.10, p<0.001), but not of pain intensity (R^2^=0.01, p>0.20).

**Discussion/Conclusions**: Both short and full PCS versions showed similar psychometric properties supporting the use of the short version in the busy ICU. Our findings are consistent with those of recent COSMIN systematic review (Ikemoto et al., 2020) pertaining to the 13-item PCS.

## Biopsychosocial network in chronic low back pain

Pouya Rabiei^1,2^, Hugo Masse-Alarie^2,3^, Patrick Desrosiers^4, 5^

^1^Faculty of Medicine, Université Laval, Québec, QC, Canada., ^2^Centre Interdisciplinaire de Recherche en Réadaptation Et Intégration Sociale (Cirris), CIUSSS - Capitale Nationale, Québec, Canada., ^3^School of Rehabilitation Sciences, Faculty of Medicine, Université Laval, Québec, Canada., ^4^Département de physique, de génie physique et d’optique, 1045, av. de la Médecine, Université Laval, Québec, Canada, ^5^CERVO Brain Research Center, 2601, de la Canardière, Québec, Canada

**Introduction**: Understanding the associations among biopsychosocial factors is essential for improving research and treatment of chronic low back pain (CLBP). Here, we characterized interrelations among biopsychosocial domains using network analysis and identified the most influential features in CLBP.

**Methods**: Data came from the Quebec Low Back Pain Study, comprising 4,489 CLBP participants. We modeled relationships among baseline biopsychosocial features as networks, where nodes represent features and edges encode statistical or causal dependencies among them. Undirected network was inferred using distance correlation. Directed network was constructed using the Linear Non-Gaussian Acyclic Model, which estimates plausible causal directions. Influence maximization was performed using the Independent Cascade (IC) model to identify the most influential features in each network.

**Results**: In the undirected network, physical function and pain interference were the most central nodes, followed by depression. In the directed network, fear of movement, catastrophizing, and widespread pain emerged as key downstream hubs receiving multiple causal inputs, whereas pain interference, physical function, and depression acted as major upstream drivers exerting broad causal influence. IC diffusion simulations further identified pain interference and physical function as the most influential features in the undirected and directed networks, respectively.

**Discussion/Conclusions**: Pain interference, physical function, and depression consistently emerged as key components of the CLBP biopsychosocial network. These features exert causal effects on fear of movement, catastrophizing, and widespread pain, with diffusion analyses confirming their roles as system-wide drivers. Interventions targeting functionality and pain interference, rather than pain intensity alone, may yield broader benefits across psychological and functional domains.

## Characterization of sensory phenotype changes in cold and heat pain sensitivity following taxane-based chemotherapy in patients with breast cancer

Charles-Antoine Auger^1,2,3^, Martine Bordeleau^4,5^, Philip L. Jackson^6,7,8^, Maud Bouffard^2,3^, Frédérique Therrien^2,3,6^, Sarah Béland^2,3^, Anne Dionne^3,9,10^, Julie Lemieux^3,10^, Lucia Gagliese^11,12,13^, Jennifer S. Gewandter^14^, Lynn R. Gauthier^2,3,15^

^1^Faculty of Medicine, Université Laval, ^2^Michel-Sarrazin Psychosocial Oncology and Palliative Care Research Team, ^3^CHU de Québec-Université Laval Research Center, Oncology Division, ^4^Centre de recherche sur le vieillissement, Université de Sherbrooke, ^5^Research Group on Aging, Neurostimulation and Pain, ^6^School of Psychology, Université Laval, ^7^Centre interdisciplinaire de Recherche en Réadaptation et Intégration Sociale (Cirris), ^8^CERVO Research Center, ^9^Faculty of Pharmacy, Université Laval, ^10^Centre des maladies du sein, CHU de Québec, ^11^School of Kinesiology and Health Science, York University, ^12^Departments of Anesthesia and Psychiatry, University of Toronto, ^13^Department of Anesthesia and Pain Management, Sinai Health Systems, York University, ^14^Department of Anesthesiology and Perioperative Medicine, University of Rochester Medical Center School of Medicine and Dentistry, ^15^Department of Family and Emergency Medicine, Faculty of Medicine, Université Laval

**Introduction**: Taxane-based treatment commonly causes chemotherapy-induced peripheral neuropathy, characterized by paresthesias, pain, and altered thermal perception. One proposed mechanism involves Aδ and C fiber damage. Cold and Heat Pain Thresholds (CPT/HPT) are commonly used to assess sensory changes, but longitudinal patterns remain unclear due to reliance on unstandardized data. Z-standardization of post-treatment values relative to baseline improves detection of deviations and enables refined phenotyping.

**Aim**: Characterize CPT/HPT sensory profile changes following taxane-based chemotherapy.

**Methods**: CPT/HPT were measured at the hand/foot of 115 patients (mean age=50.56±11.18, range:24-78) before the first (T0) and after the final infusion (T1). T0 and T1-CPT/HPT values were Z-transformed using T0 data. At T0 and T1, participants were classified as “sensory gain” (Z>+1SD), “sensory loss” (Z<-1SD), or “normal” (-1SD≤Z≤+1SD). Stuart-Maxwell tests assessed overall sensory profile distribution changes; significant results (p≤0.05) were followed by McNemar tests for within-subject transitions.

**Results**: Stuart-Maxwell tests demonstrated significant changes for CPT-foot and hand (both p=0.0002), and HPT-hand (p=0.005). McNemar tests revealed both sensory gain, [19.6% of participants at the foot (p=0.009) and 14.9% at the hand (p=0.035) transitioning from T0-no sensory gain to T1-sensory gain], and sensory loss [15.2% at the foot (p=0.007) and 17.5% at the hand (p=0.004) transitioning from T0-sensory loss to T1-no sensory loss]. HPT-hand changes were non-significant.

**Discussion/Conclusions**: Findings indicate CPT sensory gain and reduced sensory loss post-treatment, suggesting heightened cold pain sensitivity. Accounting for baseline variability and tracking sensory profile transitions lays the groundwork for more precise phenotyping. Future research should refine to inform personalized, mechanism-based interventions.

## Basic Science

### La science fondamentale

## Divergent Regulation of KCC2 by PACAP and BDNF Underlies Sex Differences in Neuropathic Pain

Samuel Ferland^1^, Marc Bergeron^1^, Annie Castonguay^1^, Francesco Ferrini^1,2,3^, Yves De Koninck^1,3^

^1^CERVO Brain Research Center, ^2^University of Turin, ^3^Université Laval

**Introduction**: Downregulation of the K⁺-Cl⁻ cotransporter KCC2 in the spinal dorsal horn, and the resulting disruption of Cl⁻-mediated inhibition, is a key mechanism underlying tactile allodynia in neuropathic pain. Although this mechanism is conserved across sexes, it is mediated by BDNF/TrkB signaling in males, while the corresponding regulatory pathway in females remains unknown.

**Methods**: A cell surface biotinylation assay and chloride imaging were used to measure KCC2 levels and function in response to PACAP treatment. Immunohistochemisty was used to study the distribution and levels of PACAP, of its receptor PAC1R, and of KCC2 in naïve and nerve-injured mice of both sexes. Mice were treated with PAC1R agonists/antagonists, or a TrkB antagonist to estimate their effects on allodynia depending on sex.

**Results**: Here, we show that the neuropeptide PACAP reduces Cl⁻ transport rates. Prolonged exposure to PACAP induces KCC2 downregulation via an NMDAR-dependent pathway, indicating a PAC1R-mediated mechanism. We found females express more dorsal horn PAC1R. PACAP induced tactile allodynia, with female-specific effects at a low dose. PACAP was primarily expressed in peptidergic afferents and increased selectively in females after nerve injury. Blocking PACAP signalling restored KCC2 expression and reversed tactile allodynia in nerve-injured females, but not males. We further reveal that PACAP signaling is necessary for female-specific leptin-induced allodynia.

**Discussion/Conclusions**: Our findings uncover a novel neuroimmune signaling cascade that underlies spinal disinhibition and tactile allodynia in females. Despite immune pathway degeneracy between sexes, there is downstream convergence through chloride dysregulation, positioning KCC2 as a common target for pain therapeutics.

## Treatment of Chronic neck pain via rTMS and augmented reality sensorimotor training

Stevie Foglia^1^, Daniel Soppitt^1^, Harsha Shanthanna^2^, Zhen Gao^3^, Aimee Nelson^1^

^1^Department of Kinesiology, McMaster University, ^2^Department of Anesthesia, McMaster University, ^3^School of Computational Science & Engineering, McMaster University

**Introduction**: Chronic neck pain (CNP) reduces quality of life and is one of the leading global causes of disability. Repetitive transcranial magnetic stimulation (rTMS) has been shown to reduce chronic pain and can enhance opportunities for plasticity induced by subsequent sensorimotor training. Our lab developed ARISE, an augmented reality sensorimotor training adapted for neck pain. In this study, we pair rTMS with ARISE, to determine whether the benefits of sensorimotor training may be further enhanced by the plasticity-inducing effects of non-invasive brain stimulation. The primary objective of this study was to investigate effectiveness of rTMS paired with ARISE to reduce pain and improve function.

**Methods**: Twelve participants with CNP participated in one of two groups: 1) REAL rTMS plus ARISE or 2) PLACEBO rTMS plus ARISE. Each group received the intervention for four weeks, 3-5 sessions per week, with each session requiring ~one hour. ARISE training consisted of goal-directed cervical movements to track moving targets presented virtually. Outcomes included pain intensity, tampa scale of kinesiophobia (TSK), neck disability index (NDI), range of motion (ROM), PROMIS-29 V2.0, and patient perceived global index of change (PGIC).

**Results**: There was a similar reduction in pain intensity in the REAL and PLACEBO groups, with a clinically meaningful improvement in NDI and increase in ROM in both groups.

**Discussion/Conclusions**: This is the first study to combine rTMS with augmented reality in CNP. Results suggest improvements following REAL and PLACEBO stimulation. Further, the data suggest training with ARISE may yield improved neck mobility.

## Primary Afferent Delta Opioid Receptors Are Sufficient to Alleviate Allodynia in Chronic Pain

Heba Mohamed^1,2,3^, Louis Gendron^1,2,3,4,5^

^1^Département de Pharmacologie-Physiologie, ^2^Institut de Pharmacologie, ^3^Université de Sherbrooke, ^4^Centre de recherche du CHUS, ^5^Quebec Pain Research Network

**Introduction**: Delta opioid receptor (DOP) agonists are promising alternatives to opioids commonly used for chronic pain management due to their reduced adverse effects. DOP is widely distributed along pain pathways; however, its anatomical localization differs across species. In rodents, DOP is expressed in primary afferents and spinal cord neurons, whereas in humans it is predominantly restricted to the central terminals of primary afferents. Whether this limited distribution is sufficient to mediate analgesia in humans remains unclear. Here, we investigated whether selective expression of DOP in primary afferents is sufficient to produce antinociception.

**Methods**: Using an inducible mouse strain (*Avil*^CreERT2^; FLAG-*DOP*^fl^°^x-STOP-fl^°^x^), we generated mice expressing DOP exclusively in primary afferents, mimicking the human spinal DOP distribution. Antiallodynic effects of intrathecal deltorphin II, a selective DOP agonist, were assessed using von Frey filaments in complete Freund’s adjuvant (CFA)-induced inflammatory pain and chronic constriction injury (CCI) neuropathic pain models. Potential sex differences were also evaluated.

**Results**: Selective activation of primary afferent DOP produced robust, dose-dependent mechanical antiallodynic effects in both pain models. Importantly, these effects were comparable to those observed in mice expressing DOP in both primary afferents and spinal neurons (FLAG-DOP mice), indicating that primary afferent DOP expression alone is sufficient to alleviate allodynia. Notably, deltorphin II-induced antiallodynia showed sex dependence in the neuropathic pain model in mice expressing DOP in primary afferents.

**Discussion/Conclusions**: Together, these findings reveal that primary afferent DOP is sufficient to reduce allodynia in chronic pain states, highlighting its translational relevance as a therapeutic target for human pain.

## Revisiting the role of S1 in acute pain perception using Transcranial Focused Ultrasound (T-FUS)

Ali K. Zadeh^1^, Sandra Masoud^2^, Yuan Song^2,3^, Gabriel Pinilla-Monsalve^4^, Lucas Ronat^2,3^, Imola Mihalecz^2,3^, Samuel Pichardo^1^, Pierre Rainville^2,3^, Oury Monchi^2,3^

^1^University of Calgary, ^2^Cente de recherche, Institut universitaire de gériatrie de Montréal, ^3^Université de Montréal, ^4^University of Toronto

**Introduction**: Noninvasive neuromodulatory techniques provide important means to study the role of brain regions activated by nociceptive stimuli in pain perception. This study investigates the role of the primary somatosensory cortex (S1) and the ventral posterolateral nucleus (VPL) of the thalamus in acute pain perception using transcranial ultrasound stimulation (TUS).

**Methods**: Twenty-five healthy participants underwent a double-blind, sham-controlled, within-subject experimental design. TUS was applied to the left S1 and left VPL in separate sessions, with quantitative sensory testing (QST) performed before and after stimulation. Measures included heat pain threshold (HPT), heat pain tolerance (HPTol), warm detection threshold (WDT), mechanical detection threshold (MDT), and pressure pain threshold (PPT).

**Results**: Stimulation of the left S1 significantly lowered HPT (p = 0.013) and HPTol (p = 0.040) on the contralateral hand, with median differences of -0.6 °C (95% CI [-1.2, -0.10]) and -0.2 °C (95% CI [-1.00, 0.15]), respectively. Additionally, both S1 and VPL stimulation led to bilateral reductions in WDT (p < 0.001), with median decreases ranging from -0.25 °C to -0.35 °C (95% CIs ranging from -0.95 to 0.00). No significant changes were observed in MDT or PPT.

**Discussion/Conclusions**: These findings suggest the involvement of S1 in pain perception, particularly in modulating heat pain sensitivity. The modulation of warm detection by both S1 and VPL further suggests that TUS can influence sensory processing at multiple levels of the somatosensory pathway. Further research is needed to replicate the present findings, elucidate the underlying biophysical mechanisms and optimize stimulation protocols for clinical applications.

## Examining the association between chronic pain and tinnitus

Lise Hobeika^1^, Gianluca Guglietti^1^, Alain Londero^2^, Séverine Samson^3^, Etienne Vachon-Presseau^1^

^1^McGill University, ^2^Hôpital Lariboisière, ^3^Institut Pasteur

**Introduction**: Tinnitus is often considered the auditory analogue of chronic pain, with both conditions thought to share common mechanisms. To test this hypothesis, we examined a population cohort to determine the prevalence of various chronic pain conditions in relation to (i) tinnitus occurrence (how often individuals experience tinnitus) and (ii) tinnitus severity (how distressing the tinnitus perception is).

**Methods**: We utilized the UK Biobank dataset, which includes information on tinnitus and chronic pain from approximately 170,000 participants. We calculated the odds ratios (ORs) for different chronic pain conditions (headache, face, neck, back, stomach, hip, and knee pain) based on tinnitus occurrence and severity. Tinnitus occurrence was rated on four levels: never had, some of the time, a lot of the time, and all the time. Severity was rated as no distress, mild, moderate, or severe distress.

**Results**: Globally, The ORs for chronic pain varied based on tinnitus presence, with ORs for all chronic pain conditions ranging from [.50, .78] for individuals without tinnitus and from [1.13, 1.84] for those experiencing tinnitus at least some of the time. ORs were also linked to tinnitus severity, showing a progressive increase: ORs ranged from [.49, .78] for non-distressing tinnitus, [.97, 1.03] for mild distress, [1.30, 1.75] for moderate distress, and [1.48, 2.39] for severe distress.

**Discussion/Conclusions**: Our results demonstrate an association between chronic pain and tinnitus. Further research is needed to identify the potential environmental and biological factors underlying this association.

## Aging and chronic pain accelerate the loss of descending pain modulation in mice

Hania Oukil^1^, Laura Stone^2^, Magali Millecamps^1^

^1^Université de Montréal, ^2^University of Minnesota Twin Cities

**Introduction**: Endogenous pain modulation (ePM) can limits pain through descending pathways. In humans, ePM gradually weakens with age, typically beginning when gonadal function declines, or during chronic pain states. This study aimed to validate a novel behavioral paradigm to assess diffuse noxious inhibitory controls (DNIC) in mice and to examine the effects of aging and chronic pain on descending pain modulation.

**Methods**: Male and female mice (3 to 24 month old) were used either as healthy controls or subjected to chronic neuropathic pain induced by spared nerve injury (SNI). Descending ePM was assessed using a DNIC-like paradigm by measuring hot-plate response latency following 60s tail immersion in cold (0-1 °C) or room-temperature (20 °C) water. An increased latency after cold stimulation reflects effective descending ePM. The neuropathic phenotype was evaluated using Von Frey test (VF) and Acetone Evoked Behavior (AEB) tests.

**Results**: In both male and female, the behavioral expression of DNIC progressively declined, between 6 and 9 months of age, coinciding with reduced gonadal activity, and was no longer detectable by 12 months. Young SNI animals exhibited robust mechanical and cold allodynia associated with an absence of behavioral DNIC.

**Discussion/Conclusion**: In mice, behavioral DNIC declines in adulthood, revealing an age-related impairment of descending ePM in both sexes. Chronic neuropathic pain further exacerbates this deficit. These findings highlight the clinical relevance of the murine model for studying age- and pain-related alterations in descending pain control.

## Inflammatory monocyte-derived macrophages drive pain via their production of nerve growth factor after peripheral nerve injury in mice

Maxime Kusik^1,2^, Alexandre Paré^1,2^, Felipe Da Gama Monteiro^1,2^, Sylvain Nadeau^1,2^, Juliette Ferry^1,2^, Isabelle Pineau^1,2^, Martine Lessard^2^, Nadia Fortin^2^, Nicolas Vallières^2^, Bradley Kerr^3^, Steve Lacroix^1,2^

^1^Département de médecine moléculaire, Faculté de médecine, Université Laval, Québec, Canada, ^2^Axe neurosciences, Centre de recherche du CHU de Québec-Université Laval, Québec, Canada, ^3^Neuroscience and Mental Health Institute, University of Alberta, Edmonton, AB, Canada

**Introduction**: Immune cells are vital to regeneration and repair processes in the nervous system. We previously reported that myeloid cells play a critical role in nerve regeneration and locomotor recovery after peripheral nerve injury (PNI) by facilitating the clearance of inhibitory myelin debris, promoting angiogenesis, and producing neurotrophins (NTs) such as nerve growth factor (NGF).

**Methods**: Sciatic nerve injury was performed on male and female C57BL/6 mice. The injured nerves were collected for immune cell characterization using flow cytometry, *in situ* hybridization, or immunoblotting. Mechanical allodynia was assessed using von Frey filaments following partial sciatic nerve ligation. Supernatants from RAW264.7 macrophages, polarized into either pro- or anti-inflammatory phenotypes, were collected for NGF quantification by immunoblotting.

**Results**: NTs are synthesized by various myeloid cell types after PNI, including neutrophils, macrophages, and dendritic cells. Notably, we found that within the first week of PNI, monocyte-derived Cd11b^+^Cd68^+^Ly6B^+^Ly6C^hi^ Ccr2^+^ macrophages infiltrate the sciatic nerve in an interleukin (IL)-1-dependent manner and subsequently produce mature NGF locally. Accordingly, depletion of circulating monocytes using PLX73086, a CSF1R inhibitor unable to cross blood-nervous system barriers, reduced *Ngf* mRNA levels in the sciatic nerve distal stump. When polarized toward a proinflammatory phenotype *in vitro*, mouse RAW264.7 macrophages rapidly release the cleaved form of NGF. Further analysis revealed that systemic administration of an anti-NGF neutralizing antibody reduced mechanical pain in mice with PNI.

**Discussion/Conclusions**: These results suggest that infiltrating monocyte-derived macrophages release NGF, thereby promoting both peripheral nerve regeneration and pain following injury.

## Education

### L’éducation

## Embedding Lived Experience in ECHO: A qualitative study integrating People with Lived Experience (PWLE) in chronic pain education

Q. Jane Zhao^1^, Nitha A. Vincent^2^, Andrew J. Smith^3^, Andrea D. Furlan^1,2^

^1^University Health Network, ^2^University of Toronto, ^3^Centre for Addiction and Mental Health

**Introduction: ECHO** (Extension for Community Healthcare Outcomes) Chronic Pain is a health professions education program operating at University Health Network (UHN) since 2014. Using a hub-and-spoke model, our program aims to increase clinicians’ competency in managing chronic pain. In May 2023, a person with lived experience (PWLE) joined as a member of the expert teaching team.

**Methods**: We conducted focus group discussions (FGDs) to explore clinicians’ perspectives and experiences regarding the PWLE hub member. We analyzed the data using qualitative descriptive methods, first inductively, then deductively using Bombard’s facilitators/barriers for patient engagement. FGDs were conducted in-person, audio-recorded, and professionally transcribed. This study was approved by UHN Research Ethics Board (UHN REB#14-7415.35).

**Results**: We conducted four FGDs with 18 clinicians. We identified the following four themes regarding PWLE involvement: 1) high satisfaction, 2) opportunity for reflexivity, 3) changing group dynamics, and 4) future recommendations. Clinicians were highly satisfied and accepted the new PWLE role, with one describing them as a “good teacher.” Clinicians also reported that the PWLE perspective broadened their understanding of patient experiences, increased empathy, and supported more patient-oriented care. In terms of sessions dynamics, the PWLE’s presence altered group dynamics: clinicians became more reflective about their language and some expressed concern about unintentionally using “trigger[ing]” language. Finally, clinicians recommended increasing the diversity of PWLE who were teaching within ECHO sessions.

**Discussion/Conclusions**: Including a PWLE on the ECHO Chronic Pain hub was largely viewed positively. Recognizing PWLE as educators may deepen clinician reflexivity and empathy, supporting more patient-oriented care.

## From Guidelines to Gameplay: A Digital Serious Game to Extend Orthopedic Pain Education Home - Proof of Concept

Émilie Gosselin^1^, Charles Bilodeau^1^, Hugo Carignan^1^, Isabelle Ledoux^1^, David Labbé^2^, Hassiba Chebbihi^1^, Samia Merkhi^3^, Alain Thivierge^3^, Johanne Lapré^4^, Daphnée Ratelle^1^, Mikaël Gingras^1^, Josiane Provost^1^, Émilie Paul-Savoie^1^

^1^Université de Sherbrooke, ^2^École des technologies supérieures, ^3^CIUSSS de l’Estrie-CHUS, ^4^Patiente parrtenaire

**Aim**: To develop and assess the feasibility, relevance, acceptability, and clinical coherence of *En-Quête-Santé*, a digital serious game designed to support postoperative pain self-management for patients undergoing Enhanced Recovery After Surgery (ERAS) orthopedic procedures.

**Methods**: En-Quête-Santé was co-created using a participatory approach informed by clinical guidelines, a formal ontology of orthopedic pain management, and findings from a scoping review on digital serious games in acute care. The intervention consists of a 30-minute escape-room-style simulation delivered at home between the pre-admission visit and surgery and accessible for up to three months postoperatively. Gamification strategies include progressive challenges, immediate feedback, and integration of pharmacological and non-pharmacological pain management strategies. Iterative development was guided by a multidisciplinary steering committee including clinicians, researchers, patient partners, and video game experts. A proof-of-concept phase conducted in Fall 2025 evaluated real-world feasibility and relevance through user testing and stakeholder feedback.

**Results**: Participants completed the game independently, indicating operational feasibility. Users reported high engagement and perceived usefulness for anticipating postoperative pain-related challenges. Feedback supported clarity of content, clinical relevance, and the added value of combining playful and educational elements. Minor refinements were identified regarding navigation cues and pacing, reinforcing the importance of iterative design.

**Discussion/Conclusions**: En-Quête-Santé illustrates how a digital serious game can extend perioperative pain education beyond hospital settings while aligning with ERAS workflows. This low-burden, scalable innovation shows promise for improving patient preparedness and autonomy. A pilot randomized study is planned to evaluate clinical and implementation outcomes and inform broader adoption.

## Addressing Inequities in Sickle Cell Pain Management Through Simulation-Based Interprofessional Education

Oluwamisimi Oluwole^1^

^1^Queen’s University

**Introduction**: Sickle cell disease (SCD) is a genetic haematologic condition characterized by recurrent vaso-occlusive pain crises that require timely and adequate analgesia. Despite well-established clinical guidelines, Black patients with SCD continue to experience delayed, inadequate, and biased pain management within acute care settings. Health professional education often underemphasizes the structural and interpersonal factors contributing to these disparities.

**Methods**: A high-fidelity clinical simulation was developed for nursing and medical students to examine pain management, racial bias, and advocacy in the care of a patient presenting with a sickle cell pain crisis. The simulation required learners to assess severe pain, initiate evidence-based interventions, and navigate interprofessional communication. Debriefing emphasized reflection on bias, power dynamics, and moral distress. Qualitative and quantitative feedback were collected through pre- and post-simulation reflections.**Results**: Learners reported increased awareness of implicit bias in pain assessment, greater confidence advocating for adequate analgesia, and improved understanding of systemic barriers faced by patients with SCD. Learners identified discomfort with hierarchical escalation but recognized advocacy as a professional responsibility rather than an exceptional action. Reflections highlighted the emotional and ethical dimensions of caring for marginalized patients experiencing pain.

**Discussion/Conclusions**: Simulation-based education offers a powerful approach to addressing inequities in sickle cell pain management by integrating clinical skill development with critical reflection on bias and power. Embedding equity-centred simulations within nursing curricula may better prepare future clinicians to provide compassionate, evidence-based, and just care for patients with SCD.

## Public Information about Pain Management: an environmental scan of Canadian websites

Glenn Huang^1^, Kalee Dass^2^, Ronessa Dass^2^, Lynn Cooper^3^, Janice Sumpton^3^, Andrea Darzi^2,3^, Jason Busse^2,3^, Tara Packham^2^

^1^Hamilton Health Sciences, ^2^McMaster University, ^3^National Pain Centre

**Introduction**: Persons living with chronic pain may seek out pain management information from digital sources. We examined the accessibility and interpretability of publicly available information on opioids and cannabis for chronic pain from websites of Canadian organizations. We further captured any guidance provided by sources regarding the role of opioids and cannabis in the management of chronic pain.

**Methods**: We conducted an environmental scan of the content, accessibility, and actionability of public-facing information about opioids or cannabis use for pain. Using incognito systematic search methods, we identified Canadian websites providing guidance on the role of opioids or cannabis for adult chronic non-cancer pain. We excluded sites focusing on substance use disorders or mental health. Along with website descriptions, we captured the number of clicks required to access pain management information on opioids or cannabis from the home page and scored readability and actionability of information on 100-point scales.

**Results**: We reviewed 870 URLs and included 253 websites addressing opioids (n=126), cannabis (n=121) or both (n=6). Opioid information was largely from public organizations (68%) while commercial entities (43%) provided more information on cannabis. The average number of clicks to reach information was 4 (range 0-17). The mean reading ease was 39.1/100 at an average grade level of 12.3; the mean actionability score was 25.5/100.

**Discussion/Conclusions**: Public facing information regarding opioids and cannabis for chronic pain on Canadian websites is difficult to read, requiring high levels of literacy. Canadian websites are often unclear regarding actions that individuals can take based on the guidance provided.

## Ontological Framework for Postoperative Orthopedic Pain Management: Development and Integration into a Serious Game

Josiane Provost^1^, Émilie Paul-Savoie^1^, Daphnée Ratelle^1^, Mikael Gingras^1^, Émilie Gosselin^1^

^1^Université de Sherbrooke

**Aim**: To develop and integrate a prototype ontology for postoperative orthopedic pain management into a serious game to enhance patient self-management through structured, interoperable biomedical knowledge.

**Methods**: Following the *Minimum Information for Reporting of an Ontology* (MIRO) guidelines, we conducted: (1) a targeted review of pain management guidelines to identify key concepts related to pain assessment, pharmacological and non-pharmacological interventions, and expected outcomes; (2) conceptualization of these concepts into a hierarchical taxonomy aligned with *Open Biological and Biomedical Ontology* (OBO) Foundry standards; (3) implementation in Protégé (OWL 2.0) using a reasoner to ensure logical consistency and semantic accuracy; and (4) integration into the game engine to enable consistent, traceable, and evidence-based feedback. Expert validation with nurses, patients, and researchers is planned.

**Results**: The ontology comprises six main classes (*Patient, Pain, Assessment, Pharmacological Intervention, Non-Pharmacological Intervention, Expected Outcome*), fifteen subclasses, and twenty-two relationships. Integration within the game generated automated, guideline-aligned feedback—e.g., “ice application” linked to “moderate pain reduction” without side effects. Logical testing confirmed internal consistency.

**Discussion/Conclusions**: This ontology formalizes clinical pain management knowledge, improving the pedagogical robustness and credibility of serious games. It supports standardized educational messaging, promotes patient engagement, and may reduce opioid dependence. Its interoperability enables broader applications across digital health and clinical education systems.

## Evaluating the Effectiveness of an Online Opioid Self-Assessment Program

Andrea Furlan^1^, Sena Gok^1^, Nagina Parmar^1^, John Flannery^1^, Talia Varley^2^

^1^University Health Network, ^2^Cleveland Clinic Canada

**Introduction**: Opioid-related harms are a leading cause of drug-related mortality among North American workers, particularly those with work-related injuries like low back pain. Early opioid prescriptions are linked to longer disability durations, persistent pain, and reduced quality of life. Despite national guidelines, inconsistent prescribing practices persist, highlighting the need for targeted education.

**Methods**: To address this, the Online Opioid Self-Assessment Program was developed to improve understanding of the 2017 Canadian Opioid Guideline among prescribers, medical students, residents, and educators. This CME-accredited, self-paced program takes about three hours and includes interactive cases, quizzes, and resources across six opioid-related domains. It is available at https://opioidassessment.ca.

**Results**: Participants completed demographic surveys, a pre-test, the module, and a post-test. Of 2,031 registrants—mostly medical students—270 completed both tests. Results showed a significant increase in mean knowledge scores from 45.8% to 87.3% (Δ = 41.5, p ≤ 0.05), with consistent improvements across six-month intervals. Cumulative scores also improved from 44% to 74%, indicating a 29% gain in knowledge.

**Discussion/Conclusions: The** program effectively addressed knowledge gaps in chronic pain and opioid prescribing. Increased enrollment among medical and nurse practitioner students suggests a positive impact, especially when learners are reached early in their careers. Overall, the program demonstrates strong potential in promoting safer opioid prescribing practices and enhancing patient safety.

## Epidemiology

### L’épidémiologie

## fFrom Localized to Widespread Pain: A Spatiotemporal Model of Sensitization and Systemic Multimorbidity

Christophe Tanguay-Sabourin^1,2^, Matt Fillingim^2,3^, Jax Norman^2,3^, Azin Zare^2,3^, Lindsay Neuert^2,3^, Pierre Rainville^1,2,4^, Etienne Vachon-Presseau^2,3^

^1^Université de Montréal, ^2^Alan Edwards Centre for Research on Pain, ^3^McGill University, ^4^Centre de recherche de l’Institut universitaire de gériatrie de Montréal

**Introduction**: Persistent pain is a leading cause of global disability, yet how localized pain evolves into chronic widespread pain remains poorly defined. Sensitization is often considered a late consequence of chronicity, but its spatial and systemic progression is unclear. We hypothesized that sensitization is a graded, spatiotemporal process linking increasing pain duration and severity to broader bodily spread and multisystem involvement.

Using population-based and clinical registries, we aimed to define duration-dependent changes in pain intensity and spread, quantify multisystem comorbidity across pain states, and model back pain as a prototype of progression from localized to widespread pain.

**Methods**: We analyzed UK Biobank (n=167,000), Lifelines, MAPP, CHOIR, and the FORWARD rheumatology registry. Pain states were classified as acute, chronic, and chronic widespread. Across 981 diagnostic categories in UK Biobank, odds ratios were estimated with FDR correction. Mixed-effects models quantified associations between pain intensity and number of painful sites, and survival analyses tested whether baseline severity predicted progression.

**Results**: Longer pain duration was associated with higher intensity and broader spatial distribution across cohorts. In UK Biobank, 55% of medical conditions were associated with chronic widespread pain, compared with 41% for chronic and 8% for acute pain. Comorbidity expanded across organ systems. In back pain, greater severity correlated with broader spread and predicted faster transition to widespread pain.

**Discussion/Conclusions**: These findings indicate sensitization is an early, graded, system-wide process linking duration, intensity, and spread, positioning chronic widespread pain as a phenotype of systemic multimorbidity.

## Perspectives of Canadian Chronic Pain Providers on Burnout, Hostile Patient Behaviour, and Turnover Intentions: A National Survey

Vetri Thangavelu^1^, Rachael Bosma^2^, Brittany N. Rosenbloom^3^, Karim S. Ladha^4^

^1^Temerty Faculty of Medicine, University of Toronto, ^2^Women’s College Hospital Research and Innovation Institute, University of Toronto, ^3^Women’s College Hospital, Toronto Academic Pain Medicine Institute (TAPMI), ^4^Department of Anesthesiology and Pain Medicine, University of Toronto, Women’s College Hospital Research and Innovation Institute

**Introduction**: Chronic pain care is clinically complex and emotionally demanding, potentially increasing provider burnout, hostile patient encounters, and intention to leave practice. Canadian multidisciplinary data are limited.

**Methods**: We conducted a national cross-sectional REDCap survey of Canadian chronic pain providers, recruited through provincial pain networks and conference outreach at the 2025 Canadian Pain Society 45th annual scientific meeting. Measures included provider/practice characteristics, hostile patient encounters, the Maslach Burnout Inventory (MBI), and the Turnover Intention Scale. Burnout was classified using prespecified literature-based criteria according to MBI scoring. Descriptive and subgroup analyses used nonparametric and exact tests (α=0.05).

**Results**: Of 68 respondents, 55 completed the survey (80.9%). Burnout prevalence was 41.8% and intention-to-leave prevalence was 38.2%. Hostile patient encounters were common: monthly was the most frequent response (30.9%), 10.9% reported weekly hostility, and 12.7% reported prior physical harm by a patient. Providers with >75% proportion of their practice being in chronic pain reported more frequent hostility (p=0.029). Burnout did not differ by role (p=0.55) or learner status (p=0.686), although learners reported more frequent hostility. Intention-to-leave was associated with younger age (p=0.027) and fewer years in practice (p=0.013).

**Discussion/Conclusions**: Canadian chronic pain providers reported substantial burnout, frequent hostility, and high turnover intention. Findings suggest workforce vulnerability in early-career providers. The absence of subgroup differences in burnout despite differences in hostility exposure suggests burnout may reflect broader structural drivers beyond patient-facing conflict alone. These findings support organizational interventions focused on workplace safety, de-escalation training, and early-career retention and wellness supports.

## Performances des approches d’apprentissage automatique vs des modèles de régression traditionnels pour prédire les effets indésirables des médicaments pour la douleur chronique: Une revue narrative.

Moustapha Gassama^1^, Yohann Chiu^2^, Anaïs Lacasse^1^

^1^Université du Québec en Abitibi-Témiscamingue, ^2^Université de Sherbrooke

**Introduction**: L’apprentissage automatique (*Machine Learning*) est un domaine de l’intelligence artificielle intégrant statistique et informatique. Il permet l’analyse de données complexes. En douleur chronique, contexte marqué par la polypharmacie et une grande variabilité des réponses aux traitements, ces approches pourraient aider à prédire plus efficacement les effets indésirables. L’objectif de cette revue narrative était de synthétiser les connaissances comparant les approches d’apprentissage automatique aux modèles de régression traditionnels pour prédire les effets indésirables des médicaments utilisés dans la prise en charge de la douleur chronique.

**Méthodes**: Une revue narrative a été réalisée à partir de PubMed. Les études incluses devaient comparer explicitement un modèle d’apprentissage automatique à un modèle de régression traditionnel et rapporter des indicateurs de performance prédictive (ex. aire sous la courbe).

**Résultats**: Peu d’études comparatives sont disponibles en douleur chronique. Toutefois, en pharmacoépidémiologie, les approches d’apprentissage automatique comme les forêts aléatoires et les machines à gradient boosting obtiennent de meilleures performances que les modèles de régression logistique pour prédire les effets indésirables. Les réseaux de neurones captent bien les relations non linéaires, mais nécessitent des volumes importants de données. Les modèles hybrides, combinant puissance prédictive et interprétabilité, émergent comme prometteuses.

**Discussion/Conclusions**: Les approches d’apprentissage automatique pourraient possiblement surpasser les modèles de régression traditionnels, mais il reste encore à définir précisément dans quels contextes. Une prochaine étape de ma formation sera d’évaluer leur utilité dans un jeu de données jumelant des mesures longitudinales sur l’usage de médicaments et des données autorapportées sur les effets indésirables.

## Falling from a High: Impact of cannabis use on fall risk among adults with chronic pain seeking care at an interdisciplinary pain clinic.

Alex Chen^1^, Etienne Bisson^2,3^, Ian Gilron^1,2,3^, Rosemary Wilson^2,3,4^, Scott Duggan^1,2,3^

^1^School of Medicine, Queen’s University, Kingston, Canada, ^2^Department of Anesthesiology & Perioperative Medicine, Queen’s University, Kingston, Canada, ^3^Chronic Pain Clinic, Kingston Health Sciences Centre, Kingston, Canada, ^4^School of Nursing, Queen’s University, Kingston, Canada

**Introduction**: Cannabinoids are commonly consumed recreationally, but are also used therapeutically for chronic pain, despite unclear evidence of analgesic efficacy. The rising prevalence of cannabinoid use coupled with the known increased fall risk in patients with chronic pain necessitates investigating if there is a relationship between cannabinoid use and falls.

**Methods**: We used Kingston Health Sciences Centre Chronic Pain Registry intake data, collected between February 2024 and November 2025, for this cross-sectional comparative study. Patients reported cannabis use, falls history (past year and previous 3 months), pain characteristics, healthcare utilization, and demographic factors in a standard questionnaire (N=487). We compared cannabis-users (N=162) and non-users (N=325) to determine if there was a relationship between cannabis use and falls prevalence.

**Results**: Cannabis use was associated with an increase in fall prevalence (p<0.001). Despite being a mean of 7 years younger (95% CI=3.4-10.1), 63.0% of cannabis-users fell at least once in the past year, versus 44.6% of non-users (OR=2.11, 95% CI=1.43-3.11). Additionally, 41% of cannabis-users fell at least once in the previous 3 months versus 28% of non-users (OR=1.74, 95%CI=1.17-2.59). Cannabis-users also fell more frequently compared to non-users (p<0.001).

**Discussion/Conclusions**: Our study demonstrates novel emerging evidence of increased falls prevalence in cannabis-users versus non-users with chronic pain, accentuating the already elevated fall risk in this population. Further research on how cannabinoid use affects fall risk will improve patient safety programming and inform existing medical guidance for cannabis use in patients with chronic pain.

## Could pain sensitivity and erector spinae activation in individuals with acute low back pain explain the development of chronic pain? A secondary analysis of a randomized controlled trial

Claudia Côté-Picard^1,2^, Jean-Sébastien Roy^2,3^, Hugo Massé-Alarie^2,3^

^1^Université Laval - Faculté de Médecine, ^2^Centre interdisciplinaire de recherche en réadaptation et intégration sociale, ^3^Université Laval-École des Sciences de la réadaptation

**Introduction**: In chronic low back pain (LBP), interventions are, at best, moderately effective in decreasing pain and disability. Identifying factors contributing to chronic LBP (cLBP) development could help to improve its management. Pain hypersensitivity and altered spine motor control have been observed in patients with cLBP compared to controls. It is however unknown whether the presence of these changes at pain onset could explain cLBP development.

**Aim**: To investigate if pain sensitivity and erector spinae muscle activation could explain the transition from acute to chronic LBP.

**Methods:** This study is a secondary analysis of a randomized controlled trial including 99 participants with acute LBP. Candidate aetiological factors were pressure pain threshold (PPT), temporal summation of pain (TSP), and the flexion-relaxation ratio (erector spinae muscle activity measured at T12 - full flexion divided by trunk extension). Potential confounders were kinesiophobia, pain catastrophizing, disability level and self-efficacy measured at baseline, as well as age and sex. The outcome was cLBP 6 months after inclusion (pain ≥ 3 months and at least half the days in the last 6 months). Logistic regression models were computed for each aetiological factors, including confounders as covariates.

**Results**: No factor was significantly associated with the presence of cLBP at 6 months (PPT: OR 1.35, 95% CI 0.99 to 1.85, p=0.06; TSP: OR 0.89, 95%CI 0.59 to 1.34, p=0.6; flexion-relaxation ratio: OR 0.82 (0.06 to 11.86), p=0.88).

**Discussion/Conclusions**: Pain sensitivity and erector spinae activation did not explain the transition from acute to chronic LBP in this study.

## A User Engagement Study of Pain Severity and Mental Health Concerns in Users of the Manage My Pain App

Heather Lumsden-Ruegg^1^, James Skoric^2^, Tahir Janmohamed^3^, Quazi Rahman^4^, Joel Katz^1^

^1^York University, ^2^McGill University, ^3^ManagingLife, ^4^Trent University

**Introduction**: Digital pain management tools have potential to reduce barriers to care and empower individuals living with chronic pain. Despite growing use of pain management apps, little is known about users of these tools and how engagement patterns relate to pain severity and mental health. It is essential to understand these profiles to optimize reach and clinical impact.

**Methods**: Using a large dataset from the *Manage My Pain* app, this study analyzed the profiles of 79,806 consenting users with at least two pain records across more than one day of use. Users were classified into five clusters based on engagement patterns (longevity and number of records). Pain severity (0-10 numeric rating scale) and self-reported depression and anxiety conditions were compared across clusters using one-way ANOVA, Tukey HSD, and chi-squared tests.

**Results**: A significant difference in pain severity was observed among clusters, *F*(4, 79,801)=155.00, *p*< .001, η^2^=.008. All clusters differed significantly (*p*<.001) from one another except the highest and lowest engagement groups (*p*=.46). Two least engaged clusters reported higher pain severity than more highly engaged clusters characterized by more records (but not longevity). Mental health conditions were reported by 7.0% of users. The relationship between cluster and mental health was significant, χ^2^(8, *N*=5,585)=23.40, *p*=.003. The highest overall engagement cluster had more depression reports, whereas the low longevity but high records cluster had more combined depression and anxiety reports.

**Discussion/Conclusions**: Engagement patterns relate to pain severity and mental health, offering insight to improve interventions and outcomes for people with chronic pain.

## Polypharmacy Trajectories in Chronic Pain: Comparing Risk-Based and Count-Based Approaches Using Linked Prescription Claims Data

Hermine Lore Nguena Nguefack^1^, Nancy Ménard^2^, Sylvie Beaudoin^2^, M. Gabrielle Pagé^3^, Line Guénette^4^, Catherine Hudon^5^, Anaïs Lacasse^1^

^1^Department of Health Sciences, Université du Québec en Abitibi-Témiscamingue (UQAT), ^2^Patient partner, ^3^Research Center, Centre hospitalier de l’Université de Montréal (CHUM); Department of Anesthesiology and Pain Medicine, Faculty of Medicine, Université de Montréal, ^4^Population Health and Optimal Health Practices Research Unit, CHU de Québec Research Center - Université Laval; Faculté de pharmacie, Université Laval, ^5^Family Medicine and Emergency Medicine Departement, Université de Sherbrooke

**Introduction**: In the context of chronic pain, no study has yet compared risk-based and count-based approaches for modelling polypharmacy trajectories. This study aimed to compare a composite medication risk score, the Medication Quantification Scale 4.0 (MQS-4.0), with medication count in examining polypharmacy trajectories among individuals with chronic pain.

**Methods**: We conducted a cohort study using data from the TorSaDE Cohort, which links five cycles of Statistics Canada’s Canadian Community Health Survey (2007-2008 to 2015-2016 cross-sectional questionnaires) and Quebec provincial health administrative databases (1996-2016 longitudinal data). We selected 8,760 adults with chronic pain who were covered by public prescription medication insurance. Pain-related medications used in the 2-year period following survey completion were operationalized as monthly risk scores and monthly number of medications. Growth Mixture Modelling (GMM) was applied to each of these measures to identify groups with similar patterns over time (trajectories).

**Results**: Both the MQS-4.0 risk scores and the number of medications were effective in forming trajectory groups that were statistically robust (based on entropy and Bayesian information criterion) and clinically meaningful. However, the MQS-4.0 risk score approach was more discriminative, identifying differences between trajectory groups in terms of sociodemographic characteristics, pain profiles, health status, and healthcare utilization. Individuals with the highest numbers of pain-related medications did not necessarily have the highest MQS-4.0 risk scores, and some individuals using fewer medications were found to be at high risk.

**Discussion/Conclusions**: Our findings underscore the value of a risk-based approach such as the MQS-4.0 over a count-based approach for subgroup analysis.

## Food insecurity among adults living with chronic pain: A cross-sectional study on the prevalence and associated factors

Éloïse Farand^1^, Paul Farand^2^, Alexandra Martel^1^, Alex Dodier^3^, Valérie St-Pierre^1^, Anaïs Lacasse^4^

^1^Département de médecine de l’Université de Sherbrooke et Centre de recherche clinique du Centre intégré universitaire de santé et de services sociaux de l’Estrie - Centre hospitalier universitaire de Sherbrooke, Sherbrooke, Québec, Canada, ^2^MD, MSc, Service de cardiologie, Département de médecine de l’Université de Sherbrooke et Centre de recherche clinique du Centre intégré universitaire de santé et de services sociaux de l’Estrie - Centre hospitalier universitaire de Sherbrooke, Sherbrooke, Québec, Canada, ^3^Département de médecine de l’Université de Sherbrooke, Université de Sherbrooke, Sherbrooke, Québec, Canada, ^4^PhD, Département des sciences de la santé, Université du Québec en Abitibi-Témiscamingue, Rouyn-Noranda, Québec, Canada

**Introduction**: Food insecurity is the inability to acquire or consume an adequate diet quality or sufficient quantity of food in socially acceptable ways, or the uncertainty that one will be able to do so. Research on food insecurity among individuals living with chronic pain is limited. This study aimed to describe the prevalence of food insecurity in this population and to identify associated factors.

**Methods**: This cross-sectional study used data from 1549 participants from the CEMPUS Cohort living with chronic pain (Quebec, Canada). Participants completed a questionnaire about their health by phone or online in 2024. Food insecurity was assessed using the validated Canadian Household Food Security Survey Module (HFSSM). Multivariable logistic regression was employed to identify associated factors.

**Results**: The prevalence of food insecurity was 9.9%, with higher frequency among younger individuals, affecting 27.5%. Prevalence was similar among females and males. Severe food insecurity was observed in 2.0% of participants. Anxiety (OR: 1.125, 95% CI: 1.051-1.205), depression (OR: 1.081, 95% CI: 1.004-1.164), smoking (OR: 2.098, 95% CI: 1.234-3.566), presence of children in the household (OR: 1.927, 95% CI: 1.114-3.333), and pain duration (1-4 years vs. 3-11 months OR: 2.086, 95% CI: 1.025-4.246) were associated with higher odds of food insecurity. Older age and higher household income were associated with lower odds.

**Discussion/Conclusions**: Several potentially modifiable factors were identified, providing preliminary directions for intervention strategies. These findings highlight the importance of considering food insecurity in equity-oriented chronic pain research.

## Reaching People Living with Chronic Pain: A Cross-Sectional Study on Social Media Use and Associated Factors

Alexandra Martel^1^, Paul Farand^2^, Éloïse Farand^1^, Valérie St-Pierre^3^, Anaïs Lacasse^4^

^1^Département de médecine de l’Université de Sherbrooke et Centre de recherche clinique du Centre intégré universitaire de santé et de services sociaux de l’Estrie, Centre hospitalier universitaire de Sherbrooke, ^2^Service de cardiologie, Département de médecine de l’Université de Sherbrooke et Centre de recherche clinique du Centre intégré universitaire de santé et de services sociaux de l’Estrie, Centre hospitalier universitaire de Sherbrooke, ^3^Département d’anesthésiologie, Département de médecine de l’Université de Sherbrooke et Centre de recherche clinique du Centre intégré universitaire de santé et de services sociaux de l’Estrie, Centre hospitalier universitaire de Sherbrooke, ^4^Département des sciences de la santé, Université du Québec en Abitibi-Témiscamingue (UQAT)

**Introduction**: Social media is increasingly used for research recruitment and knowledge mobilization. However, general use of social media has not been documented among individuals living with chronic pain.

**Objectives**: This study aimed to describe social media use in this population and identify associated factors.

**Methods**: This cross-sectional study analyzed data from 1,549 participants of the CEMPUS Cohort (Quebec, Canada) who reported living with chronic pain and completed a health questionnaire (online or by phone) in 2024. Multivariable logistic regression was conducted to examine factors associated with social media use.

**Results**: Overall, 86.4% of participants (95% CI: 84.7-88.1) reported using social media. Use was higher among females (88.9%) than males (81.7%). The most frequently used platforms were Facebook (76.5%), Messenger (63.7%), and YouTube. Factors associated with greater social media use included being a female (aOR = 1.963, 95% CI: 1.390-2.771), having post-secondary education (aOR = 1.570, 95% CI: 1.106-2.229), drinking (aOR occasional vs. never = 1.635, 95% CI: 1.042-2.565), and reporting difficulties accessing healthcare (aOR = 1.499, 95% CI: 1.012-2.219). Lower likelihood of social media use was associated with older age (aOR = 0.942, 95% CI: 0.924-0.960), having chronic pain for ten years or more (aOR ≥10 vs. <1 year = 0.550, 95% CI: 0.314-0.961), and higher depression levels (aOR = 0.924, 95% CI: 0.866-0.985).

**Discussion/Conclusions**: Most participants with chronic pain used social media, indicating its potential for research recruitment and knowledge mobilization. However, exclusive reliance on these platforms may overlook certain groups, underscoring the need for diverse outreach strategies.

## Patient-Reported Characteristics and Care-Seeking Chronic Pain in Canada: A Cross-sectional Survey

Andrew Jin^1^, Maurice Zhang^2^, Navroop Liddar^2^, Sepehr Bozorgi^1^, Anais Lacasse^3^, Behnam Sadeghirad^1^, Ian Gilron^4^, Norm Buckley^1^, James Khan^5^, Manon Choinière^6^, Jason Busse^1^

^1^McMaster University, ^2^Western University, ^3^Université du Québec en Abitibi-Témiscamingue, ^4^Queen’s University, ^5^University of Toronto, ^6^Université de Montréal

**Introduction**: We aimed to characterize Canadians living with chronic pain.

**Methods**: We administered an online survey, in French and English, for Canadian adults living with chronic pain.

**Results**: We acquired 770 completed surveys, predominantly from women (90%) between 35 to 65 years of age (76%) who had been living with chronic pain for ≥15 years (51%). Average pain intensity was most often moderate (58%) or severe (24%), that interfered with daily activities very much or more (69%). Respondents were more likely to endorse frequent use of cannabis after developing chronic pain (OR 5.24; 95%CI 3.09-9.39), and less likely to endorse frequent physical activity (OR 9.36; 95%CI 6.18-14.8).

Half of respondents (52%) reported no current engagement with a health care provider for their chronic pain. Past treatments included pain clinics (96%), physiotherapy (69%), opioids (56%), complementary and alternative medicine (47%), cannabis (42%), psychotherapy (35%), and interventional procedures (22%); however, current use of any of these modalities was significantly lower (ORs ranged from 0.32 to 0.06). Most respondents indicated that treatment had resulted in ≤40% pain relief (55%) and ≤40% functional improvement (56%) and were either dissatisfied (29%) or strongly dissatisfied (24%) with treatment results.

**Discussion/Conclusions**: Respondents to our survey typically endorsed long durations of moderate to severe pain that interfered with daily activities. Treatment sought was varied and often provided limited improvement. Our findings suggest that chronic pain management remains an important public health challenge in Canada.

## Recruiting Canadian Veterans Living with Chronic Pain in the COPE-V Study: Insights, Challenges, and Lessons Learned from a Coast-to-Coast Online Survey

Anaïs Lacasse^1^, Marimée Godbout-Parent^1^, Claudie Audet^1^, Mickaël Curadeau^1^, Lise Ferland^1^, Sandra Woods^2^, Amy Doucet^2^, Anne-Marie Pinard^3^, Luc J. Hébert^3^, M. Gabrielle Pagé^4^, Pascale Marier-Deschênes^3^, Line Guénette^3^, Catherine Héroux^1^, Manon Choinière^4^, Lise Dassieu^5^, Timothy H. Wideman^6^

^1^Université du Québec en Abitibi-Témiscamingue (UQAT), ^2^PWLE, ^3^Université Laval, ^4^Université de Montréal, ^5^CIUSSS du Nord-de-l’île-de-Montréal, ^6^McGill University

**Introduction**: Recruiting Canadian Veterans living with chronic pain from coast-to-coast requires web-based strategies to overcome geographic barriers. Web-based approaches face challenges, including limited participant trust, survey fatigue, and fraudulent responses. The COPE-V study, developed in partnership with Veterans with lived-experience, aimed to build a data infrastructure supporting research on chronic pain management among Canadian Veterans.

**Methods**: COPE-V recruited English- or French-speaking Canadian Armed Forces Veterans living with pain >3 months. The survey included validated measures across the seven Veterans Affairs Canada well-being domains and the Canadian adult pain registry minimum dataset. Recruitment (May-Oct 2025) leveraged Veteran and patient organizations, social media, and outreach to equity-deserving groups. Data quality was maximized via a honeypot item, timing checks, duplicate detection, and careful analysis of inconsistent data.

**Results**: Recruitment efforts yielded 505 responses; 173 were excluded due to empty questionnaires (19.2%), bots (14.7%), or duplicates (0.4%), leaving 332 valid participations. Successes included engagement with diverse organizations and broad geographic representation; 22.9% identified as women, 20.7% as Indigenous, and 46.1% responded in French. High interest was observed, with 89.2% open to future team projects. A key strategy was to humanize the survey with photos/videos, branding, and a compassionate approach to well-being. Challenges included recruitment (~300hrs), fraudulent responses, and a number of organizations did not respond. Some former military personnel did not identify as ‘Veterans,’ prompting varied outreach strategies.

**Discussion/Conclusions**: Despite challenges, the COPE-V study successfully engaged a diverse and committed sample of Veterans, highlighting strong potential for future research on chronic pain management.

## Healthcare utilization of Ontario Veterans with and without back pain: A population-based cohort study

Zhikang Ye^1^, Jessica Wong^2^, Jason Busse^1^, Rebecca Griffiths^3^, Keven Phinney^4^, Andrew Thomas^4^, Tom Hoppe^4^, Duncan Redburn^4^, Alyson Mahar^5^

^1^McMaster University, ^2^Western University, ^3^ICES, Ontario, ^4^Chronic Pain Centre of Excellence for Canadian Veterans, ^5^Queen’s University

**Introduction**: Veterans are disproportionately affected by back pain, yet care-seeking associated with this condition remains poorly understood.

**Methods**: We conducted a population-based historical cohort study of veterans residing in Ontario, Canada. We identified veterans through a Ministry of Health-supplied list of anonymized individuals with an administrative veteran identifying code between April 1, 2002 - Mar 31, 2021, linked to health administrative data held at ICES (formerly the Institute for Clinical Evaluative Sciences). Exposure was back pain, defined as ≥1 healthcare encounter for back pain within 2 years of the index date. We assessed associations between back pain and all cause and mental health-related healthcare utilization up to 5-year follow-up using multivariable Andersen-Gill recurrent event regression models. We used interaction terms to explore whether associations between back pain and healthcare utilization varied by age, sex, mental health comorbidity, and length of service.

**Results**: Among 17,756 veterans, 3,714 sought cares for back pain. Compared to veterans without back pain, veterans with back pain care were more likely to incur all-cause outpatient visits (adjusted hazard ratio [aHR] 1.46, 95% CI, 1.41-1.52), emergency department visits (aHR 1.54, 95% CI, 1.44-1.66), and inpatient visits (aHR 1.40, 95% CI, 1.24-1.57). Similarly, veterans with back pain were more likely to attend for mental health-related outpatient visits (aHR 1.42, 95% CI, 1.27 - 1.59), emergency department visits (aHR 1.38, 95% CI, 1.05 - 1.82), inpatient visits (aHR 1.55, 95% CI, 1.09 - 2.20). The relationship between back pain and all cause outpatient visit rates was greater among veterans with mental health comorbidity (p=0.001), older age (p=0.022) and longer length of service (p=0.020).

**Discussion/Conclusions**: Back pain among veterans was associated with greater all-cause and mental health-related healthcare utilization. Differences by sex, mental health comorbidity, and service duration highlight the need for subgroup-specific management strategies.

## Evidence, systematic reviews, guidelines, implementation science

### Les données probantes, les revues systématiques, les recommandations, la science de la mise en œuvre

## Integrating Patient Partners with Lived Experience into Pain Research and Clinical Trials: Benefits and Effective Approaches

Lucy Kovalova-Woods^1,2^

^1^CAPA, ^2^WKG Foundation

**Introduction**: Chronic pain is a complex, subjective, and frequently invisible condition that significantly affects quality of life, daily functioning, and healthcare utilization. Despite its widespread impact, pain research and clinical trials have historically been designed with limited involvement from individuals living with pain. This lack of lived-experience input can reduce the relevance, feasibility, and real-world applicability of research outcomes. Increasingly, the meaningful inclusion of patient partners, individuals with lived experience of chronic pain, is recognized as essential for improving the quality, ethics, and translational value of pain research and clinical trials.

**Methods**: This work draws on observations and practical experiences from patient-engaged pain research initiatives, alongside existing literature on patient partnership in clinical research. Models of engagement were examined across multiple stages of the research lifecycle, including research question development, study design, recruitment strategies, outcome measure selection, and knowledge translation activities. Key engagement strategies analyzed included early involvement of patient partners, clearly defined roles and expectations, equitable compensation, accessible communication practices, and training for both researchers and patient partners.

**Results**: Patient partner involvement provided valuable experiential insights that complemented scientific and clinical expertise. Engagement contributed to improved prioritization of research questions, greater alignment between outcome measures and patient priorities, and enhanced accessibility of study design. Studies that incorporated patient perspectives demonstrated improved recruitment and retention, more culturally sensitive approaches, and stronger relevance to real-world patient needs. Additionally, patient partners played a critical role in knowledge translation by helping to communicate research findings in accessible and meaningful ways.

**Discussion/Conclusions**: Meaningful patient engagement requires more than symbolic inclusion. Effective partnerships depend on early and continuous involvement throughout the research lifecycle, supportive and respectful collaboration environments, and recognition of experiential knowledge as legitimate expertise. Flexible participation options, trauma-informed approaches, and attention to equity and diversity are particularly important in chronic pain research due to stigma, disability, and fluctuating health status among participants. Embedding patient partners as collaborators rather than consultants represents a significant shift toward more ethical, inclusive, and impactful pain research. Such partnerships strengthen scientific rigor and increase the likelihood that research findings will translate into interventions that meaningfully improve the lives of people living with chronic pain.

## “It isn’t the brain that hurts”: A Qualitative Empirical Bioethics Investigation of Chronic Pain, Brain Disease Models, Objectivity, and Stigma

Iris Coates McCall^1^, Marnie Cornett^1^, Brooke Magel^1^, Rachael Bosma^2^, Emeralda Burke^1^, Jennifer Chandler^3^, Zahra Hasan^4,5^, Chris Lo^6,7,8^, Javeed Sukhera^9^, Karen Davis^10,11,12^, Daniel Buchman^1,6,13^

^1^Centre for Addictions and Mental Health, Toronto, ON, ^2^Women’s College Hospital, Toronto, Canada, ^3^Faculty of Law, University of Ottawa, Ottawa, Canada, ^4^North York General Hospital, Toronto, Canada, ^5^Sunnybrook Health Sciences Centre, Toronto, Canada, ^6^Dalla Lana School of Public Health, University of Toronto, Toronto, Canada, ^7^Department of Psychiatry, Temerty Faculty of Medicine, University of Toronto, Toronto, Canada, ^8^School of Social and Health Sciences, James Cook University, Singapore, ^9^Department of Psychiatry, Hartford Hospital, Hartford HealthCare, Hartford, USA, ^10^Krembil Brain Institute, University Health Network, Toronto, Canada, ^11^Department of Surgery, Temerty Faculty of Medicine, University of Toronto, Toronto, Canada, ^12^Institute of Medical Science, Temerty Faculty of Medicine, University of Toronto, Toronto, Canada, ^13^University of Toronto Joint Centre for Bioethics, Toronto, Canada

**Introduction**: Chronic pain is highly stigmatized and morally fraught. Because it is invisible and lacks an objective diagnostic test, people living with chronic pain often face distrust and doubt. Advances in neuroimaging have identified brain-based biomarkers associated with chronic pain, prompting proposals for a brain disease model of chronic pain (BDM-CP). Proponents argue that framing chronic pain within an objective biomedical model could reduce stigma. However, research on other brain disease models shows they can lessen some forms of stigma while worsening others. Whether a neuroimaging-supported BDM-CP reduces stigma has not been empirically examined.

**Methods**: We conducted a qualitative study using thematic analysis of semi-structured interviews with 47 adults living with chronic pain in Canada to explore how a BDM-CP might affect stigma.

**Results**: Participants differed in their opinions regarding the impact of a BDM-CP and neuroimaging on stigma, reporting each can be both validating *and* invalidating, stigmatizing *and* destigmatizing, depending on the context and across different relational dimensions. Participants expressed that a BDM-CP could legitimize their experiences, but expressed concern that localizing the disease process in the brain could reinforce negative stereotypes associated with mental illness or alter their sense of identity. Analysis revealed concerns relating to stigmatizing associations with severity, permanence, and “false negative” neuroimaging results, with clinical, interpersonal, professional, and systemic domains being relevant to these differential impacts.

**Discussion/Conclusions**: Although a BDM-CP may offer anti-stigma benefits, not all individuals endorse this framing, and unintended negative consequences may arise if diverse perspectives are not carefully considered.

## Toward Equity-Oriented Knowledge Mobilization in Chronic Pain Research: A Scoping Review of EDI-D and Patient Engagement Integration

Jaklyn Andrews^1^, Olayinka Ariba^1^, Karime Mescouto^1^, Emerald Asuncion^1^, Megan Harley^2^, Laura Connoy^1^, Maren Goodman^1^, Fiona Webster^1^

^1^Western University, ^2^Toronto Metropolitan University

**Introduction**: Chronic pain is shaped by structural inequities related to race, gender, disability, socioeconomic status, and colonial histories. Within knowledge mobilization (KM), approaches that are being implemented to rectify these inequities include innovative patient engagement (PE) strategies and practices related to equity, diversity, inclusion, and decolonization (EDI-D). Still, their integration within chronic pain research remains unclear.

**Objective**: To map the current landscape of literature integrating EDI-D and PE principles within KM, and to examine implications for chronic pain research and engagement.

**Methods**: Guided by the scoping review framework outlined by Arksey and O’Malley (2005) and refined by Levac et al. (2010) and the PRISMA-ScR (2018) reporting checklist, we conducted a comprehensive search of academic databases and grey literature published in the last ten years. 40 sources were included in the study and underwent structured data extraction. Descriptive and thematic analyses explored how KM, PE, and EDI-D are conceptualized, operationalized, and evaluated.

**Results**: Cross-cutting preliminary themes include: (1) how power is distributed within KM partnerships; (2) the need for system transformation to meaningfully integrate, rather than subordinate, lived expertise; (3) tensions between positioning EDI-D as foundational versus a component of KM initiatives; (4) accessibility as a central mechanism of equitable KM; and (5) limited formal evaluation of equity-oriented KM efforts. Few publications addressed chronic pain populations or contexts.

**Discussion/Conclusions**: Despite well-documented inequities in chronic pain care and research, equity-oriented KM approaches remain underdeveloped in this field. Chronic pain research presents an important opportunity to advance, operationalize, and evaluate equitable KM practices.

## How do graded exposure, graded exercise, or graded activity differ in chronic pain rehabilitation?

Vivian Hu^1^, Ronessa Dass^1^, Janet Holly^1^, Rebecca Guse^1^, Takara McNeil^1^, Tara Packham^1^

^1^McMaster University

**Introduction**: Graded exposure (GExp), graded activity (GA), and graded exercise (GE) are related approaches used in chronic pain rehabilitation to reduce disability. However, disciplinary distinctions in how these interventions are defined, operationalized, and evaluated across occupational therapy, physiotherapy, and psychology lacks clarity. We hypothesize that mapping these approaches will allow us to clarify these distinctions across rehabilitation professions.

**Methods**: This concept analysis used scoping review methodology to locate papers describing the conceptual elements of GExp in persons with chronic pain. We systematically searched PsychINFO, CINAHL, and Medline, including papers using any study design, in English, and focusing on chronic pain interventions. Abstracts and full text were reviewed by two independent reviewers for inclusion and reported using a PRISMA diagram. Data was extracted on conceptual elements including definitions, antecedents, mechanisms, and outcome measures; then synthesized using qualitative descriptive content analysis.

**Results**: Of the 113 papers identified, the majority were in physiotherapy (n=80), or psychology (n=36). GExp was the most referenced term as a singular approach (n=60), and GE was the least referenced (n=16). There were multiple papers describing all disciplines and combining approaches: GA and GExp were paired the most often. Across professions, common antecedents identified were pain-related fear, activity avoidance, and nociplastic pain.

**Discussion/Conclusions**: This concept analysis synthesized definitions for graded exposure-related approaches, highlighting nuanced disciplinary differences between GExp, GA, and GE; and mapping how these approaches are used across rehabilitation professions. We anticipate this will clarify GExp in clinical practice and research.

## The benefits and harms of physical activity for patients with myalgic encephalomyelitis/chronic fatigue syndrome: a systematic review and meta-analysis of randomized trials

Sarah Kirsh^1,2^, Oswin Chang^3^, Michael Ling^1^, Tanvir Jassal^1^, João Pedro Lima^1,2^, Mahsa Raji Lahiji^2^, Rachel Couban^1^, Dena Zeraatkar^1,2^, Jason Busse^1,2^

^1^Department of Anesthesia, McMaster University, ^2^Department of Health Research Methods, Evidence, and Impact, McMaster University, ^3^Faculty of Medicine, University of British Columbia

**Introduction**: Myalgic encephalomyelitis/chronic fatigue syndrome (ME/CFS) is a condition characterized by persistent fatigue, post-exertional malaise, cognitive dysfunction, and unrestful sleep. For decades, guidelines recommended graded physical activity (GPA) to improve patients’ physical capacity, though concerns about post-exertional malaise, a defining characteristic of ME/CFS, have led many to question its safety and effectiveness.

**Objective**: To systematically review and summarize evidence from randomized trials on the benefits and harms of physical activity for adults with ME/CFS.

**Methods**: We searched MEDLINE, EMBASE, PsycInfo, and CENTRAL from inception to July 2024 for randomized controlled trials comparing physical activity with usual care, pacing, or cognitive and behavioral interventions in adults diagnosed with ME/CFS. Reviewers worked independently and in duplicate to screen records, extract data, and assess risk of bias. We performed random-effects meta-analyses and rated the certainty of evidence using the GRADE approach.

**Results**: Nineteen trials (37 records; n=2,684) proved eligible. Moderate certainty evidence suggests that GPA probably reduces fatigue compared with control (mean difference [MD] -5.30, 95% CI -8.89 to -1.72) and probably decreases post-exertional malaise (risk difference [RD] -17.2%, 95% CI -25.8% to -7.9%). Moderate certainty evidence suggests that traditional Eastern movement therapies probably reduce fatigue (MD -2.67; 95% CI -3.67 to -1.67).

**Discussion/Conclusions**: Graded exercise and Eastern movement therapies likely improve fatigue in adults with ME/CFS. These findings will inform forthcoming guideline recommendations on the management of ME/CFS.

## Efficacy and Safety of Stellate Ganglion Blocks in the Treatment of Chronic Pain: A Systematic Review of Randomized Control Trials.

Lucas Zou^1^, Alex Chen^1^, Etienne Bisson^2,3^, Scott Duggan^2,3^, Ian Gilron^2,3^

^1^School of Medicine, Queen’s University, Kingston, Canada, ^2^Department of Anesthesiology & Perioperative Medicine, Queen’s University, Kingston, Canada, ^3^Chronic Pain Clinic, Kingston Health Sciences Centre

**Introduction**: Chronic pain (CP) is a highly debilitating condition that drastically reduces quality of life for those living with it despite modern multimodal therapy. The use of stellate ganglion block (SGB) in CP declined with the advent of newer analgesic approaches, but with emerging evidence suggesting that sympathetic overactivation underlies CP maintenance, there is renewed interest in SGBs as an adjuvant therapy. However, data supporting their efficacy, indications, and safety remain limited. An evidence-based review of SGBs used for CP conditions is therefore needed to inform clinical use and guide future research.

**Methods**: We searched for randomized control and crossover trials utilizing pharmacological and non-pharmacological SGB in CP patients. Studies were excluded if they did not include non-SGB controls or permanently ablated the stellate ganglion. The main outcomes were analgesic efficacy, interpreted through pain scores pre/post intervention, as well as adverse events.

**Results**: 15 trials (n=734) investigating SGB for CP were included. Aetiologies of CP ranged from complex regional pain syndrome to postherpetic neuralgia, among others. Preliminary data show that of 6 trials comparing SGB to sham procedures, 4 demonstrate equivalence of SGB to placebo for pain relief. Of the remaining 9 comparing SGB to medical/interventional modalities, 8 show that the analgesic effects of SGB are non-superior to the comparator.

**Discussion/Conclusion**: The implementation of SGBs for CP in Canadian pain clinics is irregular and often at the discretion of individual physicians. This review consolidates the evidence supporting their indications to better inform and standardize medical practice for pain clinicians.

## Complementary and Alternative Medicine (CAM) for Post-Cesarean Pain Management: A Rapid Review of Current Recommendations

Daphnée Ratelle^1^, Émilie Gosselin^1^, Josiane Provost^1^, Mikaël Gingras^1^, Émilie Paul-Savoie^1^

^1^Université de Sherbrooke

**Introduction**: Cesarean section is the most common surgery throughout Canada, with rates rising over the past 20 years. Severe pain after cesarean affects more than half of patients and contributes to physical, psychological, and parental complications. Current pain management practices vary widely across hospitals, and some effective strategies remain underused due to concerns about maternal or neonatal safety. Zimpel et al.’s review of complementary and alternative medicine (CAM) for pain management following cesarean was conducted in 2020, highlighting the need for an updated review.

**Objectives**: To conduct a rapid review of recent literature (past 5 years) on non-pharmacological pain treatment after cesarean.

**Methods**: This rapid review was conducted in accordance with the Cochrane Rapid Reviews Methods Group (2024), by replicating the search strategy of Zimpel et al. (2020) with the assistance of a librarian. Using PICO, 7 databases were searched, with outcomes restricted to “pain” and “adverse effects”. Two reviewers screened 20% of articles (agreement: κ ≥0.8), followed by single screening until completion. Data was extracted by a single reviewer and verified by another member of the team.

**Results and Conclusions**: Of the 872 screened studies, 44 were included. Several methods examined by Zimpel et al. (2020) are now supported by additional evidence, while new approaches have emerged, including but not limited to abdominal binders, Su Jok seed therapy, gum chewing, and Kinesio taping. This synthesis will support clinicians to update and adapt current recommendations for CAM to help reduce the prevalence of post-cesarean section pain through alternative methods.

## Evidence in Action: What We Learned from an Environmental Scan of Pediatric Pain Tools and Resources

Megha Rao^1^, Justin Bonhomme^2^, Louise Tunnah^3^, Sofia Olaizola^3^, Kathryn Birnie^1^

^1^University of Calgary/Alberta Children’s Hospital/Solutions for Kids in Pain (SKIP), ^2^University of Calgary/Alberta Children’s Hospital, ^3^Solutions for Kids in Pain (SKIP)/University of Dalhousie

**Introduction**: Pediatric pain remains pervasive and inequitably managed in hospital settings with gaps in access to, and availability of, resources sharing evidence-based information with patients, families, health professionals, and decisionmakers. This environmental scan served to identify and consolidate existing resources and identify gaps for new resource development.

**Methods**: Applying Shahid and Turin’s (2018) methodological framework, strategies included consultation with content experts, and targeted website and customized Google searches conducted until January 2026. Eligible resources were: (1) evidence-based and created by a government or recognized institution/organization; (2) free/open-access; (3) in English and/or French; (4) aligned with the Pediatric Pain Management health standard; (5) focused on pain in children (<18 years); and (6) current within the past 10 years. Resources were coded based on type, target audience, content, child age, type of pain, pain management strategies, and quality.

**Results**: A total of 455 resources were identified, diverse in format (e.g. toolkits, training, videos, infographics) and context (e.g. inpatient, rehabilitation). The majority of resources targeted health professionals (n=238), focused on procedural pain (n=270), and pain in children (6-12 years; n=136) and/or adolescents (13-18 years; n=149). Psychosocial (n=313), physical (n=280), and pharmacological strategies (n=257) were all represented. Resource gaps included children with neurodevelopmental disabilities, including those nonverbal and diagnosed with rare diseases.

**Discussion/Conclusions**: This environmental scan provides foundational evidence to advance equitable implementation of quality pediatric pain care. By identifying resources and critical gaps, this initiative will inform co-created implementation supports and strengthen system-level change to deliver high quality pain care for all children.

## Methylene blue for acute and chronic pain management: a systematic review and meta-analysis

Victoria Chan^1^, Alexander Xiang^2^, Jordan Vaarsi^3^, James Khan^4^

^1^Department of Anesthesiology and Pain Medicine, University of Toronto, Toronto, Canada, ^2^DeGroote School of Medicine, McMaster University, Hamilton, Canada, ^3^School of Medicine, Royal College of Surgeons In Ireland, Dublin, Ireland, ^4^Mount Sinai Hospital, Department of Anesthesia and Pain Medicine, University of Toronto, Toronto, Canada

**Introduction**: Methylene Blue (MB) has been widely utilized in medicine, has anti-oxidant and anti-inflammatory properties and is used to manage both acute and chronic pain. However, its analgesic efficacy remains uncertain. This systematic review and meta-analysis evaluated the efficacy and safety of methylene blue in the management of chronic pain.

**Methods**: A systematic search was conducted using a librarian-approved search strategy. Primary outcomes included pain scores post-MB administration, patient satisfaction, and post-procedure analgesic use. Secondary outcomes included functional capacity, complication rates, and ability to perform daily activities. A meta-analysis was performed on pain scores of randomized controlled trials (RCTs) and reported using mean differences (MD) and 95% confidence intervals (CI). A random-effects model was used, and heterogeneity assessed using the I^2^ statistic.

**Results**: 10 RCTs (n=647) and 12 observational studies (n=549) were included. Eight RCTs showed positive findings on pain scores, three for improved function, four for reduced analgesic consumption, and three for improved sleep quality. MB showed significant reduction in pain scores at one (MD -1.13, 95% CI -1.66 to -0.61, I^2^=70%) and six months (MD -1.71, 95% CI -3.14 to -0.28, I^2^=96%) post-treatment; although the three-months pooled analysis did not show a difference (MD -0.98, 95% CI -2.15 to 0.20, I^2^=97%). Statistically insignificant adverse effects were hyperglycemia, nausea, hypertension and dizziness post-MB interventions.

**Discussion/Conclusions**: MB can be helpful in providing short (one-month) and long-term relief (six-months) for interventional chronic pain management. However, further larger high-quality RCTs investigating the safety and effectiveness of MB on chronic pain is needed.

## Effectiveness of sucrose for venipuncture analgesia in neonates: systematic-review and metanalyses

Mariana Bueno^1^, Ligyana Candido^2^, Jiale Hu^3^, Michelle Fiander^4^, Jane Cracknell^5^, Emily Xu^1^, Jiamin Kang^6^, Janet Yamada^7^

^1^Univesity of Toronto, ^2^Univesity of Ottawa, ^3^Virginia Commonwealth University, ^4^Cochrane Neonatal Group, ^5^Vermont Oxford Network, ^6^Xuzhou Medical University, ^7^Toronto Metropolitan University

**Aim**: To evaluate the effectiveness of sucrose analgesia for venipuncture in neonates.

**Methods**: We searched Cochrane CRS, MEDLINE, and Embase (July 2025); and China National Knowledge Infrastructure (CNKI), VIP Chinese Science and Technology Periodicals, and Wanfang Data (July 2024). Pain intensity scores were reported as mean differences (MD), standardized MD (SMD) or relative risk (RR) with 95% confidence intervals (CI). GRADE was used to assess certainty of evidence.

**Results**: 29 studies (2,764 infants) were included, with large variations in sucrose doses, concentrations, and mode of administration. Compared to control (1), sucrose with or without non-nutritive sucking (NNS) probably reduced pain during and 30 seconds after venipuncture (SMD -0.82, 95%CI -1.02,-0.63; 7 studies, n =477; moderate-certainty evidence); (2) sucrose with NNS reduced pain one minute after venipuncture (MD -9.15, 95%CI -9.91,-8.39; 1 study, n=100; high-certainty evidence). Compared to breastfeeding, sucrose probably reduced pain during venipuncture (RR 1.38, 95%CI 1.01,1.88; 1 study, n=103; moderate-certainty evidence). Compared to NNS, (1) sucrose with NNS likely reduced pain during venipuncture (SMD -1.52, 95%CI -1.92,-1.12; 2 studies, n=136; moderate-certainty evidence); (2) sucrose with NNS probably reduced pain 2 minutes after venipuncture (SMD -1.3, 95%CI -1.71,-0.89; 2 studies, n=136; moderate-certainty evidence). The evidence is low to very-low certainty for other comparators, including skin-to-skin, EMLA, feeding, glucose, and positioning. Further comparisons were precluded due to the large variability in pain measures reported, comparator groups, and limited data available for meta-analyses.

**Discussion/Conclusions**: There is moderate to high-quality evidence to support the effectiveness of sucrose for venipuncture analgesia in neonates.

## Determinants and stage-specific processes for implementing Adaptive Mentoring Networks to strengthen chronic pain care

L. Jayne Beselt^1^, Zack van Allen^2^, Laura Harris-Lane^3^, Michelle Moussa^2^, Joshua Rash^4^, Douglas Archibald^1^, Jerry Maniate^2^, Samuel Hickcox^5^, Cristian Rangel^2^, Arun Radhakrishnan^2^

^1^bruyere health research institute, ^2^University of Ottawa, ^3^York University, ^4^Memorial University of Newfoundland, ^5^Nova Scotia Health Authority

**Introduction/Aim**: Adaptive mentoring networks (AMNs) may build primary care capacity and provider well-being for complex chronic pain care, but determinants of successful cross-setting implementation are not well described. This project aims to identify cross-network determinants and stage-specific implementation processes to inform an implementation blueprint and strategies for spread and scale of AMNs.

**Methods**: A retrospective document review and key-informant interviews were conducted across eight Canadian AMNs. Data were synthesized using Consolidated Framework for Implementation Research (CFIR) and mapped to Active Implementation Framework (AIF) stages; modifiable CFIR barriers were mapped to Expert Recommendations for Implementing Change (ERIC) strategies using CFIR-ERIC matching.

**Results**: CFIR synthesis highlighted facilitators (credible innovation source, relative advantage, adaptability/trialability, partnerships, relational connections, communication and learning culture, and supportive implementation processes) and common challenges (innovation complexity and coordination burden, patchwork resourcing and cost pressures, critical incidents, and policy or legal constraints). AIF activities were iterative: in exploration, networks adapted models to local and equity needs and established governance and legitimacy; in installation, they built operational structures for co-design, recruitment, training, and measurement; in initial implementation, they delivered regular mentoring, monitored outcomes, and used rapid-cycle improvement to scale; and in full implementation, they supported controlled growth and fidelity through ongoing evidence generation and sustainable resourcing. CFIR-ERIC matching generated a draft menu of candidate strategies tailored by stage and context.

**Discussion/Conclusions**: Findings inform a pragmatic blueprint to support AMN spread and scale. These will translate into a stage-based playbook to guide ERIC-aligned strategies for emerging and scaling networks.

## Implementation Determinants of Project ECHO Chronic Pain Programs in Canada

Monika Kataria^1,2^, Q Jane Zhao^1^, Yalnee Shantharam^1^, Chitra Lalloo^3,4^, Xiaolin Wei^4,5^, Lori S. Montgomery^6^, Helena Daudt^7^, Jean-François Leroux^8^, Andrea D. Furlan^1,2,9^

^1^Toronto Rehabilitation Institute, University Health Network, Toronto, Canada, ^2^Institute of Medical Science, Temerty Faculty of Medicine, University of Toronto, ^3^Child Health Evaluative Sciences, The Hospital for Sick Children, Toronto, ON, Canada, ^4^Institute of Health Policy Management and Evaluation, University of Toronto, Toronto, ON, Canada, ^5^Dalla Lana School of Public Health, University of Toronto, Toronto, ON, Canada, ^6^Departments of Family Medicine and Anesthesiology, Perioperative and Pain Medicine, Cumming School of Medicine, Calgary, AB, Canada, ^7^Pain Canada, Pain BC, Vancouver, BC, Canada, ^8^Chronic Pain Policy Team, Controlled Substances and Cannabis Branch, Health Canada, Ottawa, Ontario, Canada, ^9^Institute for Work & Health, 481 University Avenue, Suite 800, Toronto, ON, Canada

**Introduction**: Chronic pain affects approximately 1 in 5 Canadians. Most chronic pain patients are managed by family physicians receiving minimal training in pain management. Project Extension for Community Healthcare Outcomes (ECHO) is a virtual model to increase capacity to manage chronic pain in community settings by connecting primary care providers with specialists. Although qualitative evidence from Québec has described implementation processes across multiple ECHO clinical areas, little is known about barriers and facilitators specific to the implementation of ECHO chronic pain programs across Canada.

**Methods**: A REDCap survey based on the Consolidated Framework for Implementation Research (CFIR 2.0) was administered across 12 ECHO chronic pain hubs in Canada. One staff member per hub completed the survey assessing implementation determinants across the 5 CFIR domains. Items were rated on a 7-point Likert scale and construct level mean scores across all 12 hubs were mapped onto the CFIR −2 to +2 rating scale (−2 = strong barrier to +2 = strong facilitator).

**Results**: Within the intervention characteristics domain, all constructs were rated as facilitators except for cost, which was rated as a weak barrier. Inner setting constructs were rated as weak facilitators. Within the outer setting domain, constructs were weak facilitators except for external policies, which was rated neutral. Within the characteristics of individuals domain, knowledge, beliefs and self-efficacy were rated as strong facilitators, session timing was rated as neutral and remaining constructs were weak facilitators. Implementation process constructs were rated as weak facilitators.

**Discussion/Conclusions**: Overall, implementation of Project ECHO chronic pain programs in Canada was supported by strong perceptions of effectiveness and confidence among those delivering the programs. In contrast, financial constraints emerged as a barrier. Organizational and process related factors generally provided limited support. Hence, efforts should reinforce knowledge and self-efficacy, prioritize sustainable funding and stronger organizational and implementation support.

## Combination Pharmacotherapy for the Treatment of Fibromyalgia in Adults: A Systematic Review and Meta-Analysis Update

Shokouh Abolhosseini^1^, Landon Montag^2^, Tim Salomons^1^, Ian Gilron^3^

^1^Department of Psychology, Queen’s University, Kingston, ON, Canada, ^2^School of Medicine, Queen’s University, Kingston, ON, Canada, ^3^Department of Anesthesiology and Perioperative Medicine, Queen’s University, Kingston, ON, Canada

**Introduction**: Fibromyalgia is a chronic condition characterized by widespread pain and impaired sleep, mood and cognitive function, affecting 2-8% of people. Current pharmacological monotherapies provide limited relief, with meaningful symptom reduction in only a subset of patients. Combination pharmacotherapy is commonly used in clinical practice to improve outcomes; however, evidence supporting its efficacy and safety remains unclear. This systematic review update investigated the efficacy and tolerability of combination treatments compared with monotherapy or placebo in adults with fibromyalgia.

**Methods**: MEDLINE, EMBASE, and CENTRAL were searched from Sep-2017 to Dec-2025. Eligible studies were double-blind RCTs comparing combination pharmacotherapy with monotherapy or placebo. Outcomes included pain, quality of life, sleep, fatigue, mood, and adverse events. Qualitative synthesis and meta-analysis were conducted where appropriate.

**Results**: Twenty-one RCTs, five of which were newly identified, were included. Evidence across studies was heterogeneous. Some combinations improved outcomes compared with monotherapy or placebo; however, most combinations lacked sufficient data for meta-analysis. One meta-analysis of amitriptyline combined with lidocaine showed no significant advantage over amitriptyline monotherapy. Adverse event profiles were similar between combination treatment and monotherapy. Several trials did not compare combination treatment with all monotherapy components.

**Discussion/Conclusions**: Despite continued investigation and widespread clinical use, robust evidence supporting the superiority of combination pharmacotherapy is lacking. Combination treatment may remain a reasonable option in cases of inadequate response to monotherapy but should be implemented cautiously with individualized assessments of safety and benefit. Further research is needed to identify effective and safe combination treatments in comparison with all monotherapy components.

## Who’s Checking the Blindfold? A Review of Blinding Assessment Practices in Pharmacotherapy & Neuromodulation RCTs for Neuropathic Pain in Adults

Landon Montag^1^, Nadia Soliman^2^, Xavier Moisset^3^, Michael Ferraro^4^, Daniel Ciampi de Andrade^5^, Ralf Baron^6^, Joletta Belton^7^, David Bennett^8^, Margarita Calvo^9^, Patrick Dougherty^10^, Aki Hietaharju^11^, Koichi Hosomi^12^, Peter Kamerman^13^, Harriet Kemp^2^, Elena Enax-Krumova^14^, Ewan McNicol^15^, Theodore Price^16^, Srinivasa Raja^17^, Andrew Rice^18^, Blair Smith^19^, Fiona Talkington^2^°, Andrea Truini^21^, Jan Vollert^22^, Nadine Attal^23^, Nanna Finnerup^24^, Simon Haroutounian^25^, Luana Colloca^26^, Lene Vase^27^, Tim Salomons^28^, Ramla Abuukar Abdullahi^29^, Matthew Evans^3^°, Sascha Freigang^31^, Bethany Gwyther^32^, David Hohenschurz-Schmidt^33^, Gabriel Taricani Kubota^34^, Jules Phalip^35^, Harrison Phillips^2^, Tjokorda Istri Pramitasuri^36^, Cristina Ramirez-Piriz^37^, Augustus Rottenberg^38^, Nina Taule-Lim^39^, Quyen Than^39^, Jan Wandrey^4^°, Claire Wang^41^, Andreas Zachariadis^2^, Md Zunaid^42^, Ian Gilron^43^

^1^School of Medicine, Queen’s University, Kingston, Canada, ^2^Pain Research Group, Department of Surgery and Cancer, Imperial College London, London, UK, ^3^Université Clermont Auvergne, CHU Clermont-Ferrand, Inserm, Neuro-Dol, Clermont-Ferrand, France, ^4^Centre for Pain IMPACT, Neuroscience Research Australia, Australia; School of Health Sciences, Faculty of Medicine and Health, University of New South Wales Sydney, NSW, Australia), ^5^Center for Neuroplasticity and Pain, Health Science and Technology Department, Faculty of Medicine, Aalborg University, Denmark, ^6^Division of Neurological Pain Research and Therapy, Department of Neurology, Christian-Albrechts-University, Kiel, Germany, ^7^Fraser, CO, USA, ^8^The Nuffield Department of Clinical Neuroscience, University of Oxford, Oxford, UK, ^9^Faculty of Biological Sciences, Pontificia Universidad Católica de Chile, Santiago, Chile; Anesthesiology Division, Faculty of Medicine, Pontificia Universidad Católica de Chile, Santiago, Chile, ^10^Department of Pain Medicine, MD Anderson Cancer Center, Houston, TX, USA, ^11^Department of Neurology, Tampere University Hospital, Tampere, Finland, ^12^Department of Neurosurgery, Osaka University Graduate School of Medicine, Suita, Japan, ^13^Brain Function Research Group, Department of Physiology, School of Biomedical Sciences, Faculty of Health Sciences, University of the Witwatersrand, South Africa, ^14^Department of Neurology, BG University Hospital Bergmannsheil, Ruhr University Bochum, Germany, ^15^Department of Pharmacy Practice, Massachusetts College of Pharmacy and Health Sciences, Boston, MA, USA, ^16^Center for Advanced Pain Studies, Richardson, TX, USA; Department of Neuroscience, University of Texas at Dallas, School of Behavioral and Brain Sciences, Richardson, TX, USA, ^17^Departments of Anesthesiology and Critical Care Medicine and Neurology, Johns Hopkins University School of Medicine, Baltimore, MD, USA, ^18^Pain Research Group, Department of Surgery and Cancer, Imperial College London, London, UK., ^19^Division of Population Health and Genomics, School of Medicine, University of Dundee, Dundee, UK, ^2^°Reading, UK, ^21^Department of Human Neuroscience, Sapienza University, Rome, Italy, ^22^Department of Clinical and Biomedical Sciences, Faculty of Health and Life Sciences, University of Exeter, Exeter, UK, ^23^Inserm U987, APHP, UVSQ Paris Saclay University, Hôpital Ambroise Paré, Boulogne-Billancourt, France, ^24^Danish Pain Research Center, Department of Clinical Medicine, Aarhus University, Aarhus, Denmark, ^25^Department of Anesthesiology, Washington University in St Louis School of Medicine, St Louis, MO, USA, ^26^Department of Pain and Translational Symptom Science, School of Nursing and Department of Anesthesiology School of Medicine, University of Maryland, Maryland, USA, ^27^Department of Psychology and Behavioural Sciences, Aarhus University, Aarhus, Denmark, ^28^Centre for Neuroscience Studies, Queen’s University, Kingston, Canada; Department of Psychology, Queen’s University, Kingston, Canada, ^29^Headache Research, Wolfson CARD, Institute of Psychiatry, Psychology & Neuroscience, King’s College London, UK; Headache Centre, Guy’s and St Thomas NHS Trust, London, UK, ^3^°Division of Anaesthetics Pain Medicine & Intensive Care, Imperial College London, London, UK, ^31^Department of Neurosurgery, Medical University Graz, Graz, Austria, ^32^University of St Andrews, St Andrews, UK, ^33^Pain Research, Department of Surgery and Cancer, Faculty of Medicine, Imperial College, Chelsea, London, United Kingdom, ^34^Department of Neurology, Centre for Neurorehabilitation, Valens, Switzerland; Pain Center, University of Sao Paulo Clinics Hospital, Sao Paulo, Brazil; Center for Pain Treatment, Institute of Cancer of the State of Sao Paulo, University of Sao Paulo Clinics Hospital, Sao Paulo, Brazil, ^35^Faculté de médecine, Fondation ANALGESIA, Clermont-Ferrand, France; CHU Clermont-Ferrand, Inserm 1107 Neuro-Dol, Service de pharmacologie médicale, Université Clermont Auvergne, Clermont-Ferrand, France, ^36^Faculty of Medicine, Udayana University, Denpasar, Indonesia, ^37^King’s College, London, UK, ^38^Bristol Medical School, Bristol, UK, ^39^Siriraj Hospital, Bangkok, Thailand, ^4^°Department of Anaesthesiology and Intensive Care, Campus Charité Mitte and Campus Virchow-Klinikum, Berlin, Germany; Berlin Institute of Health, Charité-Universitätsmedizin Berlin, BIH Biomedical Innovation Academy, BIH Charité Digital Clinician Scientist Program, Berlin, Germany, ^41^Medical Cannabis Research Group, Department of Surgery and Cancer, Imperial College London, London, UK, ^42^Assistant Professor, Bangladesh Civil Service (Health Cadre), Ministry of Health and Family Welfare, Bangladesh, ^43^Department of Anesthesiology and Perioperative Medicine, Queen’s University, Kingston, ON, Canada; Biomedical & Molecular Sciences, Queen’s University, Kingston, ON, Canada; Centre for Neuroscience Studies, Queen’s University, Kingston, ON, Canada; School of Policy Studies, Queen’s University, Kingston, ON, Canada; Kingston Health Sciences Centre, Providence Care Hospital, Kingston, ON, Canada

**Introduction**: In randomized controlled trials (RCTs), study participants and research personnel are often blinded to minimize bias related to knowing treatment allocation. To determine if blinding was effective, participants and research personnel may be asked at the end of the study which treatment they believe they received (“treatment guess”).

**Methods**: We conducted a descriptive review to characterize blinding assessment (BA) and its reporting in pharmacotherapy and neuromodulation neuropathic pain RCTs.

**Results**: Of 290 studies, 37 (13%) reported a BA. Of these studies, 19 were crossover, 17 parallel, and 1 partial crossover in design. Most were single site studies (57%) and received non-industry funding (68%). 27% included an ‘unsure’ answer option for participants’ treatment guess, and 38% asked the reason for the treatment guess. There were no clear trends in BA reporting across time nor based on treatment type. 15/37 studies provided sufficient data to calculate Bang’s Blinding Index (BI) to determine blinding success. Blinding was effect

tive (BI=0 ±0.2) in 10/15 placebo and 10/15 treatment arms, suggesting that participants guessed no better than chance. 6 placebo and 5 treatment arms trended towards unblinding (BI>0.2), whereas 1 placebo and 2 treatment arms trended towards misinformed guessing (BI<-0.2).

**Discussion/Conclusions**: Overall, our findings suggest that BA is done in a minority of neuropathic pain trials, and with variable methodology. Given the importance of minimizing risk of bias due to treatment unblinding, further research and consensus building is necessary to determine if and/or how BA should be conducted and interpreted in analgesic clinical trials.

## Effectiveness and tolerability of pharmacological prophylaxis for chronic migraine: a systematic review and network meta-analysis of randomized controlled trials

Malahat Khalili^1^, Faraidoon Haghdoost^2^, Amin Liaghatdar^3^, Kian Torabiardakani^3^, Fatemeh Mahdian^3^, Tal Levit^3^, Sara Moradi^3^, Ehsan Hedayati^4^, Farzaneh Ahmadi^3^, Sahar Khademioore^3^, Tariq Atkin-Jones^3^, Vivek Patil^3^, Fatemeh Mirzayeh Fashami^3^, Kameshwar Prasad^5^, Seyed-Mohammad Fereshtehnejad^6^, Jason W. Busse^7^, Behnam Sadeghirad^7^

^1^Michael G. DeGroote Institute for Pain Research and Care, McMaster University, Hamilton, ON, Canada, ^2^The George Institute for Global Health, University of New South Wales, Sydney, Australia, ^3^McMaster University, Hamilton, ON, Canada, ^4^École d’optométrie, Université de Montréal, Montréal, QC, Canada, ^5^Department of Neurology, Fortis Hospital, Sector B1, Vasant Kunj, New Delhi, ^6^Division of Neurology, University of Toronto, Toronto, Ontario, Canada, ^7^Department of Anesthesia, McMaster University, Hamilton, ON, Canada

**Introduction**: Migraine headaches are common and potentially disabling disorder, with several interventions available for prevention and symptom reduction. We conducted a network meta-analysis of randomized controlled trials to compare the effectiveness and tolerability of prophylactic pharmacological interventions for chronic migraine.

**Method**: We searched MEDLINE, Embase, Cochrane Central, PsycINFO, Web of Science, and Scopus from their inception to July 2025. We included randomized controlled trials evaluating prophylactic pharmacological interventions in adults with chronic migraine. Pairs of reviewers independently extracted data, and the certainty of evidence was assessed using the GRADE approach.

**Results**: We included 40 RCTs (13 163 participants). Compared to placebo, valproate [MD -10.36 (95% CI: -15.42 to -5.30), low certainty] and botulinum toxin [MD -2.48 (95% CI: -4.40 to -0.56), very low certainty] were the most effective in reducing monthly migraine/headache days. The evidence is very uncertain about the effect of botulinum toxin [MD -1.43 (95% CI: -2.59 to -0.27), very low certainty] in reducing migraine/headache attacks. The moderate evidence suggests fremanezumab (relative risk [RR] 2.02, 95%CI 1.30 to 3.15) and Erenumab (RR 1.58, 95%CI 1.01 to 2.46) probably improve 50% response rate. However, very low certainty evidence found that patients were more likely to discontinue topiramate (RR 1.42, 95%CI 1.05 to 1.90) due to adverse events compared to placebo.

**Discussion/Conclusions**: No intervention showed superiority across all patient-important outcomes. Evidence suggests certain CGRP antagonists may improve some outcomes, however, these effect estimates were imprecise and mainly driven by trials sponsored by industry. Valproate and botulinum toxin may be associated with improvement in certain outcomes. Most studies had small sample sizes, and many comparisons were based on limited number of trials, which may have impacted certainty of evidence, statistical power, and led to imprecise estimates.

## Exploring Cognitive Alterations Associated with Opioid-Induced Sedation in Acute Pain Care: A Scoping Review

Danielle Dunwoody^1^, Dinesh Nethirasigamani^2^, Amanda Ross-White^3^, Callie Loveday^1^, Meredith Kuipers^1^, Vidhi Patel^4^, Monakshi Sawhney^4^

^1^Brock University, Faculty of Applied Health Sciences, Department of Nursing, ^2^Halton Healthcare, Department of Anaesthesiology, ^3^Queen’s University, Bracken Health Science Library, ^4^Queen’s University, Faculty of Health Sciences, School of Nursing

**Introduction**: Over 50% of hospitalized patients receive opioids for pain. Current nursing assessment tools lack the sensitivity and specificity required to detect adverse events early.

Dunwoody et al. (2019) suggest a potential link between changes in cognition, beyond arousability, when identifying opioid induced sedation. A scoping review was conducted regarding the relationship between opioid-induced sedation and cognitive changes within acute pain management.

**Methods**: A comprehensive literature search, conducted with a research librarian, examined CINAHL, Embase, Medline, and CENTRAL for peer-reviewed studies published between 1946 to present. Studies were included if they involved adult post-operative patients who received opioids as part of their post-operative pain management, along with having sedation assessments conducted and cognition measured. Citations were collated and uploaded into Covidence©. Following a pilot test, titles and abstracts were screened against the inclusion criteria for the review. Overall, 261 studies were deemed eligible for full-text review and underwent an independent secondary screening process by three authors to evaluate their eligibility against the inclusion criteria.

**Results**: Twelve studies were included in this review, comprised of eleven randomized controlled trials and one case series. Studies originated from China (n= 5), with one each from Canada, United States, Australia, Egypt, France, Sweden, and the United Kingdom.

## Data collection time points varied, with no study designating sedation as a primary outcome. Cognitive change related to opioid administration was a key outcome in seven studies.

**Discussions/Conclusions**: These findings reveal limited evidence regarding opioid-induced sedation with cognitive outcomes in acute pain management, warranting further research.

A review of pain management education in undergraduate nursing programs: current evidence and future directions

Marilyn Tousignant^1^, Martin Charette^1^, Emilie Paul-Savoie^1^

^1^Université de Sherbrooke

**Introduction**: Pain is a complex and pervasive phenomenon in healthcare, and its increasing prevalence makes it a major quality-of-care issue. Nurses play a central role in pain assessment and management, requiring strong knowledge and skills for optimal care. Yet evidence shows that nursing graduates display substantial gaps in this area, limiting their professional practice.

**Objectives**: To describe (1) the characteristics of pain education in undergraduate nursing programs, and (2) students’ knowledge levels and the assessment methods used.

**Methods**: A rigorous and comprehensive literature review was conducted using the PCC framework (Population: nurses; Concept: university education; Context: pain). The databases CINAHL, MEDLINE, ERIC, AMED and Education Source were searched. Of the 387 identified articles, 32 met the inclusion criteria. Data were extracted and synthesized descriptively, following principles of transparency and reproducibility inspired by systematic review methodology.

**Results**: Studies revealed limited and inconsistent instructional hours, heterogeneous teaching structures, and a predominance of lecture-based formats. Content mainly addressed neurophysiology, assessment, pharmacological and non-pharmacological interventions, while interprofessional collaboration was underrepresented. Students demonstrated limited knowledge and occasionally negative attitudes toward pain management, influenced by both the quality and quantity of their training.

**Discussion**: These findings highlight persistent gaps in undergraduate pain education, both in scope and in pedagogical approach. The reliance on lectures and insufficient emphasis on experiential learning may hinder the development of clinical judgment and reflective competencies.

**Conclusion**: Strengthening pain education across undergraduate and continuing nursing programs is essential to support effective practice and improve quality of care.

## Pharmacologic treatments for fibromyalgia: A systematic review and network meta-analysis of randomized trials

Haotao Li^1^, Dena Zeraatkar^1,2^, João Pedro Lima^2^, Sarah Kirsh^2^, Michael Ling^1^, Hamed Movahed^2^, Alex Huynh^1^, Tanvir Jassal^1^, Alicia Walch^1^, Mahsa Raji Lahiji^2^, Tyler Pitre^3^, Rachel Couba^1^, Behnam Sadeghirad^1,2^, Thomas Agoritsas^2,4^, Jason Walter Busse^1,2^

^1^Department of Anesthesia, McMaster University, Hamilton, ON, Canada, ^2^Department of Health Research Methods, Evidence, and Impact, McMaster University, Hamilton, ON, Canada, ^3^Division of Respirology, Department of Medicine, University Health Network, Toronto, ON, Canada, ^4^Department of Rheumatology, Geneva University Hospitals, Geneva, Switzerland; The MAGIC Evidence Ecosystem Foundation, Oslo, Norway

**Background**: Fibromyalgia is a chronic disorder characterized by widespread musculoskeletal pain, physical impairments, and cognitive difficulties.

**Objective**: To compare the effectiveness of pharmacologic treatments for fibromyalgia.

**Methods**: We searched MEDLINE, EMBASE, and Cochrane CENTRAL from inception to January 2025 for randomized trials comparing pharmacologic agents with placebo or usual care in adults with fibromyalgia. Two reviewers independently screened studies, extracted data, and assessed risk of bias. We performed a frequentist random-effects network meta-analysis. Outcomes included global impression of recovery or improvement, pain intensity, physical and mental health, fibromyalgia symptoms, sleep quality, return to work or education, treatment discontinuation, and serious adverse events. The GRADE guided our assessment of evidence certainty.

**Results**: 151 trials (32,592 participants) proved eligible. Compared with placebo or usual care, low-certainty evidence suggested that sodium oxybate, selective norepinephrine reuptake inhibitors, serotonin-norepinephrine reuptake inhibitors, and gabapentinoids may increase the likelihood of meaningful improvement (105-283 more per 1,000 patients). Low-certainty evidence also indicated that sodium oxybate (-8.81; 95% CI -11.78 to -5.84) and selective serotonin reuptake inhibitors (-8.02; 95% CI -13.40 to -2.64) may reduce fibromyalgia symptoms, while acetaminophen-tramadol combinations may improve physical health (5.0; 95% CI 2.62 to 7.38). No evidence showed benefits for pain intensity, mental health, or increased risks of discontinuation or serious adverse events.

**Discussion/Conclusions**: Existing trials have major limitations (notably high attrition) and U.S. Food and Drug Administration-approved drugs yield only modest benefits, with limited improvements in select outcomes. Current guidance should continue to prioritize non-pharmacologic approaches.

**Registration**: PROSPERO CRD42012003291.

## Pain Modulation Mechanisms in Endurance Athletes: A Scoping Review to Inspire Chronic Pain Management

Emilie Paul-Savoie^1^, Véronique Boudreault^2^, Maëlli Fernandez-Gendron^1^, Josiane Provost^1^, Emilie Lagueux^3^, Joannie Desroches^4^, Antoine Groleau^1^, Colin Rivard^1^, Catherine Dumoulin^1^, Emilie Gosselin^1^

^1^École des sciences infirmières, Faculté de médecine et des sciences de la santé, Université de Sherbrooke, ^2^Faculté des sciences de l’activité physique, Université de Sherbrooke, ^3^École de réadaptation, Faculté de médecine et des sciences de la santé, Université de Sherbrooke, ^4^Patiente partenaire

**Introduction**: Chronic pain is a major public health issue, often associated with impaired endogenous pain modulation. In contrast, endurance athletes deliberately expose themselves to painful situations, and their ability to perform despite pain is central to their success. Evidence suggests they exhibit greater pain tolerance than sedentary individuals. However, the underlying explanatory mechanisms remain unclear. Current evidence is fragmented across disciplines and methodologies, underscoring the need for integrative biopsychosocial synthesis. The main objectives are to map existing knowledge on the biological, psychological and social mechanisms underlying pain modulation and tolerance in endurance athletes, and to explore their potential relevance for chronic pain management.

**Methods**: Following the Joanna Briggs Institute scoping review methodology with a patient partner, a comprehensive search was conducted across 11 databases and grey literature. Using the PCC framework, two independent reviewers screened and selected quantitative, qualitative, and mixed-method studies. Synthesis focused on methodology, study context, and mechanistic dimensions.

**Preliminary Results**: Most studies were quantitative, employing diverse methodologies across endurance disciplines. Athletes consistently showed greater pain tolerance, potentially explained by more efficient descending inhibitory control, exercise-induced hypoalgesia and adaptive cognitive-affective mechanisms such as lower fear of pain, reduced catastrophizing and greater acceptance and resilience. Social influences emphasizing persistence and strategic focus also emerged as modulatory factors.

**Discussion/Conclusions**: Endurance athletes demonstrate distinct, dynamic pain modulation mechanisms integrating biological, psychological, and social components. Despite converging evidence, direct biological measures remain limited, warranting integrated experimental approaches. A forthcoming stakeholder consultation will validate these findings and prioritize their application to chronic pain management.

## Epidural Blood Patch for Post-Dural Puncture Headache: A Systematic Review and Meta-Analysis of Randomized Controlled Trials

Armaanpreet Dhillon^1^, Jackie Han^2^, Brandon Lee^2^, Amber Ansh^2^, Daniel Cordovani^3^, Juan Segura Salguero^3^, Li Wang^3^

^1^Western University, ^2^McMaster University, ^3^Department of Anesthesia, McMaster University

**Introduction**: Post-dural puncture headache (PDPH) is a common complication of lumbar puncture and neuraxial anesthesia. Epidural blood patch (EBP) is the standard treatment, though its effectiveness varies. We aimed to systematically assess the efficacy and safety of EBP in the treatment of PDPH.

**Methods**: We searched CENTRAL, CINAHL, CLIB, EMBASE, MEDLINE, PsycINFO, PubMed, and SCI up to October 2024 for RCTs comparing EBP to sham, conservative or other active treatments for 95%CI for pain and function after converting into the 10cm VAS. We assessed the certainty of evidence using GRADE approach.

**Results**: Ten studies with 533 participants were included. Low certainty evidence suggests that EBP may reduce PDPH intensity (WMD -4.72cm, 95%CI [-7.71, -1.73]) at 24 hours; but may increase the risk of back pain (RR 4.89, 95%CI [1.56-15.26]). Low certainty evidence indicates that epidural fibrin patches may provide more pain reduction than EBP (MD 1.03cm [0.39-1.67] at 24 hour; 0.73cm [0.15-1.31] at 30 days). Low to very low certainty evidence shows that, compared to IV Cosyntropin, EBP might reduce pain and function at 24 and 72 hours (Pain: MD -4.00cm [-4.93, -3.07] and -1.00cm [-1.93, -0.07]; Function: MD -5.00 [-6.11, -3.89] and -1.30 [-2.41, -0.19]). No significant differences in pain relief were found between EBP doses of 7.5, 15, 20 and 30 mL.

**Discussion/Conclusions**: EBP may reduce PDPH-related pain intensity compared to conservative treatment and IV Cosyntropin; but may also increase the risk of back pain. Given the low to very low certainty of evidence, further high-quality trials are needed to establish the optimal management strategy for PDPH.

## Reporting Quality of Randomized Controlled Trials of Pharmaceutical Interventions for Pediatric Chronic Pain: Comparison of CONSORT 2010 and 2025 guidelines

Parsia Parnian^1^, Lucas Lorimer^2^, Erinne Tian^3^, Bryan Xia^3^, Carolyn Lam^3^, Malcolm Serran^1^, Joy Chowdhury^1^, Aman Hemani^4^, Li Wang^5^

^1^University of Toronto, ^2^Royal College of Surgeons in Ireland, ^3^McMaster University, ^4^Queen’s University, ^5^Department of Anesthesia, McMaster University

**Introduction**: Chronic pain affects 11 to 38% of children, significantly affecting physical, psychological, and socio-emotional well-being. Despite its prevalence, pediatric chronic pain remains underserved, largely due to limited high-quality evidence. We systematically assessed the adherence to CONSORT 2010 and 2025 reporting guidelines in randomized controlled trials (RCTs) of pharmaceutical interventions for pediatric chronic pain.

**Methods**: We searched CENTRAL, CINAHL, Embase, MEDLINE, and Web of Science for RCTs evaluating pharmaceutical interventions for pediatric chronic pain. Two reviewers independently assessed adherence to each CONSORT checklist item (37 items for 2010; 40 for 2025). Each item was scored as 1 (reported) or 0 (not reported), with multi-component items subdivided and averaged to yield fractional scores.

**Results**: Ninety-one RCTs with 8,333 pediatric patients proved eligible. The overall adherence rate to CONSORT 2010 was 55.8% (95% CI 54.1-57.5%) with an average score of 22.7/37 points (95% CI 21.5-23.9); and the adherence rate to CONSORT 2025 was 46.1% (95% CI 44.4-47.8%) with an average score of 20.9/40 points (95% CI 19.7-22.0). Inadequate reporting (<20% of trials) was common across both guidelines for protocol changes, trial design (type, randomization, allocation ratio, framework), intervention details, interim analyses, trial termination reasons, and effect size estimates. New transparency items in CONSORT 2025, such as data sharing and patient involvement, were also underreported.

**Discussion/Conclusions**: Reporting quality in RCTs of pharmaceutical interventions for pediatric chronic pain is suboptimal, particularly under the updated CONSORT 2025 guideline. Journals and researchers should endorse CONSORT to promote transparent trial reporting in pediatric field.

## Effectiveness and tolerability of perioperative interventions to prevent chronic pain after knee or hip replacement surgery: A systematic review with network and component network meta-analysis of randomized controlled trials

Azin Khosravirad^1^, Malahat Khalili-Kisomi^1^, Ian Gilron^2^, James Khan^3^, Luis E. Chaparro^4^, Andrea J. Darzi^5^, Lawrence Mbuagbaw^1^, Jason W. Busse^5^, Behnam Sadeghirad^5^

^1^Department of Health Research Methods, Evidence, and Impact (HEI), McMaster University, Hamilton, Ontario, Canada, ^2^Departments of Anesthesiology and Perioperative Medicine, Queen’s University, Kingston, Canada, ^3^Department of Anesthesiology and Pain Medicine, University of Toronto, Toronto, Ontario, Canada, ^4^Department of Anesthesia, Grand River Hospital, Kitchener, ON, Canada, ^5^Department of Anesthesia, McMaster University, Hamilton, Ontario, Canada

**Introduction**: Chronic pain is a common and impactful complication after musculoskeletal and orthopedic surgeries. We conducted a systematic review with network and component network meta-analysis of randomized trials to evaluate the effectiveness and tolerability of perioperative pharmacological interventions aimed at preventing chronic pain after knee or hip replacement surgery.

**Methods**: We searched MEDLINE, Embase, PsycInfo, CINAHL, and the Cochrane CENTRAL until February 2025. Eligible trials, (1) enrolled adult patients undergoing knee or hip replacement surgeries, (2) randomized patients to any pharmacotherapy, their combination, or placebo aimed at reducing pain after surgery, and (3) assessed pain at ≥3 months after surgery. Our outcomes of interest included the proportion of patients reporting chronic post-surgical pain (CPSP), pain severity, physical and emotional functioning, and drop out rates.

**Results**: We included 59 randomized trials (7,705 patients). At 3 to 6 months after knee/hip replacement, epidural corticosteroid injection reduced the proportion of patients with CPSP compared to usual care (risk ratio (RR) 0.35, 95% CI [0.14, 0.90]; risk difference (RD), 11.7% fewer patients). At 6 to 12 months, wound infiltration of corticosteroids reduced pain intensity compared to usual care (MD -0.31, 95% CI [-0.57, -0.05]). At the longest follow-up time, ketamine improved physical function compared to usual care (MD 10.77, 95% CI [5.2, 16.3]), while nefopam and wound infiltration of corticosteroids also showed modest improvements. Component network meta-analysis revealed that the corticosteroid component was associated with reduced CPSP risk (incremental RR 0.58, 95% CI [0.39, 0.86]). Benefits were primarily observed in knee replacement surgeries, with no interventions showing statistically significant benefits in hip replacement surgeries patients.

**Discussion/Conclusions**: Epidural corticosteroids may be associated with small benefit for reducing CPSP, while wound infiltration of corticosteroids and ketamine may result in small but unimportant reduction in pain intensity and improvement of physical functioning.

## Patient education and self-management in adults with temporomandibular disorders: Results from a systematic review with meta-analysis

Geneviève Ferland^1,2^, Raphaël Vincent^1,2^, François Desmeules^1,2^, Audrey-Anne Cormier^1,2^, Moira Huon^3^, Laurent Pitance^3^

^1^École de la réadaptation, Faculté de Médecine, Université de Montréal, ^2^Centre de recherche de l’Hôpital Maisonneuve-Rosemont, ^3^Université Catholique de Louvain

**Aim**: To evaluate the effectiveness of patient education (ED) and self-management (SM) interventions compared to other non-surgical interventions, such as occlusal splints, manual therapy, electrotherapy or multimodal approach, on pain outcomes for adults with temporomandibular disorders (TMDs).

**Methods**: An electronic search was conducted up to March 2025, using terms related to ED, SM, and TMDs. Methodological quality was assessed with the Cochrane Risk of Bias tool V1. Random-effects meta-analyses were performed and pooled standardized mean differences (SMD) were calculated. Certainty of evidence was rated with the GRADE approach.

**Results**: Forty-seven RCTs were included (n=3238 participants). Based on very low-certainty evidence, other non-surgical interventions (supervised exercise, manual therapy, or splints) may be more effective than ED and SM alone for short-term pain reduction (SMD= 0.67, 95%CI: 0.13 - 1.20, 6 studies, 323 patients). Other comparisons, such as ED and SM alone versus ED and SM combined with other non-surgical interventions, showed comparable effects between groups on short-, medium- and long-term pain reductions. The certainty of evidence supporting these findings remains low to very low.

**Discussion/Conclusions**: While some short-term benefits may favor other non-surgical treatments, combining ED and SM with other interventions did not enhance outcomes. More high-quality trials are needed to determine the effectiveness of ED and SM and optimal delivery of ED and SM in TMD care.

## Examining Organizational Processes and Clinical Practices Implemented for Chronic Pain Management in Primary Care in the Context of the 2021-2026 Quebec Chronic Pain Action Plan (Canada)

Gabriella Lavoie Dias^1,2^, Tania Augière^1,2^, Yves Couturier^3,4^, Nick-Kevin Jérôme^1,2^, Manon Choinière^1,5^, M. Gabrielle Pagé^1,2,5^

^1^Centre de recherche du Centre hospitalier de l’Université de Montréal (CRCHUM), ^2^Département de psychologie, Université de Montréal, ^3^Université de Sherbrooke, ^4^Centre de recherche du Centre hospitalier de l’Université de Sherbrooke (CRCHUS), ^5^Département d’anesthésiologie et médecine de la douleur, Université de Montréal

**Introduction**: Chronic pain is a complex and persistent condition whose treatment remains challenging, especially in primary care where access to specialized services is limited. The 2021-2026 Quebec Chronic Pain Action Plan aims to improve access to quality care across the healthcare continuum.

**Objective**: This study examined the organizational and clinical implementation of new interprofessional primary care services for chronic pain management by documenting and analyzing decision-making, organizational processes, and clinical practices.

**Methods**: A mixed-methods case study was conducted in five sites (n = 5). Nine semi-structured interviews (n = 9) with managers and a self-administered questionnaire completed within the first six months of service implementation were analyzed. Qualitative and quantitative data were triangulated to identify key facilitators, challenges, and conditions for successful implementation.

**Results**: Teams implemented innovative interprofessional models centered on self-management and individualized care, delivered mostly independently of medical care. Despite limited physician involvement (physician engagement in care planning estimated at 20%) and fragile partnerships (ease of communication with referring physicians estimated at 62%) affecting continuity, care models often relied on coordination of patient-centered plans by nurse practitioners. Access and eligibility criteria varied across sites and over time. Despite financial and organizational constraints, teams showed adaptability, innovation, and strong commitment to accessibility and continuity.

**Discussion/Conclusions**: These models represent an important shift in primary care chronic pain management. By transferring follow-up to interprofessional teams, they support a comprehensive approach aligned with the Action Plan. Although challenges remain regarding medical engagement, partnerships, and resources, the models show promise for strengthening coordination and quality of care in Quebec’s primary care network.

## The benefits and harms of physical activity for patients with myalgic encephalomyelitis/chronic fatigue syndrome: a systematic review and meta-analysis of randomized trials

Sarah Kirsh^1^, Oswin Chang^2^, Michael Ling^1^, Tanvir Jassal^1^, João Pedro Lima^1^, Mahsa Raji Lahiji^1^, Rachel Couban^1^, Dena Zeraatkar^1^, Jason W. Busse^1^

^1^McMaster University, ^2^University of British Columbia

**Introduction**: Myalgic encephalomyelitis/chronic fatigue syndrome (ME/CFS) is a condition characterized by persistent fatigue, post-exertional malaise, cognitive dysfunction, and unrestful sleep. For decades, guidelines recommended graded physical activity (GPA) to improve patients’ physical capacity, though concerns about post-exertional malaise, a defining characteristic of ME/CFS, have led many to question its safety and effectiveness.

**Objective**: To systematically review and summarize evidence from randomized trials on the benefits and harms of physical activity for adults with ME/CFS.

**Methods**: We searched MEDLINE, EMBASE, PsycInfo, and CENTRAL from inception to July 2024 for randomized controlled trials comparing physical activity with usual care, pacing, or cognitive and behavioral interventions in adults diagnosed with ME/CFS. Reviewers worked independently and in duplicate to screen records, extract data, and assess risk of bias. We performed random-effects meta-analyses and rated the certainty of evidence using the GRADE approach.

**Results**: Nineteen trials (37 records; n=2,684) proved eligible. Moderate certainty evidence suggests that GPA probably reduces fatigue compared with control (mean difference [MD] -6.14, 95% CI -9.95 to -2.33) and probably decreases post-exertional malaise (risk difference [RD] -17.2%, 95% CI -25.8% to -7.9%). The addition of cognitive behavioral therapy to GPA provided little or no additional benefit for fatigue (MD -0.60, 95% CI -2.21 to 1.01). Moderate certainty evidence suggests that traditional Eastern movement therapies probably reduce fatigue (MD -2.85; 95% CI -4.07 to -1.64).

**Discussion/Conclusions**: Graded exercise and Eastern movement therapies likely improve fatigue in adults with ME/CFS. These findings will inform forthcoming guideline recommendations on the management of ME/CFS.

## Harms Associated with Inhaled Cannabis for Management of Chronic Pain and Other Symptoms: A Systematic Review and Meta-Analysis of Observational Studies

Wenjun Jiang^1^, Jane Jomy^2^, Abigail Chu^3^, Mona Elmikaty^4^, Rachel Couban^5^, Li Wang^5,6^, Jason Busse^5,6,7,8^

^1^Michael G. DeGroote School of Medicine, McMaster University, ^2^Temerty Faculty of Medicine, University of Toronto, ^3^Faculty of Health, University of Waterloo, ^4^Faculty of Science, McMaster University, ^5^Michael G. DeGroote Institute for Pain Research and Care, McMaster University, ^6^Department of Anesthesia, McMaster University, ^7^Department of Health Research Methods, Evidence, and Impact (HEI), McMaster University, ^8^Michael G. DeGroote Centre for Medicinal Cannabis Research, Hamilton

**Introduction**: Inhaled cannabis products are increasingly used to manage chronic pain and other symptoms; however, the harms associated with use remain uncertain. We conducted a systematic review to inform harms associated with inhaled cannabis.

**Methods**: We searched MEDLINE, EMBASE, PsychInfo, and Web of Science for non-randomized studies reporting on harms associated with inhaled cannabis use, from inception to May 30, 2025. We used random-effects models for meta-analyses.

**Results**: We identified 31 observational studies with 62,463 cannabis-using participants that reported 90 adverse events. Our meta-analyses of observational studies found that inhaled cannabis use was associated with anxiety (22% 95%CI [14-31%]), depression (9% [5-15%]), stress (8% [2-18%]), paranoia (12% [5-21%]), dizziness (15% [9-23%]), amnesia (25% [15-36%]), impaired attention (8% [5-12%]), confusion (7% [4-11%]), hallucinations (7% [4-12%]), impaired balance or coordination (6% [3-9%]), fatigue (31% [22-42%]), cough (26% [6-53%]), dry mouth (45% [31-59%]), and increased appetite (26% [13-42%]). Compared to non-cannabis users, inhaled cannabis use was associated with increased risk of: wheezing (OR 2.34, 95%CI [1.77-3.09]), cough (OR 1.81 [1.39-2.36]), and depression (OR 1.92 [1.17-3.17]); association with shortness of breath (OR 1.21 [0.78-1.87]) and lung cancer (OR 1.45 [0.89-2.39]) were not significant.

**Discussion/Conclusions**: Our review found inhaled cannabis users frequently experienced anxiety, amnesia, fatigue, coughing, dry mouth, and increased appetite. Compared to non-users, individuals using inhaled cannabis products are twice as likely to report wheezing and depression. Our findings will support shared decision-making for patients considering inhaled cannabis products for therapeutic purposes.

## A Descriptive Study of the Implementation of ECHO Chronic Pain in Canada: A Qualitative Thematic Analysis

Da Beattie^1^, Lori Montgomery^2,3^, Helena Daudt^4^, Jean-François Leroux^5^, Q. Jane Zhao^1,6^, Yalnee Shantharam^6^, Behdin Nowrouzi-Kia^1^, Sanjeev Sockalingam^1,6,7^, Andrea D. Furlan^1,6^

^1^University of Toronto, ^2^Primary Care Alberta, ^3^Cumming School of Medicine, ^4^Pain BC, ^5^Health Canada, ^6^University Health Network, ^7^Centre for Addiction and Mental Health

**Introduction**: Extension for Community Healthcare Outcomes (ECHO) is a tele-educational program used to disseminate knowledge through an all-teach, all-learn approach. ECHO was introduced in Canada in 2014 for pain. At its peak, 12 ECHO Pain hubs existed across 5 provinces. The Diffusion of Innovations (DofI) by Everett Rogers is a theory that explains how, why, and the rate at which new innovations spread. We aim to describe the implementation of ECHO Chronic Pain in Canada to improve the future implementation of ECHO across disciplines.

**Methods**: Qualitative semi-structured key-informant interviews were conducted with ECHO Pain staff members and funders. Consent was gathered via email. Interviews were conducted on Zoom and Microsoft Teams, transcribed, analyzed using NVivo 15, and interpreted using the DofI and thematic analysis.

**Results**: Fifteen interviews were conducted. As per the DofI, a family physician (change agent) discovered ECHO in the United States and partnered with a physiatrist (opinion leader) to start the first ECHO (innovator). Early and late adopters stated a gap in primary care physicians’ knowledge on pain, long wait times for specialists, and positive results from existing hubs as factors that affected their decision to adopt ECHO. Early adopters said mentorship from the mother hub in New Mexico was critical for implementation and late adopters similarly suggested the importance of mentorship from early adopters.

Discussion/Conclusions: We emphasize the importance of opinion leaders leveraging their networks to accelerate the diffusion of ECHO as well as the integral relationship between change agents and opinion leaders.

## Evaluating the Implementation of Infant Pain Practice Change (ImPaC) Resource: Are Improvements to Pain Outcomes Sustained Over Time?

Bonnie Stevens^1^, Shirine Riahi^1^, Mariana Bueno^2^, Melanie Barwick^1^, Marsha Campbell-Yeo^3^, Christine Chambers^3^, Carole Estabrooks^4^, Rachel Flynn^5^, Sharyn Gibbins^6^, Denise Harrison^7^, Wanrudee Isaranuwatchai^8^, Sylvie LeMay^9^, Melanie Noel^10^, Jennifer Stinson^1^, Anne Synnes^11^, Charles Victor^2^, Janet Yamada^12^

^1^The Hospital for Sick Children, ^2^University of Toronto, ^3^Dalhousie University, ^4^University of Alberta, ^5^University College Cork, ^6^Trillium Health Partners, ^7^University of Melbourne, ^8^Health Intervention and Technology Assessment Program (HITAP) Foundation (Thailand), ^9^Université de Montréal, ^10^University of Calgary, ^11^University of British Columbia, ^12^Toronto Metropolitan University

**Introduction**: The interactive, evidence-based ImPaC Resource website guides healthcare teams in improving infant pain practices in neonatal intensive care units (NICUs). After establishing the clinical effectiveness of ImPaC, we determined whether improvements in outcomes were sustained over time beyond the 6-month intervention period.

**Methods**: In a waitlist randomized controlled trial, 23 Canadian NICUs were randomized to either the ImPaC intervention (INT) or Usual Care (UC). Pain metrics were collected from 30 infant charts/NICU at baseline, 6-, 12- and 18-months post ImPaC (INT); and at baseline, end of the 6-month waitlist, 6- and 12-months post ImPaC (UC). Data were analyzed descriptively and compared over time using generalized estimating equations models to account for within unit correlation.

**Results**: Over time there was a significant reduction in the frequency of painful procedures/infant/day, from 3.34 at baseline to 2.75 by 18-months (p=0.023). There was a significant increase over time in the proportion of painful procedures associated with pain assessment, from 28% at baseline to 39.1% by 18-months (p < 0.001). Although there was a slight increase over time in the proportion of painful procedures accompanied by non-pharmacological pain management strategies, from 26.15% at baseline to 28.4% at 18-months, the changes were not significant (p=0.089).

**Discussion/Conclusions**: Sustainability is characterized by the maintenance of clinical outcomes. However, the continuation of practices, their integration into organizational routines and adaptation to change, and contextual barriers and facilitators make sustainability a complex phenomenon to interpret. Parallel research on these important components is required.

## Gender/sex differences

### Les différences entre les genres et les sexes

## Investigating sex differences in momentary associations between negative mood and assessment time-point with pain intensity in chronic low back pain

Elisabeth Lamoureux^1,2^, Karen Ghoussoub^1,2^, Élise Develay^1^, Sonia Lupien^3^, Pierre Rainville^4,5^, Mathieu Roy^6^, Étienne Vachon-Presseau^7^, M. Gabrielle Pagé^1, 2, 8^

^1^Centre de recherche du Centre Hospitalier de l’Université de Montréal, Montreal, QC, ^2^Department of Psychology, University of Montreal, Montreal, QC, ^3^Department of Psychiatry, University of Montreal, Montreal, QC, ^4^Faculty of Dentistry, University of Montreal, Montreal, QC, ^5^Centre de recherche de l’Institut universitaire de gériatrie de Montréal, Montreal, QC, ^6^Department of Psychology, McGill University, Montreal, QC, ^7^Faculty of Dental Medicine and Oral Health Sciences, McGill University, Montreal, QC, ^8^Department of Anesthesiology, University of Montreal, Montreal, QC

**Introduction**: From changes in neurocircuitry to differences in pain perception and sensitivity, evidence shows that sex and negative affect can significantly impact pain. Yet the dynamic nature of their associations with pain remains to be more thoroughly investigated, especially in the context of chronic pain. This study aimed to evaluate sex differences in the associations between momentary negative mood, assessment time-point and pain (both at the intra- and inter-individual levels) among adults living with chronic low back pain (cLBP).

**Methods**: Participants (N=197, age=48.7±12.4 years, 57.9% woman) were recruited from a province-wide cohort of adults living with cLBP and advertisements in medical-related clinics across Quebec to participate in a prospective longitudinal mixed-methods study. This study is based on quantitative data from daily diaries, which participants were asked to complete 3 times per day for 7 days to track their mood and pain intensity.

**Results**: Multilevel analyses stratified by sex showed that both men and women tended to report significantly higher pain intensity when their mood was lower on average (b=0.43, *p*<.001; and b≈0.55, *p*<.001 respectively) and when it was worse than their usual levels (b≈0.35, *p*<.001, and b≈0.31, *p*<.001 respectively). Interestingly, end-of-day pain scores were higher compared to the morning for both men (b≈0.19, *p*≈.03) and women (b≈0.38, *p*<.001).

**Discussion/Conclusions**: Findings suggests that negative mood modulates momentary pain intensity among individuals with cLBP, regardless of their sex. This underscores the importance of considering mood variations when developing treatment plans for cLBP and supports treatment tailoring based on individual’s average mood.

## Anal Pain During Receptive Intercourse: High Symptom Burden, Modifiable Causes, and Consent Dynamics

Samantha Levang^1^, Bibiana Kemerer^1^, Daniel Dickstein^2^, Wendy Zukerman^3^, Blythe Terrell^3^, Caroline Pukall^1^

^1^Queen’s University, ^2^Icahn School of Medicine at Mount Sinai, ^3^Spotify Studios

**Introduction**: Receptive anal intercourse (RAI) is common, yet anal pain during RAI is rarely quantified with validated measures or examined in relation to consent and sexual experience.

**Methods**: Adults (N = 1,796) completed an online survey on sexual practices for the Science Vs Podcast. Participants reporting partnered RAI answered two PROMIS SexFS Anal Discomfort items; summed scores were converted to T‑scores using the PROMIS summary‑score conversion table. T‑score analyses were restricted to participants reporting partnered RAI in the past 30 days. Regression models tested associations between anal discomfort T‑scores and age, lifetime RAI frequency, gender/sexual‑orientation cohort, pressure to engage in RAI, and anal masturbation history. Descriptive analyses examined pain appraisal (desirable vs bothersome/distressing) and perceived reasons (modifiable vs medical).

**Results**: Anal discomfort T‑scores indicated substantial pain/discomfort, with scores clustered toward the higher end of the PROMIS conversion range. Older age and greater lifetime RAI frequency were associated with lower T‑scores. Cohort differences were significant; cisgender heterosexual men reported lower T‑scores than cisgender women and sexual‑minority cohorts. Pressure to engage showed a strong independent association with higher T‑scores, whereas anal masturbation history was modestly associated with lower T‑scores. Among participants with any anal pain, most described it as bothersome or distressing and attributed it primarily to modifiable factors (e.g., inadequate lubrication); relatively few cited gastrointestinal or inflammatory conditions.

**Discussion/Conclusions**: Anal pain during partnered RAI appears common and substantial, closely tied to pressure to engage and modifiable behaviours, highlighting targets for pain‑informed sexual counseling and education across diverse sexual and gender groups.

## Men show greater motor reactivity and lower pain sensitivity than women

Celina Davila Felipe^1,2,3^, Soichi Kurihara-Piché^1,2,3,4^, Anne-Sophie Marion^1,2,3^, Yuuto Kurihara-Piché^1,2,3^, Takatoshi Satake^1,2,3^, Mathieu Piché^1,2,3^

^1^Département d’Anatomie, Université du Québec à Trois-Rivières, Trois-Rivières, QC, Canada, ^2^Institut National d’Anatomie, Université du Québec à Trois-Rivières, Trois-Rivières, QC, Canada, ^3^Centre de Recherche de l’Institut Universitaire de Gériatrie de Montréal, Montréal, QC, Canada, ^4^Département de Psychologie, Université de Montréal

**Introduction**: It is generally accepted that pain sensitivity is greater in women. However, sex differences in pain responses have been overlooked. The aim of this study is to examine sex differences in motor responses to pain and how it is shaped by psychosocial factors.

**Methods**: The study includes 52 women and 46 men. Pressure pain thresholds were measured to confirm the sex difference in pain sensitivity. Transcutaneous electrical stimulation of the sural nerve was used to evoke the nociceptive flexion reflex (NFR) with four stimulus intensities adjusted individually between pain and tolerance thresholds. Pain catastrophizing, fear of pain, pain vigilance, anxiety, and gender roles were measured with validated questionnaires.

**Results**: Men showed higher pain thresholds than women (p<.001). This difference was not moderated by psychosocial factors (all p’s>.12). The intensity dependent increase in NFR amplitude was significantly greater in men than women (p=.004), indicating a sex difference in motor reactivity to pain. Bonferroni-corrected planned contrasts revealed that in men, the three highest intensities induced significantly greater NFR amplitude compared with the lowest intensity (all p’s<.001). In contrast, none of these effects was significant in women (all p’s>.15). Moreover, analyses of covariance indicated that these effects remained significant after controlling for individual differences in pain and tolerance thresholds, as well as psychosocial factors.

**Discussion/Conclusions**: These results indicate that men show lower pain sensitivity than women, but greater NFR (motor) reactivity. This between-sex sensorimotor dissociation may indicate sex-specific evolutional or biological advantages. This warrants future studies to examine the underlying neurophysiological mechanisms.

## Exploring social conceptualizations of trauma to better understand chronic pain experiences among women

Laura Connoy^1^, Larissa Costa-Duarte^1^, Marilyn Ford-Gilboe^1^, Fiona Webster^1^

^1^Western University

**Introduction**: Chronic pain is disproportionately evident among women living with trauma, yet much focus remains on interpersonal trauma at the expense of social forms of trauma—like systemic and structural harm and violence—which inform chronic pain experiences. This study employs a socially informed conceptualization of trauma to better recognize the largely underrecognized knowledges and experiences of women living with chronic pain.

**Methods**: We recruited women across Ontario who are over the age of 18 and self-identified as living with chronic pain. Utilizing key tenets of the sociological approach of institutional ethnography, we conducted interviews with 9 women both in-person and via Zoom to bring into focus social relations that inform chronic pain, including institutional processes and functions. Data was discussed in smaller and larger team meetings and indexed to keep the institution in view.

**Results**: We developed four preliminary topics: 1) limitations of healthcare: quick fixes, stereotyping and dismissal; 2) economic institutions: harms in the workplace; 3) the family: relationships of distress; and, 4) what is needed: holistic methods, validation and trauma informed approaches. Strewn across these is patriarchy as a system of oppression and organizing principle of participants’ lives.

**Discussion/Conclusions**: This project highlights the need to recognize social forms of harm and violence and how these inform chronic pain. A socially informed conceptualization of trauma shifts responsibility towards institutions, rendering them responsible for the violence that can exacerbate pain. As a result, chronic pain can be understood not simply as a health issue but a social justice issue.

## Sex, Gender, and Sociodemographic Factors Associated with Persistent Use of Prescription Medication in Chronic Pain: Insights from a Cohort Study

Marimée Godbout-Parent^1^, Nancy Julien^1^, Hermine Lore Nguena Nguefack^1^, M. Gabrielle Pagé^2^, Line Guénette^3^, Lucie Blais^4^, Nancy Ménard^1^, Sylvie Beaudoin^1^, Anaïs Lacasse^1^

^1^Université du Québec en Abitibi-Témiscamingue, ^2^Centre de recherche du Centre hopitalier de l’Université de Montréal (CRCHUM), ^3^Université Laval, ^4^Université de Montréal

**Introduction**: Chronic pain (CP) disproportionately affects women and gender-diverse individuals, raising questions about how sociodemographic factors influence medication use. Yet, the interplay between sex, gender, and prescribed medication use in CP remains poorly understood, limiting optimization, safety, and equity of care. We examined how sex and gender relate to persistent use of prescribed pain medication classes among individuals with CP, accounting for sociodemographic factors.

**Methods**: This study was conducted among individuals living with CP and links self-reported data to public and private prescription claims (n=561). Persistent use (≥40% days covered; yes/no) of medications prescribed for CP and related comorbidities in the year following questionnaire completion was analyzed. Main independent variables were sex, gender identity, and gender-stereotyped personality traits (Bem Sex-Role Inventory). Cluster analysis was used to create intersecting sociodemographic subgroups (incorporating sex, gender, and other sociodemographic factors). Multivariable logistic regression was achieved to examine associations between these subgroups and persistent medication use.

**Results**: Most commonly persistently used classes of medications prescribed for CP and related comorbidities were antidepressants (48%), anticonvulsants (35%), opioids (19%), and nonsteroidal anti-inflammatory drugs (18%). Between clusters, statistically significant differences were found for the subgroups labelled: (1) ‘W*omen with private drug insurance*’, who had lower odds of persistent opioid use (aOR:0.38; 95%CI:0.15-0.95), and (2) ‘*Unemployed older men*’, who had lower odds of persistent antidepressant use (aOR: 0.45; 95%CI:0.24-0.87) (vs. ‘*Unemployed women*’).

**Discussion/Conclusions**: Our results highlight how sex, gender, and intersecting sociodemographic factors influence persistent use of prescribed medications, particularly opioids and antidepressants, among individuals living with CP.

## Imaging: Pain Imaging and Neuroimaging

### L’imagerie: l’imagerie de la douleur et la neuroimagerie

## Menstrual pain and the brain: a conditioned pain modulation fMRI pilot study

Natalie Osborne^1,2^, Sarah Darnell^1^, Dina Vavarutsos^1^, Zoey Fitzgerald Kidwell^1^, Nondas Leloudas^1^, Kevin Hellman^1,2^, Frank Tu^1,2^

^1^Endeavor Health, ^2^University of Chicago Pritzker School of Medicine

**Introduction**: Dysmenorrhea (painful periods) is a risk factor for chronic pain, but the mechanisms underlying this increased pain vulnerability are unknown. Heightened pain sensitivity to visceral and deep tissue stimuli in the trunk and - less commonly - to distal cutaneous stimuli have been found, indicating some level of altered central pain processing.

**Methods**: We developed a novel conditioned pain modulation (CPM) paradigm for functional MRI of brain activity off-menses in 34 adults with dysmenorrhea (Dys) and 10 controls. A pressure cuff on the right thigh calibrated to deliver a pressure eliciting 40/100 pain served as a test stimulus, while a footpad with circulating cold water (10°C) on the left foot served as the conditioning stimulus.

**Results**: Preliminary analysis revealed that the CPM (cold + pressure) condition inhibited pain ratings and showed greater activity in the right hippocampus and bilateral caudate nucleus compared to rest, pressure, and cold pain alone. Participants with dysmenorrhea and hypersensitivity to evoked bladder pain (DysB, n=11) - a subgroup previously shown to exhibit widespread pain sensitivity and increased risk for chronic pain - rated the cold water as more painful and had more brain activation to cold compared to Dys and controls, including in the insula, supplementary motor area, and dorsal posterior cingulate cortex. During CPM, Dys had greater anterior prefrontal cortex activation than DysB.

**Discussion/Conclusions**: This novel fMRI-CPM paradigm successfully elicited conditioned behavioural and brain responses to pain and may be useful in characterizing abnormal central pain processing in individuals at-risk for chronic pain.

## Brain Responses to Emotional Stimuli, Pain Catastrophizing and Chronic Headache in Youth

Yi An Wang^1^, Elias Abou-Assaly^1^, Inge Timmers^2^, Laura Simons^3^, Melanie Noel^1^, Jillian Miller^1^

^1^University of Calgary, ^2^Tilburg University, ^3^Stanford University

**Introduction**: Pain catastrophizing, the tendency to magnify, ruminate and feel hopeless in response to pain, is associated with increased pain and pain-related disability in pediatric chronic pain (pain > 3 months). However, little is known about how pain catastrophizing influences emotional brain responses to maintain pediatric chronic pain. This study compared the neural responses to emotional stimuli in youth with and without chronic headache and investigated whether pain catastrophizing mediated the relationship between altered brain activation and headache frequency.

**Methods**: 30 youth (ages 10-18) with chronic headache and 30 age- and sex-matched controls tracked their headache for one month. They answered the Pain Catastrophizing Scale. Functional MRI scanning was completed to detect blood oxygen level-dependent (BOLD) signal changes in response to validated pictures of facial affect. Second-level analyses were conducted to compare differences in BOLD responses between groups. In clusters showing group differences, we then examined whether pain catastrophizing mediated the relationship between brain activation and headache frequency.

**Results**: Patients showed heightened activation to emotional stimuli (i.e., happy and fearful faces versus scrambled control images), compared to controls, in the right middle temporal gyrus (cluster size = 363, FWE-corrected P = .01[IT1] [IT2]). In patients, pain catastrophizing significantly mediated the relationship between middle temporal gyral activity and headache average per day (Effect: 2.37, CI: 0.25-4.35), such greater middle temporal gyral activation was associated with greater headache average per day through its association with pain catastrophizing.

**Discussion/Conclusions**: Modifying threat value in pediatric chronic pain patients may be important to headache management.

## Disentangling periodic and aperiodic alpha activity in the dynamic pain connectome of young healthy adults

Sushmit Das^1,2^, Rima El-Sayed^1,2^, Vaidhehi Veena Sanmugananthan^1,2^, Natalie R Osborne^1,2^, Junseok A Kim^1,2^, Rachael L Bosma^1,2^, Karen D Davis^1,2^

^1^University Health Network, ^2^University of Toronto

**Introduction**: Resting-state alpha activity has been proposed to be related to acute pain sensitivity and chronic pain, including alpha power and peak alpha frequency (PAF). However, band-limited power spectral density (PSD) metrics can reflect true oscillatory peaks and/or shifts in the aperiodic (1/f-like) background. This complicates interpretation across studies, cohorts, and interventions. Here, we investigated whether PSD-based alpha metrics and spectral parameterization yield similar estimates of alpha activity, or whether they differ in ways that could change conclusions.

**Methods**: Healthy young adults (under 40 years old) underwent resting-state magnetoencephalography. Spectra were computed using a standard PSD pipeline across 19 regions of interest (ROIs) in the dynamic pain connectome spanning ascending and descending nociceptive pathways, salience and default mode networks. We applied spectral parameterization to separate the aperiodic (1/f) background from periodic alpha peaks. From both approaches, we extracted PAF and total 8-13 Hz alpha power for each participant.

**Results**: PSD- and parameterization-based estimates of PAF differed significantly across all ROIs, showing the methods are not interchangeable. PSD total alpha power also differed significantly from parameterized periodic-only alpha peak power after removing the 1/f background.

**Discussion/Conclusions**: These findings highlight the importance of spectral analysis method which can shape inferences about alpha-band activity. PSD-based alpha power effects may partly reflect background spectral changes, whereas parameterization yields separable periodic and aperiodic features that improve interpretability and comparability. Therefore, reporting both parameterized alpha peak and aperiodic measures provide key information to disambiguate alpha power and PAF from aperiodic shifts.

## Exploration of pain-related nervous system alterations in symptomatic knee osteoarthritis

Christopher Lamb^1^, Lara Boyd^1^, Jackie Whittaker^1^

^1^Faculty of Medicine, University of British Columbia, Vancouver, British Columbia, Canada

**Introduction**: Knee osteoarthritis (KOA) is a leading cause of chronic pain worldwide. Yet understanding of pain mechanisms and their relationship to cortical changes is incomplete. Previous research has used transcranial magnetic stimulation (TMS) to explore pain-related excitability changes in primary motor cortex (M1) only, despite pain affecting a broad sensorimotor network. This exploratory study aimed to conduct an expanded examination of cortical sensorimotor excitability, and its relationship to pain intensity in individuals living with KOA.

**Methods**: Thirteen individuals (9 female) with symptomatic KOA who met the National Institute for Health and Care Excellence diagnostic criteria, and 14 controls (7 female) completed the assessment protocol. Cortical excitability in bilateral M1 was assessed using TMS to index resting motor threshold (RMT). Cerebellar inhibition (CBI) to M1 was measured using a short-interval [5 ms] paired pulse paradigm. RMT and CBI protocols targeted the quadriceps representation in M1. Pain intensity was assessed using 101-numeric pain rating scale. Between group differences, and associations between pain intensity and cortical excitability within the KOA group were explored.

**Results**: Individuals with symptomatic KOA demonstrated significantly less cerebellar inhibition to M1 (higher CBI ratio) compared to controls (KOA: 1.22+/-0.42, controls 0.76+/-0.42, p<0.001). No difference in M1 excitability (RMT) between groups, or association between CBI/RMT and pain intensity in individuals with KOA was found.

**Discussion/Conclusions**: This work will inform a fully powered study to enable enhanced understanding of differences in cortical sensorimotor excitability, and relationship to pain intensity in persons living with KOA.

## Preliminary Evidence that Cold-Induced Pain Disrupts Functional Connectivity in Knee Osteoarthritis Patients with Chronic Pain: An rs-fMRI Study

Mahnaz Tajik^1,2^, Dinesh Kumbhare^3, 4^, Michael D. Noseworthy^1, 2, 5, 6, 7^

^1^Department of Medical Sciences, McMaster University, Hamilton, ON, Canada, ^2^Imaging Research Centre, St. Joseph’s Healthcare Hamilton, Hamilton, ON, Canada, ^3^Toronto Rehabilitation Institute (TRI), University of Toronto, Toronto, ON, Canada, ^4^Department of Medicine University of Toronto, Toronto, ON, Canada, ^5^Department of Electrical and Computer Engineering, McMaster University, Hamilton, ON, Canada, ^6^Department of Kinesiology, McMaster University, Hamilton ON, Canada, ^7^Department of Medical Imaging, McMaster University, Hamilton, ON, Canada.

**Introduction**: Chronic pain in knee osteoarthritis (KOA) arises from both peripheral joint pathology and central sensitization, particularly in patients with widespread, nociplastic pain. While resting-state fMRI (rs-fMRI) captures intrinsic network dysfunction, how acute pain in these patients alters connectivity remains unclear. Using a standardized Cold Pressor Gel Test (ICE) as an MRI-compatible model of nociceptive pain, this study examined network-level modulation during pain. We hypothesized that nociplastic features would associate with altered connectivity within the Default Mode, Salience, and Sensorimotor networks.

**Methods**: Eleven KOA participants with widespread pain (10 female, 1 male; mean age = 61.2 ± 7.4 years) completed three 8-minute rs-fMRI runs: baseline (Rest), cold pain (ICE), and tactile/pin stimulation. MRI data were acquired using a GE 3T MR750 and processed using CONN with field-map correction, normalization, and 8 mm FWHM smoothing. Analyses focused on ICE vs Rest (voxel p < 0.001; cluster p-FDR < 0.05). Pressure-pain thresholds (PPT) were measured pre- and post-ICE using a handheld digital algometer applied to the medial knee and wrist, with pressure gradually increased until participants indicated the first pain sensation.

**Results**: Behavioral data showed inter-individual variability in PPT, indicating mixed descending pain modulation. ROI-to-ROI analysis identified one significant cluster (F(1,5)=144.47, p-FDR = 0.013) with increased ICE > Rest connectivity between the left lateral occipital cortex and salience-network regions (ACC, anterior insula, rostral prefrontal cortex). Seed-to-voxel analyses revealed decreased Default Mode coupling between medial prefrontal and parietal cortices, and increased salience/sensorimotor connectivity with cerebellar and motor areas.

**Conclusion/Discussions**: These preliminary results suggest network-specific modulation during acute pain, consistent with nociplastic mechanisms in KOA. Recruitment and multimodal analyses are ongoing to validate and expand these findings.

## Individual Differences in Secondary Hyperalgesia and Associated Neural Activity

Kayla Millar^1^, Richard Harrison^2^, Tim Salomons^1^

^1^Queen’s University, ^2^University of Reading

**Introduction**: Central sensitization is a mechanism associated with chronic pain, and secondary hyperalgesia (SH) is a manifestation of central sensitization used to predict propensity to develop chronic pain (Nijs et al., 2021; Woolf, 2011). Previous research suggested SH is a stable physiological response and identified group differences in neural activity associated with individuals’ propensity to develop small or large areas of SH (Asghar et al., 2015; Werner et al., 2013). This study examined whether individual differences in the neural pain response were related to the area of SH individuals develop.

**Methods**: Participants (*N* = 75) completed one MRI session with thermal nociceptive stimulation and two measurements of SH within a two-week period. A multilevel model was conducted to examine individual differences in area of SH developed across sessions. A general linear model was conducted using FSL to examine whether patterns of neural activity were related to the average area of SH they developed.

**Results**: Multilevel model results suggested SH was stable within participants across sessions, however, they displayed significant interindividual differences in the area of SH developed. FSL analyses suggested larger areas of SH were associated with increased neural activity in the inferior parietal lobe and the premotor cortex.

**Discussion/Conclusions**: These findings suggest increased activation in brain regions contributing to multisensory integration, spatial representation, and motor response planning contributes to individual differences in the development of SH. Targeting aspects of sensory processing and motor responses through psychological and behavioural interventions may be effective for reducing SH and potentially, pain vulnerability.

## Laminar Profile of Pain Learning in the Insula

Gianluca Guglietti^1^, Amelia Lozano-Beckman^1^, Matt Fillingim^1^, Mathieu Roy^1^, Etienne Vachon-Presseau^1^

^1^McGill University

**Introduction**: The Reinforcement Learning (RL) model of pain suggests that pain perception is optimized to minimize future pain. This is thought to be processed in layer dependent hierarchy within the insula. This study aims to elucidate the layer specific circuitry of pain learning using a novel pain learning task and high-resolution fMRI. We hypothesize that pain perception will align with expectations during periods of high certainty and low controllability. Additionally, we expect to see activation in the dorsal posterior insula during pain assimilation and deep layers of the anterior insula during contrast.

**Methods**: Participants pick between two visual cues that control thermal stimulation to learn their associations. Associations vary in baseline certainty (odds of delivering pain) and controllability (free/forced choice). High-resolution 2D-EPI BOLD during task completion. To quantify the learning patterns, we applied the Rescorla-Wagner model. The model describes how associations strengthen or weaken after each trial represented by the Q-value. Higher Q-values represent greater preference for a given visual cue.

**Results**: Using mixed linear model across participants we observed significant negative association between Q-values and pain intensity ratings stratifying for temperatures (low temp: β=-4.770, p<0.001; high temp: β =-5.648, p<0.001). We also observed a significant interaction between uncertainty ratings and q values on pain ratings (β = -0.148, p < 0.001) with higher levels of certainty being related to greater assimilation effects of the Q-value. In addition, preliminary results suggest layerwise connectivity between superficial layers of the posterior insula and deep layer of the anterior insula in both resting and task based fMRI.

**Discussion/Conclusions**: By examining how the brain integrates sensory stimuli with past experiences and expectations to generate the experience of pain, this study aims to advance our understanding of the neural underpinnings of pain and its modulation.

## Pain in specific populations

### La douleur dans les populations distinctes

## Changes in resting state functional connectivity after intensive interdisciplinary pain treatment in youth and young adults with chronic migraine

Clara Moon^1^, Julie Shulman^1^, Allison Smith^1^, Navil Sethna^1^, William La Cava^1^, Alyssa Lebel^1^, Scott Holmes^1^

^1^Boston Children’s Hospital

**Introduction**: Clinical data and self-report of pediatric headache are largely used to support the differentiation of existing headache phenotypes. These tools can rely on subjective data and often fail to help understand differential responses to treatment. Despite evidence for several clinical phenotypes of pediatric headache, there is little information relating to objective biomarkers supporting presence and symptom changes.

**Methods**: We evaluated functional connectivity (FC) of rs-fMRI data to understand brain changes in youth with chronic migraine after participation in intensive interdisciplinary pain treatment (IIPT; n =15), compared to youth receiving usual standard care (SC; n=20). Both groups were evaluated at two time points: SC at baseline and at 3-month follow-up, and IIPT at program admission and discharge.

**Results**: Three comparisons revealed multiple FC differences. Comparing IIPT-SC baselines, IIPT showed greater FC between left frontal pole to right isthmus cingulate and right para hippocampal. IIPT from before to after intervention showed greater connectivity from left precentral to right postcentral, left lateral orbitofrontal, and right pars triangularis. Longitudinal findings of SC from baseline to follow-up visit showed greater FC between left cuneus to left lateral orbitofrontal cortex.

**Discussion/Conclusions**: Observing greater frontal-hippocampal interaction in IIPT may reflect the chronicity of pain symptoms. Despite both cohorts showing changes in orbitofrontal cortex between time points, IIPT showed greater FC with sensory and motor cortices, underscoring positive role of these regions in recalibrating pain-oriented sensory processing. Follow-up work will include concurrent structural imaging and neurobehavioral correlations.

## The Impact of the COVID-19 Pandemic on Pain in Youth with Cancer

Kayla Iasenza^1^, Alex Pizzo^1^, Paul C Nathan^2^, Thomas Hadjistavropoulos^3^, Lindsay Jibb^2^, Nicole Alberts^1^

^1^Concordia University, ^2^Hospital for Sick Children, ^3^University of Regina

**Introduction**: Pain is common in youth during and after cancer treatment. While COVID-19 impacted pain experiences and care in youth without cancer, its effects on youth with cancer remain unclear.

**Methods**: Canadian parents (N=118, median age [IQR] 40[37-46], 89.8% female) of youth (0-21) on and off cancer treatment (57.6% male, mean[*SD*] age=9[4.9]) completed measures of COVID-19 impact and distress (CEFIS), proxy reports of child pain interference (PROMIS), and child pain intensity (Numerical Rating Scale). Participants reported on pain at the time of survey completion (i.e., 2023-2024) and on the impact of the pandemic retrospectively. Hierarchical linear regression models examined associations between: (1) COVID-19 exposure/impact (i.e., exposure to COVID-19, related events; impact on family relationships, well-being) and pain interference and intensity, and (2) COVID-19 parent distress (i.e., pandemic distress) and pain interference and intensity.

**Results**: Mean scores of 49.16 (*SD*=10.35; T-score) and 1.72 out of 10 (range=0-7; *SD*=1.82), were observed for child pain interference and child pain intensity, respectively. Greater exposure to COVID-related events (e.g., stay at home orders, school closures, changes in employment) was independently associated with higher child pain interference (β=.281, p=.005). After adjusting for age and sex, COVID-19 parent distress explained additional variance in child pain intensity (ΔR^2^ = .05, p=.01) and emerged as a significant independent predictor of pain intensity (β=.232, p=.01).

**Discussion/Conclusions**: Greater COVID-19 exposure and parent distress were linked to higher pain interference and intensity in youth. Further research is needed to assess the pandemic’s broader effects on pain in pediatric cancer patients and survivors.

## Joint effect of chronic pain and SARS-CoV-2 infection severity on post-COVID complications and symptoms.

Camilla Porto Campello^1,2^, Djamal Berbiche^1,2^, Helen-Maria Vasiliadis^1,2^

^1^Faculté de Médecine et sciences de la santé, Université de Sherbrooke, ^2^Centre de Recherche Charles-Le Moyne

**Introduction**: Pain conditions have been associated with SARS-CoV-2 infection. Additionally, both pain and SARS-CoV-2 infection have been linked to a range of post-COVID symptoms. The aim was to assess the joint effect of chronic pain (CP) and SARS-CoV-2 infection severity on post-COVID complications and symptoms.

**Methods**: This study uses data from 1,871 adults with a positive SARS-CoV-2 PCR test in the “Biobanque Quebecoise de la Covid-19” (BQC19). The severity of the infection was dichotomized (mild vs moderate/severe). Data collected at baseline and at 6-month follow-up were used to assess CP (myalgia/arthralgia/headache). Study outcomes included psychiatric, neurological, cardiovascular, and gastrointestinal post-COVID complications, and COVID-related symptoms, such as mental confusion, moderate/severe anxiety/depression (MSS-ANXEP), fatigue, frailty, and sarcopenia. Logistic regression analyses were used to examine the joint effect of CP and SARS-CoV-2 severity (S) on outcomes, controlling for age, sex, recruitment site, and history of chronic conditions. A synergy index (SI) was also computed [SI= aOR_(CP+S+)_ - aOR_(CP+S-)_ - aOR_(CP-S+)_ - 1], where SI>0, SI=0, and SI<0 indicate synergistic, no, and negative interaction (less than additive), respectively.

**Results**: Close to 9.8% and 27.4% reported CP and moderate/severe infection. CP was associated with all outcomes. There was no positive synergistic joint effect of CP and SARS-CoV-2 severity on post-COVID complications, fatigue, and MSS-ANXEP. There was a negative synergistic effect on frailty and sarcopenia.

**Discussion/Conclusions**: CP is associated with post-COVID complications, independent of SARS-CoV-2 severity. The negative synergy observed suggests that CP and infection severity share similar etiologic pathways for frailty and sarcopenia.

## The association between pain and traumatic memories in critically ill survivors: Does gender matter?

Céline Gélinas^1,2^, Robin Kagie^2^, Bachi-Ayukokang Ebob-Anya^1,2^, Alice Wagenaar^3^, Lan-Vy Ho^1,2^, Grace Al Hakim^1^, Geneviève Laporte^1,2^

^1^Ingram School of Nursing, McGill University, Montreal, Canada, ^2^Centre for Nursing Research, Jewish General Hospital, Montreal, Canada, ^3^Association québécoise de la douleur chronique, Montreal, Canada

**Introduction**: Most critically ill survivors experience pain and >20% will suffer from post-traumatic stress including traumatic memories from their stay in the Intensive Care Unit (ICU). We aimed to explore the relationship between ICU pain and traumatic memories at 3 months post-discharge and the influence of gender in this multisite study.

**Methods**: We conducted an observational study design in 5 mixed ICUs in the province of Quebec. Eligibility required an ICU stay of over 36 hours, reported pain, and able to communicate in French or English. Participants reported their average pain using the 0-10 Numeric Rating Scale and the 4 ICU traumatic memories of the Post-Traumatic Stress Syndrome Inventory.

**Results**: Out of 255 participants, 65.5% identified as man and 34.5% as woman with mean age of 62 (SD=13 years). They were admitted for a surgical (68%), medical (21%) or trauma (11%) diagnosis with a median ICU stay of 4 days (IQR=2-6) and the majority were mechanically ventilated (68%). ICU average pain had a median of 4 (IQR=2-5) and 42% reported at least one traumatic memory (nightmares 16%, severe panic 23%, severe pain 27%, and suffocation 19%). ICU average pain was positively correlated with the number of traumatic memories (rho=0.24, p<.001). Women reported higher ICU pain (median=5 vs 4; Mann-Whitney U test, p=.002) and at least one traumatic memory compared to men (55% vs 36%; Chi-Square test, p=.04).

**Discussion/Conclusions**: Women experienced higher ICU pain and traumatic memories post-discharge. Individualized pain and stress management are essential to optimize recovery.

## Observational study to access the need and pattern of analgesics prescribed at discharge for patients undergoing major cancer surgery.

Sumitra Bakshi^1^, Bhakti Deshmukh^2^, Vaibhav Patil^1^, Supriya Salunkhe^1^

^1^Tata Memorial Hospital and Homi Bhabha National Institute, INDIA, ^2^ACTREC and Homi Bhabha National Institute, INDIA

**Introduction**: With enhanced recovery after surgery (ERAS) pathways enforced in the healthcare system, patients are benefiting by early discharge post major surgeries. Hence, It is essential to optimally treat pain even after discharge. This study was undertaken to understand the need and pattern of analgesics prescribed at discharge following major onco-surgeries.

**Methods**: Following approval from ethics board and registration of trial, patients planned for discharge within 96 hours after a major elective oncology surgery were enrolled in this observational study after taking valid informed consent. Each patient was given hard copy of Brief Pain Inventory (BPI) and the assessment of pain was done either in person or telephonically after 7 days of discharge. Data regarding severity of pain, compliance with the prescription, requirement of rescue analgesics and need of any other medications/treatment (apart from prescription) was noted.

**Results**: In this ongoing study, total 145 postoperative patients were screened and 120 patients were recruited. Twelve patients (10%, CI 0.05 to 0.17) reported moderate to severe pain, 16 reported no pain, 92 had mild pain, one week after discharge. At the time of evaluation, 47 patients still required rescue analgesics. Two patients needed additional analgesics than the prescribed medications.

**Discussion/Conclusions**: Although most patients reported only mild pain, 10% experienced moderate to severe pain at one-week post-discharge despite receiving scheduled analgesics. Opioids were prescribed in 2.5% of patients, which is lower than international data. In conclusion, although most patients reported only mild pain one week after discharge, select groups may benefit from a more tailored approach.

## Catastrophizing as a Target for Improving Pain Outcomes Following Health-Related Pandemics

Antonina Pavilanis^1^, Wenny Fan^2^, Christiane Konstantopoulos^1^, Heewon Jang^1^, Madeline Dowd^1^, Michael Sullivan^1^

^1^McGill University, ^2^University of Alberta

**Introduction**: Large-scale infectious disease outbreaks are often followed by persistent symptoms, elevated pain, and heightened perceptions of threat. Cognitive-emotional processes such as catastrophizing and post-traumatic stress symptoms (PTSS) have been identified as risk factors for worse recovery outcomes across health conditions. However, their specific contribution to pain following pandemic illness remains unclear. COVID-19 offers a useful model, as a considerable number of individuals report ongoing musculoskeletal, neuropathic, or widespread pain following infection. The purpose of the present study was to examine the associations among PTSS, catastrophizing, and persistent pain following COVID-19, and to determine whether catastrophizing explains unique variance in pain beyond PTSS.

**Methods**: The study sample consisted of 200 individuals who were assessed 30-120 days following confirmed COVID-19 infection. Measures of PTSS, symptom catastrophizing, and pain intensity were administered at enrolment and 6 months later.

**Results**: Consistent with previous research, PTSS and symptom catastrophizing were strongly associated with each other (*r* = .596, *p* < .001) and with pain at both baseline and follow-up (ˆ*rs* = .489-.556, *ps* < .001). Hierarchical regressions showed that symptom catastrophizing explained unique variance in pain beyond PTSS, increasing explained variance from 24% to 35%, and emerging as the strongest individual predictor.

**Discussion/Conclusions**: Catastrophizing appears to place individuals at greater risk for higher and more persistent pain following pandemic-related illness. It may contribute to cycles of increased symptom monitoring and perceived threat, beyond the influence of trauma-related distress. These findings suggest that catastrophizing is a clinically meaningful and potentially modifiable factor in post-pandemic pain. Though drawn from COVID-19, these findings may generalize future health emergencies.

## The Influence of Gestational and Postnatal Age on Neonatal Pain Response During a Routine Heel Lance in the NICU: An HRV-Based Assessment

Lucas De Araujo^1^, Sara Jasim^1^, Carol Cheng^2^, Vibhuti Shah^2^, Rebecca Pillai Riddell^1^

^1^York University, ^2^Mount Sinai Hospital

**Introduction**: Preterm infants in the neonatal intensive care unit (NICU) are exposed to recurrent stressors during a critical window of autonomic development, including medically necessary procedures. Despite their central role in neonatal development, few studies have examined the combined influence of gestational age (GA) and postnatal age (PNA) on neonatal pain regulation. Infants born earlier often remain in the NICU longer and may be more physiologically vulnerable due to their immature autonomic systems and cumulative stress exposure. The goal of this analysis is to examine the influence of GA and PNA on low-frequency heart rate variability (LF-HRV) during a routine heel lance.

**Methods**: Infants (N = 52) were observed during a routine heel lance procedure at Mount Sinai Hospital NICU, with cardiac activity recorded via ECG monitoring. LF-HRV was assessed across four 30-second epochs: baseline (60-29 seconds pre-lance), reactivity (0-30 seconds at lance), and two regulatory phases (60-89 seconds and 120-149 seconds post-lance).

**Results**: Three-step hierarchical regression models across epochs showed that GA and PNA predicted LF-HRV, at baseline and during the first regulatory phase (lower GA and higher PNA was associated with a heightened physiological response). Descriptive graphs revealed varying LF-HRV patterns across developmental groups, providing partial support for Allostatic Load Theory, as low GA and high PNA did not consistently demonstrate greatest dysregulation.

**Discussion/Conclusions**: These findings highlight the complexity of preterm pain regulation and underscore the need for multimodal approaches to comprehensively capture the preterm pain experience.

## Emotional Experiences of First-time Parents Engaging in Skin-to-Skin Care and Skin-to-Skin Contact for the Management of Acute Pain of Very and Extremely Preterm Infants

Estreya Cohen^1^, Haleh Hashemi^1^, Nichaela Garvey^1^, Fabiana Bacchini^2^, Lesley Johannsson^3^, Carol Cheng^3^, Vibhuti Shah^3^, Rebecca Pillai Riddell^1^

^1^York University, ^2^Canadian Premature Babies Foundation, ^3^Mount Sinai Hospital

**Introduction**: Skin-to-skin care (SSC) and skin-to-skin contact for procedural pain (SSCP) are well-established interventions in the neonatal intensive care unit (NICU) with demonstrated benefits, including pain reduction in infants. However, limited research has explored how first-time birthing parents of very and extremely preterm infants (V/EPT; < 32 weeks gestational age), a group for whom SSCP can be particularly stressful, experience these interventions. This is a sub-analysis of data collected to understand maternal experiences related to SSC and SSCP in the NICU with their V/EPT infants (Hashemi et al., 2025).

**Methods**: Virtual interviews were completed with 38 mothers of V/EPT infants across Canada, whose babies had been admitted to the NICU within the past five years. The interview transcripts were then analyzed following 6 phases of thematic analysis.

**Results**: Data was synthesized around themes relating first to emotional experience with SSC broadly and then SSCP both at first attempt and over time. The experiences of first-time parents were then compared to those of parents who already have children.

**Discussion/Conclusions**: Around half (53%) of mothers interviewed were first-time parents when their child was admitted to the NICU. Overall, mothers’ experiences with SSC and SSCP revealed an emotionally rich but complex process. First-time mothers described SSC and SSCP as affirming experiences; they finally felt like parents when they held their infants and participated in their care. Understanding the emotional experiences of first-time parents with SSC and SSCP is essential to better support them in their V/EPT infants’ pain management in the NICU.

## Examining Very Preterm Infants’ Autonomic Pain Responding During Heel Lance in the NICU: The Role of Maternal Physiological Arousal and NICU-Related Stress

Divya Bhupal^1^, Oana Bucsea^1^, Vibhuti Shah^2^, Carol Cheng^2^, Rebecca Pillai Riddell^1^

^1^York University, ^2^Mount Sinai Hospital

**Introduction**: Preterm infants in the neonatal intensive care unit (NICU) undergo repeated painful procedures while autonomic regulation is still maturing. During skin-to-skin contact (SSC), caregivers may buffer infant pain-related physiology, but caregiver stress may interfere with co-regulation. This study examined whether maternal physiological arousal and NICU-related psychological stress surrounding a routine painful procedure predicted preterm infants’ heart rate (HR) post-lance, an index of pain-related distress.

**Methods**: Mother-infant dyads (n = 36) were observed in SSC during heel lance in a tertiary Level III NICU. Maternal and infant HR were captured via ECG. Infant HR post-lance epochs were the primary outcomes: reactivity (0-29 seconds[s] post-lance) and regulation (60-89 s post-lance). Maternal predictors were stress arousal prior to the heel lance (HR, 3 X 30 second epochs prior to lance and then 30-second epochs post-lance that were prior or concurrent) and the PSS:NICU (parent stress scale) . Hierarchical regressions controlled for infant gestational age (GA) and postnatal age (PNA). Modifications were made based on assumption violations.

**Results**: No maternal predictors or infant age variables were significant for HR reactivity. In contrast, GA was the only significant predictor of HR during the early regulation phase. Greater GA predicted higher HR (β = 0.33, p = 0.047).

**Discussion/Conclusions**: Findings suggest a difference between acute reactivity and initial regulation during heel lance performed while in SSC. Initial regulation may be more strongly influenced by maturational factors, underscoring GA as a key predictor of early post-lance autonomic regulation.

## Cannabis as a Management Strategy for Fibromyalgia: A Qualitative Study of Canadian Women

Samantha Holmes^1^, Melissa Northwood^2^, Nancy Carter^2^, Jason Busse^2^

^1^University of Manitoba, ^2^Mcmaster University

**Introduction**: Fibromyalgia (FM) is a complex chronic pain condition that affects approximately 3% of Canadians, most often women over the age of 40. Traditional pharmacological treatments frequently fail to provide adequate symptom relief, leading many individuals to explore alternative therapies, such as cannabis. Despite increasing legalization and accessibility, little is known about the experiences of individuals with FM who use cannabis for symptom relief.

**Methods**: We used a qualitative descriptive design involving fifteen adult women with FM across six Canadian provinces. Participants were recruited through purposive sampling to share their lived experiences with current or past cannabis use. Data was collected via semi-structured Zoom interviews and analyzed through reflexive thematic analysis.

**Results**: Findings were categorized into four major themes: (1) Cannabis as a Core Component of Management, described as a “turning point” for pain relief, improved sleep, and quality of life; (2) Barriers and Challenges, including stigma, lack of healthcare provider support, financial constraints, and the difficulty of dose-finding; (3) Cannabis Use in Daily Life, highlighting intentional strategies and self-monitoring to balance symptom relief with daily responsibilities; and (4) Advice for Healthcare Professionals, emphasizing the urgent need for nonjudgmental, patient-centered care.

**Discussion/Conclusions**: These findings highlight the significant role cannabis plays in FM management while exposing systemic barriers. There is a clear need for improved provider education, enhanced affordability, and a shift toward collaborative care models. Addressing these gaps allows healthcare professionals to better support individuals with FM in making informed, therapeutic decisions.

## Pain and Post-traumatic Stress in Adult Survivors of Childhood Cancer

Aliaksei Fiodarau^1^, Claire R. Galvin^1^, Paul C. Nathan^2^, Lindsay Jibb^2^, Nicole M. Alberts^1^

^1^Concordia University, ^2^The Hospital for Sick Children

**Introduction**: Post-traumatic stress symptoms (PTSS) and chronic pain commonly co-occur in the general population, yet little is known about their association in childhood cancer survivors, despite survivors’ elevated risk for both.

**Methods**: A sample of 85 adult survivors (83.5% female, median[range] 25 [18-67] years, 20 [2.3-67.3] years since diagnosis), completed validated self-report measures of PTSS, pain interference (7-item mean), anxiety (7-item mean), depression (8-item mean), fear of cancer recurrence (9-item mean), and chronic pain (pain lasting ≧3 months). Independent samples t-test compared mean levels of PTSS between survivors with and without chronic pain. Hierarchical multiple regression was used to examined associations between PTSS and pain interference as well as psychological factors among survivors with chronic pain.

**Results**: 42.3% (36/85) of survivors reported chronic pain, of whom 25% reported moderate to severe pain interference. The mean level of PTSS among all survivors was 18.9 (SD=14.8). Higher levels of PTSS were observed in survivors with chronic pain (M=22.6, SD=14.7) compared to survivors without (M=16.2, SD=14.4; p=.02). After adjusting for age, sex, anxiety and depression, PTSS was not associated with pain interference among survivors with chronic pain (R^2^ = .406, F[5,30]=4.1, p=.55).

**Discussion/Conclusions**: These findings replicate our prior work showing a high prevalence of chronic pain in adult survivors of childhood cancer. Additionally, they provide cross-sectional evidence that elevated PTSS is associated with chronic pain in this population. Further research is needed to elucidate the nature of the relationship between PTSS and pain, which will help inform psychosocial screening and intervention for survivors.

## Exploring emotion regulation and substance use from adolescence to young adulthood in individuals with chronic pain: A thematic analysis

Randa Elgendy^1^, Michelle Gagnon^1^

^1^University of Saskatchewan, Department of Psychology and Health Studies

**Introduction**: Difficulties with emotion regulation are related with increased pain and substance use among adults; however, little is known about the relationship among emotion regulation, pain, and substance use in the period from adolescence to young adulthood. We investigated whether emotion regulation strategies influence the association among chronic pain and cannabis and alcohol use, as well as how this relation changes as adolescents age into young adulthood.

**Methods**: Young adults aged 18-25 (*N* = 20, *M*_age_= 22.05, 50% women) were interviewed regarding their experiences with pain, alcohol and cannabis use, and emotion regulation from adolescence until the present day. Interview data were analyzed using thematic analyses.

**Results**: Generated themes reflected four patterns of experience among youth: 1) alcohol and cannabis were used to reduce negative emotions and decrease pain; 2) cannabis was used to decrease pain, followed by the use of other adaptive emotion regulation strategies; 3) alcohol was used to decrease negative emotions, but led to increased pain; and 4) alcohol led to worsening of both pain and negative emotions. Two overarching trajectories across development were identified, demonstrating that either a decrease in pain intensity or development of more helpful emotion regulation skills were each associated with a decrease in alcohol and cannabis use as adolescents aged.

**Discussion/Conclusions**: This study provides preliminary evidence that experiences of chronic pain, substance use, and emotion regulation are interrelated during the transition to adulthood and may present a viable avenue for intervention and prevention of long-term substance use among individuals with pain.

## Use of Virtual Reality for Dental Fear, Anxiety and Pain in Children Undergoing Dental Treatments (VR-TOOTH): A Randomized Controlled Trial

Julien Gardner^1^, Vallerie Markopoulos^2^, Charlotte Fafard^1^, Daphnée Pelletier^1^, Marie-Eve Asselin^2^, Jean Théroux^3^, Sylvie Le May^1^

^1^University of Montreal, ^2^CHU Sainte-Justine Dental clinic, ^3^CHU Sainte-Justine Research Centre

**Introduction**: Dental fear, anxiety (DFA) and pain impact many children, especially those with special healthcare needs (SHCN), often leading to avoiding dental visits and behavior issues. Virtual reality (VR) provides an environment that may lessen DFA, pain and improve the dental experience.

**Objectives**: To evaluate the efficacy of VR in reducing DFA and pain among SHCN pediatric patients undergoing dental procedures. To assess children’, parents’ and healthcare providers’ satisfaction.

**Methods**: Participants aged 6 to 17 years were recruited from a hospital pediatric dental clinic and randomized to control group (muted-cartoons on a TV) or experimental group (VR). Primary outcome: Risk of anxiety measured by the Venham Anxiety Rating Scale. Secondary outcomes: pain, behavior, side effects and satisfaction. Subgroup analyses were planned for sex and neurodiversity.

**Results**: We recruited 385 participants. Mean age was 10.6 ± 3.1 years, predominantly boys (57%). VR group showed a lower risk of anxiety during procedure (OR: 0.391, *p* = 0.010), lower pain (*p* = 0.002), and good behavior (*p* = 0.041). No differences in side effects between groups (p=0.638). Satisfaction was high regarding VR among children, parents and healthcare professionals (> 8.5/10). Neurodivergent participants in the VR group showed lower anxiety (*p* = 0.010) and better behavior (*p* = 0.006).

**Discussion/Conclusions**: Virtual reality is an efficacious and safe non-pharmacological method to manage anxiety, pain and improve behavior in children undergoing dental procedures, especially for neurodivergent children. We are planning the implementation of VR at this dental clinic.

## Examining 1-Month Pain-Related Anxiety as a Mediator of the Relationship Between Acute Post-Operative Pain-Related Anxiety and Child and Parent 2-Month Memory Explanation

Anna Waisman^1,2^, Melanie Noel^3,4^, Chenyue Zhang^1^, Valeria Robles Vera^3^, Batsheva Weinberger^1^, Carmel Daskalo^5^, Maria Pavlova^6^, Joel Katz^1,2^

^1^York University, ^2^Toronto General Hospital, ^3^University of Calgary, ^4^Alberta Children’s Hospital, ^5^McGill University, ^6^University of Guelph

**Introduction**: Recalling specific pain-related memories protects against chronic pain in adults. In pediatric contexts, parent–child reminiscing about pain containing fewer explanatory elements has been linked to poorer developmental outcomes. However, links between autobiographical memory and pain outcomes have not been investigated in youth.

**Methods**: Following REB approval and informed assent/consent, 68 youth (10-18 years) scheduled for spinal fusion at Alberta Children’s Hospital participated. Children reported pain-related anxiety (0–10 NRS) on the first postoperative day. At 1 month they recalled the pain-related anxiety experienced on Day 1. Memory interviews occurred 2 months after surgery when youth and their parents recalled the child’s experiences on their first postoperative day. Interviews were coded for episodic specificity and categorized into thematic domains.

**Results**: A nonparametric bootstrap mediation analysis (5000 simulations) revealed a significant indirect effect of Day 1 child pain-related anxiety on parents’ explanatory details at 2 months via 1-month child pain-related anxiety recall (ACME=−0.129, *p*=0.020, 95% CI[−0.318, −0.018]). The direct and indirect effects operated in opposite directions. Day 1 child pain-related anxiety directly predicted greater use of explanatory details in parents’ narratives (ADE=0.421, *p*<0.001), while the indirect pathway through 1-month child pain-related anxiety recall significantly attenuated this effect.

**Discussion/Conclusions**: Children’s pain-related anxiety on Day 1 after surgery may catalyze family meaning-making, reflected in increased explanatory content in parents’ narratives. However, pain-related anxiety recalled at 1 month appears decoupled from the parents’ explanatory memories. These findings identify the 1-month postoperative period as a potential window for intervention, warranting further investigation.

## Mapping Literature on Pain in Migrant Youth: A Rapid Scoping Review

Mica Marbil^1^, Josep Roman-Juan^1^, Safira Dharsee^1^, Kelly Nguyen^2^, Prachi Khanna^3,4^, Megan MacNeil^4,5,6^, Sean Lindsay^1^, Diane Lorenzetti^7^, Melanie Noel^1^, Kathryn Birnie^1, 4, 6^

^1^Department of Psychology, University of Calgary, ^2^Lawrence Bloomberg Faculty of Nursing, University of Toronto, ^3^London School of Hygiene and Tropical Medicine, London, UK, ^4^Chronic Pain Network, McMaster University, ^5^School of Public Health, University of Alberta, ^6^Department of Anesthesiology, Perioperative, and Pain Medicine, University of Calgary, ^7^Health Sciences Library, Department of Community Health Sciences, University of Calgary

**Introduction**: Migrant youth often experience intersecting oppressions (e.g., racism, poverty, discrimination) that contribute to pain disparities. Despite rising global rates of migration, migrants are underrepresented in pain research, hindering efforts toward equitable and evidence-based pain care. In response to increasing anti-migrant policies and rhetoric, we conducted a rapid scoping review mapping research on pain in migrant youth.

**Methods**: Guided by PCC (population, concept, context) and PROGRESS-Plus frameworks, electronic searches were conducted in MEDLINE, CINAHL, and Scopus for primary research studies published since 2015 that examined any pain among migrant youth (aged <18 years). Data on study characteristics, sociodemographic and pain information, and systemic and sociocultural factors were charted.

**Results**: Of 7,348 unique titles/abstracts screened, 90 full-texts were reviewed with 35 final eligible articles. Most were quantitative (74%) and conducted in Europe (40%) and Western Asia (29%). Almost one-third focused on Syrian refugee youth. Samples most often involved youth aged 12-18 years. Most common types of pain were headache and migraine (31%), bodily/musculoskeletal pain (29%), and dental pain (26%). Over half of the articles (51%) did not include pain intervention, and only one examined resilience.

**Discussion/Conclusions**: Research on pediatric pain in migrants predominantly focuses on refugee populations and frames pain through forced displacement and health disparities. While important, this represents a limited view of pain among migrants, with opportunities to broaden application of diversity-/strengths-based lenses. More research exploring pain experiences among migrant youth is needed to better understand pain among this population and inform equitable and effective pain care for all.

## The Effects of Cannabis Use on Postoperative Pain in Patients Undergoing Hip and Knee Arthroplasty

Michael Smyth^1^, Jack Wile^1^, Pearla El-Rabahi^1^, Karim Mukhida^1^

^1^Dalhousie University

**Introduction**: Cannabis use is increasing in Canada, with literature suggesting potential links to heightened postoperative pain. This study evaluated the impact of preoperative cannabis use on postoperative pain, opioid consumption, and nausea and vomiting (PONV) following total hip and knee arthroplasty.

**Methods**: This retrospective study analyzed 596 patients (October 2022-January 2025). Participants were categorized as cannabis users (CU) or non-users via self-report. Primary outcomes were peak post-anesthesia care unit (PACU) pain scores and total PACU opioid consumption (oral morphine equivalents [OME]). Secondary outcomes included PONV.

**Results**: Thirty-seven patients reported cannabis use. No significant differences were found in PACU pain scores in unadjusted (B = −0.88, 95% CI −1.81 to 0.06, p = 0.67) or covariate-adjusted analyses (B = −0.79, 95% CI −1.74 to 0.17, p = 0.107). Although mean pain scores were numerically higher among CU (6.7 ± 2.8 vs. 5.8 ± 3.0), this trend was not statistically significant. PACU OME also showed no significant differences (adjusted B = −2.64, p = 0.656). While unadjusted analysis suggested increased PONV odds for CU (OR = 1.96, p = 0.048), significance was lost after adjustment (OR = 1.76, p = 0.106).

**Discussion/Conclusions**: Preoperative cannabis use was not associated with statistically significant differences in postoperative pain, opioid consumption, or PONV following total hip or knee arthroplasty.

## Examining the association between neonatal pain burden and maternal NICU stress

Lojain Hamwi^1^, Vibhuti Shah^2^, Rebecca Pillai Riddell^1^

^1^York University, ^2^Mount Sinai Hospital

**Introduction**: Preterm infants in neonatal intensive care units (NICUs) undergo frequent painful procedures. Although neonatal stress exposure has been associated with maternal psychological distress, less is known about whether quantified cumulative medical pain burden predicts NICU-specific caregiver stress.

**Methods**: Thirty-five preterm infant-mother dyads were included. Infants had a mean gestational age (GA) of 29.05 weeks (SD = 1.34) and studied at a mean post-menstrual age (PMA) of 32.08 weeks (SD = 1.86). Cumulative neonatal pain burden was derived from medical chart review and indexed using a weighted 5-point procedural pain scale capturing frequency and severity of documented painful interventions. Mothers completed the Parental Stressor Scale NICU during a routine heel lance. Items were rated on a 5-point scale, and mean stress ratings across endorsed items were calculated to generate a total stress score. Hierarchical regression examined whether pain burden predicted maternal NICU stress after accounting for GA and PMA.

**Results**: Pain burden demonstrated variability (*M* = 338.31, SD = 155.28) and maternal NICU stress levels were moderate (*M* = 3.03, SD = 0.85). In Block 1, GA and PMA did not significantly predict maternal NICU stress, *F*(2, 32) = 0.03, *p* = .969, *R*^2^ = .002. Adding cumulative pain burden in Block 2 did not account for additional variance in maternal stress (ΔR^2^ = .003, *p* = .746). Pain burden was not a significant predictor of maternal stress and the overall regression model remained non-significant.

**Discussion/Conclusions**: Findings suggest that the relationship between neonatal pain exposure and caregiver NICU distress may be more complex and moderated by individual, environmental, or psychosocial factors. Directly assessing caregiver stress in the NICU rather than inferring risk from infant medical history remains essential.

## Does Maternal Stress in the Neonatal Intensive Care Unit Impact Mother-Infant Physiological Attunement During a Stressor?

Ilana Shiff^1^, Carol Cheng^2^, Vibhuti Shah^2^, Rebecca Pillai Riddell^1^

^1^York University, ^2^Mount Sinai Hospital

**Introduction**: Mother-preterm infant physiological attunement reflects the coordination of dyadic regulation during infant distress (Hofer, 2010). Elevated parental stress may disrupt these processes (Di Lorenzo-Klas et al., 2023), particularly in the high-stress NICU environment. This study examined whether maternal NICU-related stress was associated with maternal and infant heart rate (HR) attunement during a routine painful procedure.

**Methods**: Participants were 31 mother-preterm infant dyads recruited from the NICU at Mount Sinai Hospital (Toronto). Maternal and infant HR were assessed during a routine heel lance and averaged over 30-second epochs from one minute prior to the lance through six minutes following band-aid application. Maternal stress was measured using the PSS:NICU and categorized for descriptive visualization based on the sample distribution.

**Results**: PSS:NICU mean scores ranged from 1.25 to 4.64 (M = 3.07, SD = 0.87). Five mothers scored ≥1 SD above the mean, twenty within one SD of mean, and six ≤1 SD below the mean. Compared to the other infants, infants of mothers with elevated stress appeared to show greater HR reactivity immediately following the lance and a distinct recovery pattern across subsequent epochs. Mothers in the elevated stress group also appeared to maintain higher HR across epochs.

**Discussion/Conclusions**: Elevated maternal NICU-related stress may be associated with differences in dyadic physiological responding during infant distress. These findings highlight the potential role of parental stress in shaping early regulatory processes in the NICU and may have implications for interventions.

## An investigation of the predictive relations between cardiac stress measures and neonatal clinical variables during heel-lances

Oana Bucsea^1^, Carol Cheng^2^, Vibhuti Shah^2^, Rebecca Pillai Riddell^1^

^1^York University, ^2^Mount Sinai Hospital

**Introduction**: Although past research has used measures of parasympathetic nervous system (PNS) activity to index pain-related stress regulatory patterns in hospitalized newborns, highly heterogenous response patterns have emerged in this population. Further research is needed to understand factors contributing to variable pain-related stress responses in hospitalized newborns to subsequently incorporate these non-invasive and accessible measures in pain assessment tools. The aim of the current study was to examine the predictive associations among PNS measures and clinical variables during a heel-lance in preterm newborns.

**Methods**: High-frequency heart rate variability (HF-HRV), indexing PNS activity, was measured at four epochs: pre-handling (120sec before nurse handling), reactivity (0-60sec post-lance), early recovery (0-60sec post band-aid application), and late recovery (5-6 minutes post band-aid application) in 78 hospitalized preterm newborns. Clinical variables (gestational and postnatal age, birthweight, weight at study) were collected from medical charts. Linear regressions were used to examine the predictive relationships between measures of HF-HRV and clinical variables across the four epochs.

**Results**: HF-HRV during each epoch was positively predicted by preceding HF-HRV values (*β’s* 0.29-0.51, *p’s* <.01). Baseline HF-HRV was also positively predicted by gestational age (*β* = 0.28, *p* = .05).

**Discussion/Conclusions**: Higher HF-HRV values (indicating more parasympathetic activation) predicted higher parasympathetic influences and thus more optimal regulatory patterns at subsequent epochs during a heel-lance, underscoring the importance of assessing and managing stress even prior to the procedure. Lower gestational age newborns are more likely to experience higher stress pre-handling, which can influence their subsequent patterns of responding during painful procedures.

## Highlighting the lived experiences of Veterans/Injured Military Personnel living with chronic pain and PTSD/OSI; an interpretive description study

Joline Attalla^1^, Joy MacDermid^1^, Nicholas Halmasy^1^, Shannon Killip^1^, Don Richardson^1^, Christina Ziebart^1^, Robin Campbell Bromhead^1^

^1^Western University

**Introduction**: Operational Stress Injuries (OSI) involve a range of persistent psychological difficulties which result from duties associated with a person’s occupation, including anxiety disorders, depression, and post-traumatic stress disorder (PTSD). Often, these arise after a traumatic event, combat, or high stress situation.

**Methods**: Interviews were conducted with former or current members of the Canadian Armed Forces with an OSI. 28 participants (15 men and 13 women) and were interviewed using a semi-structured interview guide. Data analysis was performed using an inductive thematic analysis, where codes and themes were derived from the data using an interpretive description approach.

**Results**: Participants provided information surrounding 4 key areas including their experience with chronic pain, stress responses related to their OSI, preferences and facilitators, feedback on exercise progression and training. Many participants noted days of extreme opposites - days where they are unhindered by pain and days where they are unable to function due to it. Participants also underlined specific triggers, where avoidance-based strategies were often used to manage symptoms. The need for social support was also mentioned, where participants often relied heavily on their spouse and finding camaraderie through other Veterans, a key barrier for rural participants was lack of access to care.

**Discussion/Conclusion**: For those living with chronic pain and posttraumatic stress disorder (PTSD) or other occupation stress injuries (OSI), they have notable challenges and struggle to resume a new pattern of physical activity and exercise. The importance of program tailoring as a result may be a key management technique through a trauma informed approach.

## Pharmaceutical and Non-pharmaceutical Interventions for Chronic Pain Following Breast Cancer Treatment: a Scoping Review

Annika Bey^1^, Yangqianxi Wang^2^, Serena Wei^3^, Kaitlyn Phan^3^, Li Wang^2,4^

^1^Michael DeGroote School of Medicine, McMaster University, Hamilton, ON, Canada, ^2^Department of Health Research Methods, Evidence, and Impact, McMaster University, Hamilton, ON, Canada, ^3^Faculty of Health Sciences, McMaster University, Hamilton, ON, Canada, ^4^Department of Anesthesia, McMaster University, Hamilton, ON, Canada

**Introduction**: Chronic pain after breast cancer (BC) treatment affects up to 60% of patients and can significantly impair wellbeing. Management approaches and outcome measures in trials vary widely.

**Objectives**: This study aims to characterize the current research using any intervention for post-BC chronic pain by analyzing treatment type, origin country, publication year, and outcome measures reported.

**Methods**: We conducted a scoping review of randomized controlled trials (RCTs) evaluating all interventions that compared with usual care, placebo, or another active treatment for post-BC chronic pain (pain ≥3 months). We searched EMBASE, MEDLINE, CINAHL, AMED, PsycINFO, and CENTRAL. Paired reviewers independently screened trials, assessed the risk of bias, and extracted data. Collected data included country of origin, publication year, types of interventions and controls, and all outcome measures as guided by IMMPACT. Data was narratively analyzed.

**Results**: Of 3752 studies screened, 62 proved eligible. Studies originated from 19 countries and publication year spanned from 1996-2025, with 80.65% in the last 10 years. Exercise and physiotherapy programs were the most common intervention (37.1%), followed by pharmacotherapy (16.13%) and psychotherapy (16.13%). All studies (100%) reported pain outcomes, 48.39% assessed quality of life, 42.94% and 24.19% reported physical and emotional functioning, respectively. Other outcomes, including sleep, cognitive functioning, social functioning, and adverse events were underreported.

**Discussion/Conclusions**: Trials on post-BC chronic pain treatment encompass a wide breadth of interventions and heterogeneous outcome measures. This provides a guide for an upcoming systematic review and network meta-analysis to assess intervention effectiveness.

## Financial burden of chronic pain after critical illness: A portrait of monthly out‑of‑pocket expenses and associated financial stress

Geneviève Laporte^1,2^, Céline Gélinas^1,2^

^1^Ingram School of Nursing, McGill University, ^2^Centre for Nursing Research and Lady Davis Institute, Jewish General Hospital

**Introduction**: About one‑third of critically ill survivors will develop chronic intensive care-related pain (CIRP), which can hinder daily activities and foster financial stress through reduced income and higher out‑of‑pocket expenses. Our aim was to describe quantitatively the financial burden reported by survivors living with CIRP, and to assess their level of financial stress.

**Methods**: We conducted a descriptive-correlational design study using validated questionnaires administered by phone. Critically ill survivors who experienced pain >3/10 at least 3 months post-discharge from the intensive care unit (ICU) provided an estimate of pain-related expenses in the last month (CoPaQ) and completed measures of financial stress and coping (COST-FACIT), pain intensity (BPI) and quality of life (SF-12).

**Results**: Participants (n=22; 54% male, 81% French-speaking) reported a mean pain intensity of 4.24/10 (SD = 2.4). They spent on average 574.45$CAD/month in healthcare costs and reported moderate financial stress (M=33.5/60, SD=5.5). Paramedical services (n=6, M=370.00$), home support services (n=8, M=227.50$) and prescription medications (n=16, M=127.20$) were among the costliest recurrent expenses. Higher pain intensity at 3 months correlated with higher monthly expenses (rs=0.55, p=0.01). Participants used more cost-reducing strategies when they reported greater financial stress (rs=0.67, p<0.001) or high pain-related expenses (rs=0.67, p<0.001). Increased financial stress was associated with lower quality of life (rs=-0.48, p=0.02).

**Discussion/Conclusions**: Survivors living with CIRP are still subjected to out-of-pocket expenses, which can contribute to significant financial stress for themselves and their family. Therefore, healthcare providers should consider the economic burden associated with CIRP treatments and integrate cost‑sensitive care options to their recommendations.

## Perceptions of the Independent Medicolegal Examination through the lens of People with Chronic Pain.

Rodrigo Deamo Assis^1^, Eric Chaize^1^, Mary-Ann Fitzcharles^2^

^1^Centre Intégré de Santé et Services Sociaus de l’Abitibi-Témiscamingue, ^2^McGill University

**Introduction**: An independent medicolegal examination (IME) may be requested regarding questions of diagnosis, severity of illness, causation or functional ability. Most commonly requested by an insurer, IMEs may also be requested by the plaintiff. Engagement with the legal system is foreign to many and may be seen as adversarial to patients. This qualitative study explored patients’ perceptions of the IME process.

**Methods**: Patients followed at a regional pain clinic (*Centre Intégré de Santé et de Services Sociaux de l’Abitibi-Témiscamingue)* and scheduled for an IME completed a structured qualitative interview. Questions included: (1) Is this your first IME? (2) What is an IME? (3) Who will conduct it? (4) Why was it requested? (5) What are your expectations?

**Results**: Ten patients (eight men, two women) participated; for eight this was the first IME experience. Five searched online for information about the expert, focusing mainly on negative reviews. Reported comments included: “The expert is paid by the insurer, so he’ll make me go back to work,” “I heard he doesn’t believe in fibromyalgia,” “I don’t know why the insurer doesn’t believe me,” “If he tells me to return to work, I don’t know how I’ll do it,” and “You only see the expert for 15 minutes; I’m afraid someone I don’t know will decide my life.” Only one expected a positive outcome: “The expert will identify my problem.”

**Discussion/Conclusions**: Fear and distrust of the IME process predominated leading to anxiety, and potential reduced engagement in return-to-work effort.

## Risk Factors and Predictive Models of Chronic Cancer-related Pain in Older Adults: Evidence from the Health and Retirement Study

Yangqianxi Wang^1^, Matthew Tang^2^, Annika Bey^1^, Jason Busse^1^, Li Wang^1^

^1^McMaster University, ^2^Queen’s University

**Introduction**: Chronic pain is a prevalent and disabling condition influenced by biological, psychological, and socioenvironmental factors. Among older adults with cancer, chronic cancer-related pain is common and associated with functional impairment and poor quality of life. Understanding and predicting chronic pain risk after cancer diagnosis are essential for targeted interventions. This study developed predictive models to identify risk factors for moderate-to-severe chronic pain among older patients post-cancer diagnosis using generalized linear mixed models (GLMMs) and machine learning based on the Health and Retirement Study (HRS).

**Methods**: Data were from HRS, a nationally representative longitudinal cohort of U.S. adults aged over 45 years. Participants with complete data across three waves—pre-diagnosis, at diagnosis, and post-diagnosis—were included. Temporal changes in demographic, behavioral, and clinical characteristics were analyzed using Pearson’s Chi-squared and Kruskal-Wallis tests. GLMMs evaluated longitudinal associations between cancer diagnosis and pain severity, adjusting for 10 demographic, behavioral, and clinical variables. Four machine learning algorithms—support vector machine, random forest, XGBoost, and neural network—were applied to predict moderate-to-severe pain and identify key predictors.

**Results**: Among 2,272 participants with cancer, 266 (11.7%) consistently reported moderate-to-severe pain. Pain severity differed significantly across time points (p < 0.01), showing temporal variation. Participants with persistent pain had greater ADL limitation (p = 0.001) and lower income (p = 0.035). In GLMMs, higher odds of moderate-to-severe pain were linked to cancer diagnosis (adjusted OR 1.20, 95% CI [1.02-1.42]), multimorbidity (OR 3.55 [2.76-4.55]), ADL limitation (OR 2.08 [1.88-2.30]), and higher BMI (OR 1.05 [1.03-1.07]). Neural network (AUC = 0.733) and XGBoost (AUC = 0.731) achieved the best predictive accuracy.

**Discussion/Conclusions**: Pain severity increased after cancer diagnosis. ADL limitation, multimorbidity, cancer diagnosis, and BMI were consistently associated with moderate-to-severe chronic pain. Integrating statistical and machine learning approaches can improve early identification and personalized management of chronic cancer-related pain in older adults.

## Recovering Together from Pain and Stress After a Stay in the Intensive Care Unit (ICU): A Convergent Mixed Methods Study

Amanda Guerin^1,2^, Andrea Maria Laizner^1,3^, Geneviève Laporte^1,4^, Rosalind Garland^1,4^, Oxana Kapoustina^1^, Céline Gélinas^1,4^

^1^McGill University, ^2^McGill University Health Centre, ^3^Research Institute of McGill University Health Centre, ^4^Jewish General Hospital

**Introduction**: Many patients in the Intensive Care Unit (ICU) experience acute pain and stress, putting them at risk of chronic intensive care-related pain (CIRP). It is estimated that one in three ICU survivors live with CIRP. This study explored how ICU survivors with CIRP and their family caregivers cope with pain and stress.

**Methods**: A convergent mixed-methods study was conducted in 3 university-affiliated ICU settings in Montreal. Quantitative questionnaires and qualitative interviews among survivors who reported CIRP at 3 months post-ICU discharge and their family caregivers were completed. Descriptive statistics for quantitative data and deductive-inductive thematic analysis for qualitative data were performed. Data integration was achieved by building a matrix.

**Results**: A sample of 11 francophone dyads was enrolled. ICU survivors with CIRP were mostly male (n=7; median age=53), admitted for a surgical or trauma diagnosis, half were mechanically ventilated and all received opioids. Most family caregivers were female (n=9; median age=58) and partners (n=8). Survivors reported significant pain (median average pain=4, IQR=3-5; median worst pain=6, IQR=4-7) and memories of ICU severe pain (n=7). Four major themes emerged from interviews: 1) Being Exposed to Pain and Stress, 2) Navigating the Unknown Brings an Abundance of Stressors, 3) Coping Through Recovery Together, and 4) Addressing the Needs of Patients and Family Caregivers. Prioritizing pain management, seeking information, staying active, and family support were valued strategies among dyads, leading to a reframing of acceptance and hope.

**Discussion/Conclusions**: Empowerment of a dyadic approach could improve coping with CIRP in the recovery process.

## “Grey zones and things unsaid”: Patients’ and caregivers’ perceptions of the clinical assessment of chemotherapy-induced peripheral neuropathy in an oncology setting

Angelina Centeno-Báez^1^, Maud Bouffard^2^, Antoine Frasie^3^, Alyson Stone^2^, Maxime Bouchard^4^, Philippe Bérubé-Mercier^1^, Julie Lemieux^5^, Anne Dionne^5^, Jennifer Gewandter^6^, Lucia Gagliese^7^, Lynn Gauthier^1^

^1^Université Laval, ^2^CR-CHU de Québec-Université Laval, ^3^Université de Québec à Trois-Rivières, ^4^Patient Author, ^5^Centre des maladies du sein Deschênes-Fabia, CHU de Québec, Quebec, Canada, ^6^University of Rochester, ^7^York University

**Introduction**: Chemotherapy-induced peripheral neuropathy (CIPN) from taxane- and platinum-based agents is a frequent and disabling complication that remains underassessed in clinical practice. This study explored how people with breast cancer and their caregivers perceive CIPN clinical assessment.

**Methods**: We conducted a secondary, qualitative analysis of semi-structured interviews from a focused ethnography of CIPN-related treatment decisions. Eligible patients were ≥18 years old, cognitively intact, French-speaking, treated with taxanes+/-platinum agents for breast cancer, reporting ≥1 sensory +/- motor/autonomic symptom(s) in the past 24 hours on a 61-item checklist. Eligible caregivers (aged ≥18, cognitively intact, French-speaking) identified as primary support persons were invited to participate. Interviews were analyzed with inductive, reflexive thematic analysis.

**Results**: Patient-participants included 22 French-speaking, middle-aged (median age: 49 [IQR 44-51]) highly educated women (59% postsecondary education). All received taxane-based chemotherapy; 1 also received a platinum agent. Thirteen caregivers participated (76.5%). They were middle-aged (median age: 56 [IQR 49-63]), primarily men (92.3%), partners (92.3%), providing care for 6 months (IQR 5-9). Patients reported a median of 6 checklist symptoms (IQR 4-10; range 1-19), most commonly widespread muscle/joint pain (59.1%), fingertip sensitivity loss (50.0%), and difficulty handling small objects (36.4%). Six themes were identified: CIPN knowledge/understanding; Time-pressured assessment shaped by threshold-based responses, experience reinterpretation, and destabilizing interactions; Assessment process variability; Patient/caregiver initiatives; Barriers; Expectations/needs for improvement.

**Discussion/Conclusions**: Patients’/caregivers’ evolving knowledge provided reference points through which they approached assessment, which was perceived as variable, influenced by brevity of encounters, differing professional approaches, and patient/caregiver initiatives. Findings highlight opportunities to improve clinical practices.

## Retrospective Assessment of Bisphosphonate Therapy Efficacy and Safety in Pediatric Complex Regional Pain Syndrome: A Review of Effectiveness and Side Effects

Christine Lamontagne^1,2,3^, Nicole Fakhory^1^, William Dagg^1,2^, Michelle Nieuwesteeg^2,3^, Heidi Eccles^3^, Leanne Ward^1,2,3^

^1^University of Ottawa, ^2^Children’s Hospital of Eastern Ontario, ^3^CHEO RI

**Introduction**: Complex regional pain syndrome (CRPS) is a chronic, severe pain condition characterized by continuing (spontaneous and/or evoked) regional pain that is disproportionate to the usual course of any known trauma. While bisphosphonates show promise in alleviating adult CRPS, their efficacy and safety in pediatrics remain poorly understood. At CHEO, intravenous (IV) bisphosphonate therapy has been incorporated into pediatric CRPS treatment since 2017.

**Methods**: This retrospective chart review study (1) assessed responder rate to IV bisphosphonate therapy administered every 3 months for 6-12 months in pediatric CRPS patients compared to a control group (based on improvement of Budapest criteria, decrease in pain score by 30% on numerical rating scale [NRS], improvement functioning, or decrease in pain medication), and (2) evaluated the incidence and duration of side effects (including pain flare, nausea/vomiting) and safety events (hypocalcemia or symptomatic hypocalcemia) associated with IV bisphosphonate therapy.

**Results**: A total of 76 patients diagnosed with CRPS were seen at CHEO from 2013-2024 were included (20 treatment and 56 control). Patients that received IV bisphosphonate treatment may improve more quickly than controls, especially in the first 90-180 days, based on physical functioning, NRS pain score, and Budapest criteria. By 1 year, however, improvement in both groups was similar. No major safety events were identified, however, some patients experienced pain flare lasting 3-14 days mainly after the first IV bisphosphonate infusion.

**Discussion/Conclusions**: IV bisphosphonate may be a safe and effective treatment for pediatric CRPS, however, support may be required to manage potential pain flare post infusion.

## The Relationship Between Psychological and Pain Factors in Post-Surgical Patients Enrolled In A Feasibility RCT of a Digital Psychology Program at the Transitional Pain Service at Toronto General Hospital

Alisha Ratnasekera^1,2^, Kristina Axenova^3,4^, Callon Williams^2,3^, Anna Lomanowska^2,3^, Tahir Janmohamed^5^, Molly McCarthy^2,3^, Joel Katz^2,3,4,5,6^, Hance Clarke^2,3,6^, Maxwell Slepian^2,3,6^

^1^Dalla Lana School of Public Health, University of Toronto, ^2^Department of Anesthesia and Pain Management, University Health Network, ^3^Transitional Pain Service, Toronto General Hospital, University Health Network, ^4^Department of Psychology, Faculty of Health, York University, ^5^ManagingLife Inc, ^6^Department of Anesthesiology and Pain Medicine, University of Toronto

**Introduction**: Psychological factors (e.g., anxiety, depression) are known to impact pain, yet access to pain psychologists remains limited. In response, the Transitional Pain Service at Toronto General Hospital developed a digital, self-guided psychology program that teaches pain coping and emotion regulation skills. The current study examines associations between baseline psychological and pain-related factors among post-surgical patients enrolled in a feasibility/limited efficacy RCT of the program (ClinicalTrials.gov ID: NCT06455345).

**Methods**: Pre-randomization baseline data from patients (n = 27; 55.6% female) who underwent surgery <6 months previously were analyzed. Psychological (PROMIS Anxiety, Depression, mental health diagnosis [Yes/No]) and pain-related measures (PROMIS Pain Interference and Prescription Pain Medication Misuse, Pain Intensity, Pain Catastrophizing, Psychological Inflexibility in Pain) were examined using Pearson and point-biserial correlations.

**Results**: Higher anxiety and depression scores were significantly associated with greater pain interference (*r* = .446, *p = .020; r* = .584, *p = .002*), medication misuse (*r* = .549, *p = .004; r* = .742, *p < .001*), pain intensity (*r* = .480, *p = .011; r* = .612, *p < .001*), pain catastrophizing (*r* = .624, *p <.001; r* = .706, *p <.001*), and psychological inflexibility (*r* = .609, *p <.001; r* = .762, *p < .001)*, respectively. Mental health diagnoses (Yes/No) were associated with higher pain interference (*r* = .428, *p = .029)* and psychological inflexibility (*r* = .459, *p = .018)*.

**Discussion/Conclusions**: Psychological factors are strongly associated with post-surgical pain outcomes, highlighting the importance of increasing access to psychological care during the critical postoperative period.

## Prevalence and Predictors of Chronic Post-Surgical Pain After Caesarean Section delivery: A Systematic Review and Meta-Analysis of Observational Studies

Armaanpreet Dhillon^1^, Harjind Kahlon^2^, Jeevan Jeevan Dhillon^3^, Sobindeep Mann^4^, Wenjun Jiang^3^, Andy Cui^3^, Annika Bey^3^, Jason Busse^3^, Li Wang^5^

^1^Western University, ^2^University of Toronto, ^3^McMaster University, ^4^York University, ^5^Department of Anesthesia, McMaster University

**Introduction**: Caesarean sections (C-sections) have been increased by 14% since 1990. Chronic post-surgical pain (CPSP) is a common complication after C-section, associated with reduced quality of life and increased economic burden. However, its prevalence and risk factors remain unclear. This systematic review aimed to assess the prevalence and predictors of CPSP after C-section.

**Methods**: We searched MEDLINE, EMBASE, CINAHL, and PsycINFO up to April for observational studies reporting CPSP (pain ≥3 months) and associated predictors in women who’ve undergone C-sections. Reviewers independently screened studies, assessed risk of bias, and extracted study data in duplicate. We conducted random-effects meta-analysis to pool the CPSP prevalence and the association with each poolable predictor.

**Results**: Thirty-six studies with 36,342 women were eligible; 18 (50%) studies were at high risk of bias. The pooled CPSP prevalence from 33 studies (n=37,179) was 18.0% (95%CI 12-24%). CPSP after C-section was significantly associated with moderate-to-severe acute postoperative pain (OR 1.60 [95%CI 1.19-2.14]) and general anesthesia vs. regional anesthesia (OR 2.22 [95%CI 1.22-4.05]. However, no significant associations were found with age (OR 1.01 at every 10-year decrement [95%CI 0.96-1.06]), BMI (OR 1.04 at every 5-point increment [95%CI 0.92-1.18]), previous C-section (OR 1.20 [95%CI 0.85-1.69]), anxiety (OR 1.05 [95%CI 0.83-1.33]), or depression (OR 0.99 [95%CI 0.71-1.38]).

**Discussion/Conclusions**: Approximately one in five women experiences CPSP after C-section. Moderate-to-severe acute pain and use of general anesthesia are associated with increased risk. Early identification of high-risk individuals and optimizing anesthesia and perioperative pain management may reduce CPSP after C-section.

## “There’s nothing I can do for you. Good luck, have a nice life”: experiences of medical gaslighting in young adults living with chronic pain.

Gabrielle Leblanc-Huard^1^

^1^Université Laval

**Introduction**: Chronic pain is a major public health issue affecting one in four adults. While it is more prevalent among older adults, many young adults (YAs) also live with chronic pain during an important moment of their life, the transition to adulthood, when they are expected to become autonomous and build their identity. For many YAs, chronic pain acts as a biographical rupture, especially when the search for a diagnosis or effective management remains uncertain.

**Aim**: This poster explores experiences of medical gaslighting among YAs living with chronic pain and formulates recommendations to better support them as they navigate life with pain.

**Methods**: The results presented in this poster are drawn from a qualitative and exploratory study. Ten participants aged 25 to 35 living with chronic pain took part in three in-depth narrative interviews. A narrative thematic analysis was conducted to understand each participant’s story in all its complexity.

**Results**: Participants reported multiple forms of medical gaslighting throughout their pain journeys. These experiences affected their sense of self-worth, well-being, and ability to move forward as adults. Many shared that being listened to and met with validation would have profoundly changed their experience.

**Discussion/Conclusions**: In clinical encounters, welcoming and listening to YAs living with chronic pain may take more time, but it can transform how they navigate adulthood and live with pain. Investing time in acknowledging and validating their experiences is not merely an act of empathy. It is a matter of epistemic justice, one that can profoundly reshape their lives.

## Beyond Growing Pains: Patient Perspectives on a Pediatric-to-Adult Chronic Pain Transition Clinic

Christine Lamontagne^1,2,3^, Patricia Poulin^1,4^, Natalie Zur Nedden^5^, Michelle Nieuwesteeg^3^, Heidi Eccles^3^

^1^University Of Ottawa, ^2^CHEO, ^3^CHEORI, ^4^TOH, ^5^OHRI

**Introduction**: Chronic pain decreases quality of life for adolescents and often persists into adulthood. In Ottawa, an interdisciplinary combined adult and pediatric chronic pain clinic between the Children’s hospital of Eastern Ontario and The Ottawa Hospital was established to facilitate the transition of youth to adult pain care using existing resources while minimizing wait time. This study aimed to understand the experience, satisfaction, and recommendation for program improvement from young adults attending the transition clinic.

**Methods**: Online semi-structured interviews, 1-2 hours in duration were conducted. Data analysis was guided by reflexive thematic analysis as outlined by Braun and Clark. Interviews were transcribed verbatim, and coding and analysis of the transcripts was done in duplicate. Reviewers familiarized themselves with the transcripts, then created preliminary coding categories, identified common and recurring themes, refined and named themes through consensus meetings, then proceeded with substantive coding. This was done iteratively until data saturation was reached.

**Results**: Saturation was reached at 11 participants. Emerging themes regarding the transition clinic included feelings of gratefulness, smooth process, change, and uncertainty, however some of the experiences in the adult system were identified as in need of optimization.

**Discussion/Conclusions**: The adult system can be intimidating for youth with chronic pain. This study deepens our understanding of the challenges young adults face during the transition process and can lead to and can lead to opportunities to improve the transition from pediatric to adult pain care.

## Pain and related symptoms in critically ill adults: How are they doing at discharge from the Intensive Care Unit?

Lan-Vy Ho^1,2^, Robin Kagie^1^, Grace Al Hakim^2^, Bachi-Ayukokang Ebob-Anya^1,2^, Geneviève Laporte^1,2^, Céline Gélinas^1,2^

^1^Jewish General Hospital, ^2^McGill University

**Introduction**: Critically ill adults suffer from pain and other symptoms during their stay in the Intensive Care Unit (ICU) but we lack evidence on these symptoms at ICU discharge. This study aimed to describe pain and its related symptoms at ICU discharge.

**Methods**: A correlational descriptive study was conducted in a university affiliated ICU setting in Montreal. ICU patients able to self-report who experienced pain during their ICU stay were eligible. Validated measures were completed by participants at ICU discharge: a) Brief Pain Inventory (BPI), b) Pain Catastrophizing Scale (PCS), and c) modified Edmonton Symptom Assessment Scale (ESAS).

**Results**: A total of 171 participants (25% female; 80% North American, European and Oceanian) with a mean age of 62 years old (SD=13) mainly admitted for a surgical diagnosis (83%) were enrolled. The majority were mechanically ventilated (81%) and received opioids (94% with 72% for >72 hours) with a median ICU length of stay of 4 days (IQR=2-5). At ICU discharge, the medians of average pain and worst pain were 4 (IQR=2-5) and 7 (IQR=4-8), respectively. Pain interference showed the highest medians of 5 with general activity (IQR=2-8), mobilization (IQR=1-8) and deep breathing (IQR=1-7). Pain catastrophizing (median=12; IQR=2-24) correlated positively with average pain (rho=0.32) and worst pain (rho=0.34). Pain correlated moderately (rho=0.42 to 0.54) with anxiety, general discomfort, sadness, tiredness and lack of sleep.

**Discussion/Conclusions**: ICU patients experience high levels of pain and several symptoms at their discharge. Improvement in ICU pain and symptom management is essential to optimize their recovery.

## Elucidating the CIPN-related treatment change decision-making process: Preliminary analysis of a focused ethnography

Maud Bouffard^1^, Antoine Frasie^1,2^, Angelina Centeno Baez^1, 3^, Philippe Bérubé-Mercier^1^, Maxime Bouchard^4^, Alyson Stone^1^, Julie Lemieux^1, 5^, Anne Dionne^1, 5, 6^, Jennifer Gewandter^7^, Lucia Gagliese^8^, Lynn Gauthier^1, 9^

^1^CHU de Québec-Université Laval Research Center, Oncology Division, Quebec, Canada, ^2^Department of Anatomy, Program of physiotherapy, Université du Québec à Trois-Rivières, Quebec, Canada, ^3^Department of Medicine, Faculty of Medicine, Université Laval, Quebec, Canada, ^4^Patient Author, ^5^Centre des maladies du sein Deschênes-Fabia, CHU de Québec, Quebec, Canada, ^6^Faculty of Pharmacy, Université Laval, Quebec, Canada, ^7^Department of Anesthesiology and Perioperative Medicine, University of Rochester Medical Center School of Medicine and Dentistry, Rochester, United States of America., ^8^York University, School of Kinesiology and Health Science, Toronto, Ontario, Canada., ^9^Department of Family and Emergency Medicine, Faculty of Medicine, Université Laval, Quebec, Canada

**Introduction**: Chemotherapy-induced peripheral neuropathy (CIPN), characterized by sensorimotor symptoms (numbness, pain, balance loss), affects 43%-80% of people receiving taxane-based chemotherapy for breast cancer. In 11.5%-38% of people, despite unclear survival impact, management often involves chemotherapy modifications (treatment delays [TD], dose reductions [DR], premature discontinuation [PD]). Minimal decision-making guidance underscores the need to examine real-world clinical processes.

**Methods**: A focused ethnography was conducted in a cancer center with 22 patients receiving taxanes±platinum agents for stages I-III breast cancer, reporting ≥1 sensory±motor/autonomic symptom(s) on a 61-item checklist, 13 caregivers, and 40 treating healthcare providers. Data include observation field notes of infusion room and clinical discussions and electronic medical records. Grounded theory analyses employed handwritten and Cmap Tools concept maps for 6 patients (mean age 48±7.8 years, range: 35-51), selected for this preliminary analysis based on maximal variation in CIPN-related treatment decisions.

**Results**: Patients selected 10.7±4.3 [5-19] checklist items. We conducted 41 observations (mean 996.7±823.4 mins [40-1,965 mins]), with 6.8±5.0 [1-14] observations/patient. One patient had no treatment change, one had 1TD, one had 1DR, one had 1TD+1DR, one had PD, and one had 2TDs+2DRs+1PD. Iterative analyses revealed interrelationships among 3 dimensions contributing to CIPN-related decision-making: 1) environment/emotional climate (clinical team inconsistency, patients’ need to be heard/understood); 2) communication/documentation/action about symptom onset, progression, functional impact; and 3) chemotherapy choices to manage CIPN (continuation+surveillance, TD vs. DR).

**Discussion/Conclusions**: This study’s detailed insights into CIPN-related treatment discussions will guide future research on the development of clinically grounded CIPN decision-support tools.

## Chronic intensive-care related pain (CIRP) and opioid use in survivors: Profile and associated factors at 3 months post-discharge

Bachi-Ayukokang Ebob-Anya^1,2^, Robin Kagie^2^, Geneviève Laporte^1,2^, Michael Goldfarb^1,2^, Marc O. Martel^1^, Alice Wagenaar^3^, Céline Gélinas^1,2^

^1^McGill University, ^2^Jewish General Hospital, ^3^Université du Québec à Trois-Rivières

**Introduction**: Critically ill adults are exposed to high levels of pain during their stay in the Intensive Care Unit (ICU) for which they receive opioids. To describe the profile and factors associated with chronic intensive-care related pain (CIRP) and opioid use/misuse in survivors at 3 months post-ICU discharge.

**Methods**: A prospective cohort study was conducted in five Quebec ICU settings. Eligible patients were those who reported ICU-related pain and worst pain ≥ 3 at 3 months. The Brief Pain Inventory (BPI) measured pain intensity and pain interference. Opioid misuse was measured with the Opioid Compliance Checklist (OCC).

**Results**: At 3 months, 122/293(42%) presented with CIRP. Majority were male (65%) and North American (73%) with a mean (SD) age of 60 (14) years, were admitted for a surgical diagnosis (52%) and mechanically ventilated (59%). Pain interference at 3 months showed highest medians of 4 and 5 for general activity, mood, walking and sleep. Worst pain intensity at 3 months was higher in non-ventilated versus ventilated patients (median=7 vs 5, U=1212.5, p=0.01). Almost one in three survivors with CIRP used opioids daily at 3 months (n=37, 30%) and 24% misused opioids (e.g., prescribed opioids from > 1 provider). Worst pain was greater for opioid users than non-users (median=7 vs 5, U=887, p=<0.001). Mechanical ventilation (x^2^=7.165, p=0.007) and surgical/trauma diagnosis (x^2^=6.883, p=0.032) were associated with opioid use.

**Discussion/Conclusions**: A high proportion of ICU survivors live with CIRP and use opioids post-discharge. Chronic pain prevention should be prioritized in this vulnerable population.

## Exploring the effect of prior caregiving activities on maternal physiological reactivity during skin-to-skin contact for infant pain in the Neonatal Intensive Care Unit

Nichaela Garvey^1^, Vadim Tyuryaev^1^, Vibhuti Shah^2^, Rebecca Pillai Riddell^1^

^1^York University, ^2^Mount Sinai Hospital

**Introduction**: Holding a preterm infant in skin-to-skin contact during a painful procedure (SSCP) can be a distressing experience for mothers. The current study aimed to explore the effect of previous caregiving activities in the neonatal intensive care unit (NICU) on maternal pain-related physiological distress during SSCP.

**Methods**: The study included 22 mother-infant dyads at a tertiary NICU who participated in a SSCP study. Before SSCP, caregivers self-reported their weekly average amount of minutes spent on direct caregiving activities. During SSCP, maternal heart rate (HR) was collected for six 30-second epochs surrounding lance using electrocardiography (3 prior to lance and 3 post lance).

**Results**: Three linear mixed models assessed the differential effect of caregiving activities on maternal HR. Across lance, skin-to-skin was not associated with maternal HR (*F*(5,100) = 0.62, *p* = 0.69). A significant interaction was found for handling (*F*(5,100) = 2.32, *p* = 0.04), indicating variation across epochs. A stronger interaction emerged for hand hugging (*F*(5,100) = 4.50, *p* < 0.01). Post-hoc analysis revealed that the decrease in effect between Epoch 1 and Epoch 5 and Epoch 1 and Epoch 6 was significant, as the slope in Epoch 1 was significantly steeper than in Epoch 5 (*t*(100) = 3.36, p = 0.01) and Epoch 6 (*t*(100) = 2.95, p = 0.04).

**Discussion/Conclusions**: Previous handling and hand hugging were associated with maternal HR from 30-90 seconds before lance, supporting an anticipatory physiological distress mechanism during SSCP. Skin-to-skin was not associated with maternal HR in this context, warranting further investigation.

## Maternal Recommendations on Supporting Skin-to-Skin Contact for Neonatal Pain in the NICU: A Qualitative Analysis

Haleh Hashemi^1^, Estreya Cohen^1^, Nichaela Garvey^1^, Andrea Lebovic^1^, Fabiana Bacchini^2^, Lesley Johannsson^3^, Carol Cheng^3^, Vibhuti Shah^3^, Rebecca Pillai Riddell^1^

^1^York University, ^2^Canadian Premature Babies Foundation, ^3^Mount Sinai Hospital

**Introduction**: Skin-to-skin contact for procedural pain (SSCP) are recognized for their physiological and emotional benefits in the neonatal intensive care unit (NICU), including pain reduction in preterm infants. However, little is known about how birthing parents of very and extremely preterm infants (V/EPT; < 32 weeks gestational age), a significantly more challenging preterm infant population to enact SSCP, perceive this intervention. This study aimed to explore birthing parents’ recommendations on supporting SSCP in the NICU with their V/EPT infants.

**Methods**: In partnership with a national preterm parent organization, virtual interviews were conducted with 38 mothers of V/EPT infants from across Canada, who had been admitted to the NICU within the past five years. Transcripts were subsequently analyzed using 6 phases of thematic analysis.

**Results**: Data was synthesized around themes of 1) Experience with SSCP, 2) Encouragement for SSCP from others in the NICU 3) Future suggestions to support SSCP. In addition, mothers’ opinions about *a priori* concepts and potential interventions (generated from pilot data) were also vetted. Important actionable facilitators and interventions to support SSCP with parents of V/EPT infants were discerned.

**Discussion/Conclusions**: Although most found their experience rewarding, barriers such as limited instruction, inconsistent staff support, procedural challenges, and emotional strain often hindered use of SSCP. Enhancing staff training, standardizing protocols, offering mental health support, and adopting flexible, family-centered policies appear key to improving SSCP engagement with the youngest preterm infants. Improving SSCP information and support can better enable parents to support preterm infants, improving outcomes for both.

## Persistent Headaches and Olfactory Dysfunctions in Patients with Long COVID

Laurianne Thompson^1^, Annie Sylfra^1,2^, Majd Balbous^1,2^, Frank Cloutier^1^, Johannes Frasnelli^1, 3, 4^

^1^Department of Anatomy, Université du Québec à Trois-Rivières (UQTR), ^2^Faculté de Médecine, Université de Montréal (UdeM), ^3^Research Center, Hôpital du Sacré-Coeur de Montréal, ^4^Centre de recherche de l’Institut universitaire en gériatrie de Montréal

**Aim**: The study aims to determine the association between headaches and olfactory dysfunctions in patients with long COVID.

**Methods**: Patients who had previously tested positive for COVID-19 were recruited online or through posters to obtain a total of 70 participants. After providing electronic consent, participants were mailed the University of Pennsylvania Smell Identification Test (UPSIT) to assess their olfactory function, after which an online appointment was scheduled. During this appointment, participants completed four questionnaires: a sociodemographic questionnaire, the Headache Impact Test-6 (HIT-6) assessing the impact of headaches on daily life, the Hospital Anxiety and Depression Scale (HADS) measuring anxiety and depression, and the Questionnaire of Olfactory Disorders (QOD) evaluating the impact of smell loss and headaches on quality of life.

**Results**: Preliminary analyses (n = 20) revealed no significant correlation between olfactory dysfunction and the presence of headaches (r = -.43, 95% CI [-.74, .12], p = .06). However, participants who experienced headaches showed significantly higher HADS scores, indicating greater anxiety and depression (mean difference: 6.1 [95% CI: 2.6 - 10.2]; t(18) = 3.0, p = .02, d = 1.0).

**Discussion/Conclusions**: According to the preliminary results, severe olfactory dysfunction does not appear to be indicative of the presence or intensity of headaches. However, the presence of headaches is significantly correlated with increased levels of anxiety and depression among individuals.

## More than growing pains: Assessing the Impact of an Interdisciplinary Pediatric-Adult Chronic Pain Clinic on Transition to Adult pain care

Christine Lamontagne^1,2,3^, Alice Kim^1^, Daniel McIsaac^1,4,5^, Daniel James^1,4^, Heidi Eccles^3^, Michelle Nieuwesteeg^2,3^

^1^University of Ottawa, ^2^CHEO, ^3^CHEORI, ^4^TOH, ^5^OHRI

**Introduction**: Chronic pain significantly affects adolescents’ functioning and quality of life, and up to half continue to experience pain into adulthood. A coordinated transition to adult services is essential, yet long waitlists and system complexity often leave youth without timely support. To address this gap, the Children’s Hospital of Eastern Ontario (CHEO) and The Ottawa Hospital (TOH) established a joint interdisciplinary transition clinic to streamline the shift from pediatric to adult chronic pain care using existing resources and reducing wait times.

**Methods**: This retrospective chart review evaluated the clinic’s impact on health care utilization. 94 patients were identified from the electronic health record: 47 youth seen in the transition clinic between 2019 and 2023 and 47 matched controls from the pre-transition period (2016-2019). Matching criteria included age, sex, diagnosis and pain intensity. Variables extracted were demographic data (age, sex), diagnosis, pain intensity, emergency department visits, hospitalizations, and specialist referrals.

**Results**: The sample of 94 patients included 74 females and 20 males. Control and transition patients had comparable numbers of ED visits and hospitalizations, however, 45% of transition-clinic patients attended 1 or more specialist referrals vs only 15% of control patients, possibly indicating greater uptake of recommended follow-up care.

**Discussion/Conclusions**: Youth seen in the transition clinic were more likely to access specialist services, suggesting improved continuity of care compared with the pre-transition cohort, who may have faced barriers such as limited primary care attachment or long adult-care waitlists. Ottawa’s joint pediatric-adult chronic pain transition model remains one of the few such programs in Canada and offers a promising approach to reducing care gaps for transitioning youth

## The Impact of Cancer Diagnosis on Pain Burden Among Older Adults: A Mixed-Effects Difference-in-Differences (DiD) Model

Yangqianxi Wang^1^, Matthew Tang^2^, Annika Bey^1^, Jason Busse^1^, Li Wang^1^

^1^Mcmaster University, ^2^Queen’s University

**Introduction**: Pain is a common and disabling condition affecting nearly one in five adults globally, with higher prevalence among older adults. Pain is also most distressing symptom in cancer patients, contributing to functional decline and reduced quality of life. Yet, longitudinal evidence separating the impact of cancer diagnosis on pain from aging and comorbidities is limited. This study applied a Difference-in-Differences (DiD) framework to estimate the causal effect of cancer diagnosis on moderate-to- severe pain using data from the Health and Retirement Study (HRS).

**Methods**: Data were drawn from HRS, a nationally representative cohort of U.S. adults aged ≥60 years. Participants with complete data across three waves—pre-diagnosis, at diagnosis, and post-diagnosis—were included. Mixed-effects DiD models with individual random intercepts estimated within-person changes in pain severity before and after cancer diagnosis relative to non-cancer controls. Ten demographic, behavioral, and clinical covariates were adjusted to account for confounding.

**Results**: Of 20,404 participants, 2,272 were diagnosed with cancer and had 3-wave complete longitudinal data; 266 (11.7%) consistently reported moderate-to-severe pain. In the unadjusted model, a significant cancer-by-post interaction (OR = 1.20, 95% CI [1.02-1.41]) indicated greater pain increases among cancer participants. After adjusting10 pre-defined confounders, cancer diagnosis remained significantly associated with increased pain risk (adjusted OR = 1.29, 95% CI [1.09-1.52]), representing a 29% increase in odds of developing moderate-to-severe pain compared to non-cancer participants. Younger age, lower education, smoking, higher BMI, and ADL limitation were associated with greater pain risk.

**Discussion/Conclusions**: Pain severity significantly worsened following cancer diagnosis compared to non-cancer participants. These findings support a causal link and highlighted the need for early pain monitoring and target interventions in survivorship care for older adults with cancer.

## “Immigration to oneself”: Participatory co-creation of an information resource by and for immigrant people living with chronic pain

Lise Dassieu^1,2,3^, Jonathan Bichon^1^, Maripier Arcand-Langlois^3^, Estelle Carde^3,4^, Anaïs Lacasse^3,5^, Jacqueline Schneider^1,6^

^1^Research Centre of the CIUSSS du Nord-de-l’Ile-de-Montréal, ^2^School of Social Work, Université du Québec à Montréal, ^3^Quebec Pain Research Network, ^4^Department of Sociology, Université de Montréal, ^5^Department of Health Sciences, Université du Québec en Abitibi-Témiscamingue, ^6^Department of Anthropology, Université de Montréal

**Introduction**: Chronic pain is frequent among immigrants in Canada, but they often lack tailored resources to navigate the healthcare system and self-manage their pain. This qualitative participatory study aimed to co-create, with a group of immigrant people living with chronic pain, an information resource to better support immigrants in their healthcare and pain management experiences.

**Methods**: A group of six immigrant women with chronic pain met regularly during 10 online discussion sessions (August-November 2025). Participants were recruited in community-based organizations in Quebec. They came from the Americas, Africa, and the Middle-East, and were aged 33-65 years. They had lived in Quebec (regions of Montreal, Laval, Montérégie) for between 2 and 30 years. Several chronic pain conditions were represented (fibromyalgia, migraine, rheumatoid arthritis, endometriosis, etc.).

**Results**: Participants shared their lived experiences and knowledge on the following themes: (1) navigating pain care services; (2) daily life with chronic pain; (3) links between migration and chronic pain. Their discussions formed the basis for the co-development of a guidebook and a series of animated infographics bringing together the knowledge and advice they wished to share with others facing similar challenges. These resources will be translated into different languages and distributed to organizations specializing in immigration and pain.

**Discussion/Conclusions**: This participatory research based on a co-creation approach has the potential to catalyze social change and improve the experiences of immigrant people living with chronic pain. The participatory process fostered mutual support between participants and valued the knowledge acquired during their trajectories with immigration and pain.

## Telescoping and Phantom Limb Pain: Clinical and Psychosocial Correlates

Andrea Aternali^1^, Heather Lumsden-Ruegg^1^, Lora Appel^2^, Sander L. Hitzig^3^, Amanda L. Mayo^4^, Joel Katz^1^

^1^Department of Psychology, York University, ^2^School of Health Policy & Management, York University, ^3^St. John’s Rehab Research Program, Sunnybrook Research Institute, ^4^Physical Medicine & Rehabilitation, Sunnybrook Health Sciences Centre

**Introduction**: Phantom limb pain (PLP) is a common and distressing consequence of limb loss, with significant impacts on daily functioning. Some also experience phantom sensations such as telescoping, the perceived shortening of the phantom limb. Whether telescoping is linked to PLP or broader psychosocial outcomes remains unclear.

**Methods**: This study examined whether individuals with and without telescoping differ on PLP intensity and psychosocial variables. Adults living with limb loss for at least three months completed online questionnaires assessing demographics, PLP intensity (0-10 scale), and telescoping (yes/no; percent). Participants also completed measures of pain interference (BPI), neuropathic pain (IDPQ-6), pain catastrophizing (PCS-4), depression and anxiety (PHQ-4), optimism (LOT-R), resilience (CD-RISC2), and acceptance (CPAQ-8). Independent-samples *t* tests, Chi-square tests, and Pearson correlations were conducted.

**Results**: Fifty-one individuals with limb loss (33 male; M_age_ = 49.5 ± 15.4 years) participated. Twenty-three (45.1%) reported telescoping and 28 (54.9%) did not. Those reporting telescoping were significantly younger and more likely to have (1) a right upper-limb, (2) below-elbow loss, and (3) higher symptoms of depression and anxiety (all *p*s < .05). Group differences were not found for PLP intensity or other variables. Greater percent telescoping was associated with lower pain interference, *r*(21) = -.431, *p* = .040, and lower PLP intensity, *r*(21) = -.490, *p* = .018.

**Discussion/Conclusions**: Telescoping is not a stand-alone marker of lower pain burden. It may reflect both affective distress (e.g., anxiety/depression) for some, while representing adaptive adjustment for others. Routine screening for anxiety and depression may support tailored interventions for individuals who experience telescoping.

## Treatment/management/ pain programs

### Le traitement, la gestion ou les programmes de prise en charge de la douleur

## Adapting the Pediatric Fear-Avoidance Model of Chronic Pain for Autism Spectrum Disorder: A Case Series

Abirami Kandasamy^1^, Joshua Lee^1^, Fatima Di Valentin^1^, Adam Newton^1^

^1^Children’s Hospital, London Health Sciences Centre

**Introduction**: In the Pediatric Fear-Avoidance (PFA) Model, pain-related fear drives avoidance and disability, and recovery occurs when fear diminishes sufficiently to permit confrontation of avoided activities. For adolescents with Autism Spectrum Disorder (ASD), characteristics such as sensory hyperreactivity, cognitive rigidity, and intolerance of uncertainty may amplify fear responses and reinforce rigid protective behaviours. These behaviours can persist despite reduction or resolution of pain, prolonging functional impairment. Traditional interventions that emphasize insight and cognitive restructuring may be less effective for some youth with ASD, particularly when rigidity and concrete thinking limit cognitive flexibility. We propose adapting the PFA model so that structured behavioural confrontation precedes fear reduction, positioning graded exposure as the primary mechanism facilitating functional recovery.

**Methods**: Four adolescents (ages 13-17) with verbal ASD and chronic pain presentations, were referred following prolonged avoidance of the affected limb (≥12 months) and having previously received Cognitive Behavioural Therapy, without meaningful reduction in pain-related fear. At intake, patients demonstrated severe functional impairment, deconditioning, and entrenched avoidance patterns. Each adolescent completed an interdisciplinary intervention guided by an adapted PFA framework. Treatment included medication management, coordinated physiotherapy and psychology interventions using Behavioural Therapy principles, and caregiver coaching to practice exposures with reinforcement schedules at home.

**Results**: All adolescents reported reductions in pain-related fear, tolerated contact with the affected body region, and engaged in previously avoided activities.

**Discussion/Conclusions**: Emphasizing confrontation as the driver of fear reduction may be the key to facilitating functional recovery, even after prolonged avoidance, for adolescents with ASD and chronic pain.

## Facteurs influençant les croyances et l’adaptation des professionnelles et des professionnels de la santé au sein des cliniques interdisciplinaires de première ligne en douleur chronique

Nick-Kevin Jérôme^1,2^, Tania Augière^1^, Gabriella Lavoie-Dias^1,2^, Manon Choinière^1,3^, Yves Couturier^4,5^, Gabrielle Pagé^1,2,3^

^1^Centre de recherche du Centre hospitalier de l’Université de Montréal (CRCHUM), ^2^Département de psychologie, Université de Montréal, ^3^Département d’anesthésiologie et médecine de la douleur, Université de Montréal, ^4^Centre recherche du Centre hospitalier de l’Université de Sherbrooke (CRCHUS), ^5^Département de travail social, Université de Sherbrooke

**Aim**: En 2021, le ministère de la Santé et des Services sociaux du Québec a lancé un Plan d’action afin d’assurer une meilleure coordination des services et une gestion optimale de la douleur chronique. Des cliniques interdisciplinaires ont ainsi vu le jour en première ligne. Cette étude vise à identifier les facteurs influençant la collaboration des clinicien·nes de ces équipes et les croyances envers la patientèle vivant de la douleur chronique.

**Méthodologie**: Les clinicien·nes (n=27) de cinq cliniques interdisciplinaires participantes ont rempli un questionnaire dans les six premiers mois suivant l’implantation des nouveaux services. Des modèles de régressions linéaires ont été utilisés pour examiner les associations entre **1)** le nombre d’années d’expériences (Expérience), **2)** la perception du clinicien ou de la clinicienne de ses compétences en gestion de la douleur (Compétence) et **3)** jusqu’à quel point la gestion de la douleur chronique représente un défi (Défi) avec la difficulté perçue de satisfaire la patientèle dans leur besoin de soulagement de la douleur (Soulagement) (modèle 1), la perception que la patientèle surestime l’impact qu’a la douleur sur leur quotidien (Impact) (modèle 2; Expérience et Compétence seulement comme variables indépendantes) et l’importance de la collaboration interdisciplinaire (Collaboration) (modèle 3).

**Résultat**: Les résultats indiquent que plus la variable Défi était élevée, plus les personnes cliniciennes croyaient difficile de soulager la douleur de la patientèle, et ce, même en contrôlant pour l’Expérience et la Compétence (p=0,04; ẞ=0,44). Aucune autre variable n’était associée à l’Impact ou au Soulagement (p>0,05).

**Discussion/Conclusions**: Ces résultats soulignent le besoin important de ressources en douleur chronique pour soutenir les clinicien·nes afin de pallier à certaines croyances à propos des patient·es vivant avec ce problème.

## User Experiences with Solace, an AI Chatbot for Managing Chronic Pain: A Qualitative Content Analysis of Conversation Transcripts and Self-Report Assessments

Anna M. Lomanowska^1^, Eileen Liang^1^, Veronika Kolarska^1^, Heather Lumsden-Ruegg^2^, Stephanie Buryk-Iggers^1^, Binh Nguyen^3^, Tahir Janmohamed^3^, Hance Clarke^1,4,5^, Joel Katz^1,2,4,5^, Nils G. Niederstrasser^6^, P. Maxwell Slepian^1,3,4,5^

^1^Transitional Pain Service, Department of Anesthesia and Pain Management, Toronto General Hospital, University Health Network, Toronto, ON, ^2^Department of Psychology, York University, Toronto, ON, ^3^ManagingLife, Inc., Toronto, ON, ^4^Department of Anesthesiology and Pain Medicine, University of Toronto, Toronto, ON, ^5^University of Toronto Centre for the Study of Pain, University of Toronto, Toronto, ON, ^6^School of Psychology, Sport, and Health Sciences, University of Portsmouth, UK

**Introduction**: Conversational agents powered by generative artificial intelligence (AI) have garnered substantial interest for their potential to increase access to on-demand psychology interventions. We recently developed a first-of-its-kind, expert trained AI-driven chatbot, Solace, to deliver evidence‑based pain psychology strategies for chronic pain management. We aimed to evaluate users’ experience with Solace by examining conversation transcripts and self-reported assessments of interactions with the chatbot.

**Methods**: Adults with chronic pain (N=175) recruited from the online platform, Prolific, interacted with Solace for ≥25 minutes. Transcripts of participants’ conversations with Solace and responses to four free-text questions regarding the interaction (likes, dislikes, recommendations, anything else to share) were analyzed by two coders using qualitative content analysis.

**Results**: Directed content analysis of transcripts demonstrated that Solace effectively employed a conversational approach aligned with Motivational Interviewing and evidence-supported therapeutic relationships. Pain management strategies recommended by Solace reflected evidence-based psychology approaches, including relaxation, psychoeducation, graded progression, self-compassion, and mindfulness, as well as gentle movement and physiotherapy techniques. Solace appropriately steered the conversation away from topics that are outside of its safety guardrails. Inductive content analysis of participants’ open-ended responses revealed four main themes capturing both strengths and areas for improvement: attributes of Solace as an agent, conversation flow, quality of information provided, and digital platform functionality.

**Discussion/Conclusions**: This in‑depth evaluation demonstrates that Solace effectively delivers evidence-based psychology content in interactions with users. Participants provided a rich assessment of Solace’s features, informing its further refinement as the first available evidence-based AI-driven tool for chronic pain support.

## Integrated Management of Chronic Pain and Post-Traumatic Stress Disorder: The Role of Stellate Ganglion Block and a Novel Care Pathway in a Tertiary Pain Clinic

Etienne J Bisson^1,2^, Shelby Lee^1^, Paul Hook^3,4^, Sean McEvoy^3^, Christopher Haley^1,2^, David Clinkard^1,2^, Tim Salomons^1,5^, Yuliya Knyahnytska^6,7^, Ian Gilron^1,2^, Scott Duggan^1,2^

^1^Chronic Pain Clinic, Kingston Health Sciences Centre, Kingston, Canada, ^2^Department of Anesthesiology & Perioperative Medicine, Queen’s University, Kingston, Canada, ^3^Person with Lived Experience, Kingston, Canada, ^4^Canadian Institute for Military and Veteran Health, Queen’s University, Kingston, Canada, ^5^Department of Psychology, Queen’s University, Kingston, Canada, ^6^Department of Psychiatry, Queen’s University, Kingston, Canada, ^7^Interventional Psychiatry Department, Providence Care Centre, Kingston, Canada

**Introduction**: Chronic pain (CP) and post-traumatic stress disorder (PTSD) often co-occur, complicating treatment and highlighting the need for integrated approaches. Stellate Ganglion Block (SGB), originally used for upper extremity pain has shown potential efficacy in managing PTSD. Hence, we aimed to develop and evaluate the benefit of a novel care pathway to support Canadian Armed Forces (CAF), RCMP members, Veterans, and first responders with PTSD and CP.

**Methods**: Adult CAF/RCMP members, Veterans, and first responders with PTSD and CP were enrolled in a care pathway involving three ultrasound‑guided SGB administered on the ride side, 60 days apart. Procedures followed established clinical guidelines and included continuous monitoring. Participants completed assessments before each injection, bi‑weekly for six weeks afterward, and again two months following the final treatment. Preliminary descriptive analyses were performed to evaluate the benefits of the program. Ethics approval was obtained for this work in the setting of a quality improvement project.

**Results**: Twelve patients were enrolled from July 2025 to February 2026 with nine completing the first SGB round of data collection (all male, mean(SD) age= 46(11)). Based on patients’ impressions of change, 75% reported improvement in PTSD symptoms, and 62.5% in pain symptoms 2 weeks after one SGB treatment and were maintained 2 months later. Results from patient-reported outcomes and follow-up treatments will be presented.

**Discussion/Conclusions**: Patients with PTSD and CP undergoing the first of three treatments tolerated the procedure well, with most reporting improvement. These preliminary findings are encouraging as we continue to evaluate this novel pathway.

## Fluoroscopy-Guided High-Intensity Focused Ultrasound for Sacroiliac Joint Pain: A New Method for an Old Problem

Kevin Smith^1^, Jamie Smith^1^, Lynn Kohan^2^, Michael Gofeld^1^

^1^ Unika Medical Centre, ^2^University of Virginia

**Introduction**: This retrospective analysis summarizes the first 12 months of clinical use of the Neurolyser XR fluoroscopy-guided high-intensity focused ultrasound (HIFU) for the treatment of SIJ-mediated pain.

**Methods**: 38 patients received ablative HIFU of the L5 to S3 branches using the FUSMobile Neurolyzer XR. SIJ-mediated pain was confirmed through diagnostic SIJ injections or diagnostic L5 dorsal ramus and S1 to S3 lateral branch blocks. Patient demographics and procedural characteristics were collected. Clinical outcome data is still in process.

**Results:**

Mean age: 72 (48-93)

Gender: 19 male, 19 female

Number of sites: mean 9 (3-18)

Duration: mean 25.3 min (14-46 min)

Fluoroscopy dose (DAP, Gy x cm2): mean 2.04 (1.01-4.08)

Procedural pain: mean 6.3/10 (0-10)

The majority of patients reported a meaningful immediate reduction in SIJ pain.

**Discussion/Conclusions**: As the first approved North American site to use the Neurolyzer XR in clinical practice, we have the unique opportunity to pioneer its implementation in routine patient care. Recognizing the benefits of future clinical studies on the use of HIFU for SIJ-mediated pain, the following insights are offered based on our 12-month experience:

- HIFU ablation of the sacral lateral branches is typically more painful than HIFU neurotomy of the lumbar medial branches;

HIFU procedure is faster than RFA;

HIFU is possibly more efficacious with a strip lesion;

Increasing cycle duration from 50 seconds to 90 seconds was the most effective analgesic strategy;

Consider a reduction in Joules delivered (700-1000 total) to improve patient procedural satisfaction.

## Development of a virtual reality content screening tool for Recreation Therapists working in long-term care

Susan Tupper^1^, Rebecca Genoe^2^, Karen Kindrachuk^1^, Princess Adedokun^2^, Taylor Teckchandani^2^

^1^Saskatchewan Health Authority, ^2^University of Regina

**Introduction**: Recreation Therapists (RecTs) provide meaningful leisure opportunities for long-term care (LTC) residents, addressing physical, social, spiritual, emotional and cognitive well-being through person-centered, strengths-based approaches. Virtual reality (VR) is used by RecTs to improve residents’ chronic pain, physical activity participation, mood, and cognitive health. Currently, RecTs do not use a standardized approach to screen VR content for appropriateness to address residents’ therapeutic needs. We co-designed a VR content screening tool to support criteria-based content selection and optimize resident outcomes.

**Methods**: Based on results of a scoping review (n=26 articles reviewed), tool criteria were selected by a working-group of n=6 LTC-based RecTs. RecTs from 6 provinces evaluated the tool prototype with the System Usability Scale (SUS) and provided qualitative feedback through an online survey (n=29 participants). An online nominal group process was held with n=7 people with expertise in LTC and VR to finalize the tool.

**Results**: Six screening categories were included in the initial draft: game/app information (e.g. compatible headsets); user interaction (e.g. position); content type and accessibility (e.g. genre); recommended abilities (e.g. physical demand); potential outcomes (e.g. reduced discomfort); safety considerations (e.g. sudden movements). Survey participants rated the prototype 75/100 (range=42-100) with the SUS. Eleven improvement recommendations were identified with content analysis. Two online nominal group meetings resolved revision recommendations through discussion and consensus voting.

**Discussion/Conclusions**: The new VR content screening tool was developed and iteratively refined with broad feedback to support VR content selection for LTC home residents. Further research is required to examine tool reliability and utility.

## The MusKiP Trial: Preliminary Quantitative Results of Music as an Adjunct to Intravenous Ketamine for Chronic Noncancer Pain

Carlos Gevers-Montoro^1,2^, Louisia Starnino^3^, Amanda Gisondi^3^, Sylvie Toupin^3^, Fatiha Benrahmani^1^, Dany Bouchard^4^, Alexandre Lehmann^5^, Kyle Greenway^6^, Jonathan Hudon^3^, Mark Ware^3^, Mathieu Roy^1,2^

^1^Psychology Department, McGill University, Montreal, Qc, ^2^Alan Edwards Centre for Research on Pain, McGill University, Montreal, Qc, ^3^Alan Edwards Pain Management Unit, McGill University Health Centre, Montreal, Qc, ^4^Montreal General Hospital, McGill University Health Centre, Montreal, Qc, ^5^Centre for Research on Brain, Language, and Music, McGill University, Montreal, Qc, ^6^Jewish General Hospital, McGill University Health Centre, Montreal, Qc

**Introduction**: Ketamine is an emerging treatment for chronic pain, yet the durability of its effects remains insufficiently explored. The clinical context during ketamine therapy, including music, may shape treatment experience and outcomes. This study examines whether different music contexts during intravenous ketamine infusions influence immediate and longer-term pain outcomes in patients with chronic non-cancer pain.

**Methods**: We are conducting a randomized crossover trial at the Alan Edwards Pain Management Unit (McGill University Health Centre). Fifteen patients receiving clinically indicated ketamine infusions (0.5 mg/kg, 60 minutes) underwent four sessions at 5-week intervals: usual care followed by three experimental conditions in randomized order (silence, therapist-selected music, preferred music). Pain intensity and unpleasantness (0-10 NRS) were assessed pre- and post-infusion and longitudinally over 5 weeks using multilevel modelling. Baseline psychological characteristics were explored as moderators.

**Results**: Acutely, ketamine produced robust hypoalgesia across conditions (mean reduction: intensity 2.63 points; unpleasantness 2.95 points; both *p*<0.001), with no evidence of between-condition differences (*p*>0.19). Longitudinal analyses revealed significant time×condition interactions for pain intensity (*p*=0.003) and unpleasantness (*p*=0.012). Preferred music yielded more sustained pain reductions for up to 5 weeks compared with usual care and therapist-selected music. Psychological flexibility moderated longitudinal outcomes, partly accounting for differences observed with preferred music.

**Discussion/Conclusions**: Music context did not affect acute hypoalgesia but influenced the persistence of pain relief following ketamine infusions. Self-selected music was associated with more durable pain reductions, particularly among individuals with greater psychological flexibility. These findings underscore the clinical importance of contextual and personalized approaches in pain management.

## MusKiP: Music and Ketamine in Chronic Noncancer Pain — A Preliminary Qualitative Study

Louisia Starnino^1^, Carlos Gevers-Montoro^2^, Amanda Gisondi^1^, Alexandra Therond^3^, Sylvie Toupin^1^, Dany Bouchard^4^, Alexandre Lehmann^5^, Kyle Greenway^6^, Hudon Jonathan^1^, Mark Ware^7^, Mathieu Roy^8^

^1^Alan Edwards Pain Management Unit, McGill University Health Centre, ^2^Alan Edwards Centre For Research on Pain, McGill University, ^3^Université du Québec à Montréal, ^4^McGill University Health Centre, ^5^Centre for Research on Brain, Language, and Music, McGill University, ^6^Jewish General Hospital, McGill University, ^7^McGill University, ^8^Alan Edwards Research Centre for Pain, McGill University

**Introduction**: Intravenous (IV) ketamine is increasingly used for chronic noncancer pain, yet patient experiences vary widely. Beyond analgesia, ketamine produces alterations in sensory, emotional, and bodily self-experience that may influence tolerability and perceived benefit. Modifiable environmental factors during infusion may shape these experiences. Music is known to affect mood, attention, and pain perception in non-medical contexts; however, its role during IV ketamine treatment for chronic pain remains poorly understood.

**Objective**: We compared subjective experiences of IV ketamine infusions delivered under usual care versus patient-preferred music conditions.

**Methods**: A randomized controlled trial at the Alan Edwards Pain Management Unit is being conducted. Patients with chronic pain receive IV ketamine as 60-minute infusions (0.5 mg/kg) under usual care and patient-preferred music conditions, separated by five-week washout periods. Semi-structured post-infusion interviews were analyzed using inductive reflexive thematic analysis.

**Results**: Altered embodiment, including sensations of floating, lightness, and detachment from the body, was constructed across accounts as a shared experiential dimension of ketamine treatment. Under usual care, narratives centered on environmental awareness, rumination, and efforts to manage emerging thoughts or anxiety. In contrast, patient-preferred music was described as providing a meaningful frame through which emotions, memories, and reflections were engaged, often emphasizing emotional regulation, focused attention, and positive affect.

**Discussion/Conclusions**: Within this reflexive thematic analysis, auditory context featured prominently in how participants narrated and interpreted their ketamine experiences. From a constructionist perspective, these findings highlight how treatment experiences are co-constituted through pharmacological effects, environmental context, and personal meaning-making, underscoring the relevance of patient-preferred music selection within person-centered ketamine care for chronic pain patients.

## Répondre aux besoins uniques des vétérans en douleur chronique: une revue de la portée des adaptations culturelles militaires dans les programmes d’autogestion numériques

Pascale Marier-Deschênes^1^, Catherine Duclos^1^, Hélène Le Scelleur^2^, Andréa Bergeron^1^

^1^Université Laval, ^2^Chronic Pain Centre of Excellence for Canadian Veterans

**But**: La douleur chronique est plus fréquente chez les vétérans que dans la population civile. Bien que les programmes d’autogestion de la douleur offerts en ligne ou via une application soient de plus en plus accessibles et montrent des effets positifs, leur adaptation aux besoins distincts des vétérans demeure incertaine. Cette revue de la portée visait à documenter la présence et la nature des adaptations culturelles militaires dans ces programmes et à examiner leur influence sur les résultats rapportés par les utilisateurs.

**Méthodologie**: Nous avons inclus des études portant sur les vétérans et les membres des forces armées vivant avec de la douleur chronique et ayant participé à un programme d’autogestion offert sur le web ou via une application. Nous avons examiné comment les adaptations culturelles étaient décrites et si elles influençaient l’expérience utilisateur, l’engagement, la satisfaction, la motivation ou la perception de pertinence culturelle. Une recherche exhaustive a été menée dans les principales bases de données (MEDLINE, CINAHL, Embase, PsycINFO) et la littérature grise pour trouver des études en anglais ou en français publiées depuis 2010.

**Résultats**: Sur les 248 sources examinées, nous avons retenu neuf études provenant des États-Unis et deux du Canada. Dix décrivaient des programmes destinés aux vétérans, mais une seule détaillait le processus d’adaptation. Nous avons identifié divers éléments d’adaptation (p. ex., inclusion de témoignages de vétérans, réduction de la longueur des modules), bien que la plupart aient été décrits de manière superficielle. Aucune étude n’a évalué les effets spécifiques de ces adaptations, à l’exception des perceptions des participants quant à la pertinence culturelle.

**Discussion/Conclusions**: Les descriptions des processus d’adaptation, des composantes adaptées et de leurs effets demeurent limitées. Les recherches futures pourraient impliquer les vétérans dès les premières étapes et évaluer systématiquement la valeur ajoutée des adaptations culturelles militaires.

## Optimizing care through an interdisciplinary hip pain referral triage system: our one-year experience

Casey Wang^1^, Qi Kang Zuo^1^, Riya Virdi^1^, Mataya Lukas^2^, Najam Mian^3^, Parth Lodhia^2^, Mark McConkey^4^

^1^Faculty of Medicine, University of British Columbia, Vancouver, BC, Canada, ^2^Fraser Orthopaedic Research Society, New Westminster, BC, Canada, ^3^Vancouver General Hospital, Vancouver, BC, Canada, ^4^Pacific Orthopedics & Sports Medicine, North Vancouver, BC, Canada

**Introduction**: Hip pain is a prevalent entity to diagnose and treat. Over four years, we established a high-volume integrative clinic for management of hip pain. Comprising an interdisciplinary collaboration of pain specialists, sports physicians and surgeons, our group aims to optimize delivery of surgical and non-surgical care for patients with painful hips. We developed a novel hip pain referral triage system aiming to expedite assessment and management of patients with painful hip conditions. We present our one-year experience after implementing this system.

**Methods**: An interdisciplinary team was assembled in 2020. Monthly interdisciplinary rounds involved sports medicine, pain medicine, orthopaedics, radiology and physiotherapy. Referrals were triaged based on a hub-and-spoke model with clinical information and imaging. Retrospective chart review of triaged referrals from 2023 was performed by three independent reviewers. Data points extracted included demographic information, radiologic diagnosis, initial referral date, treatment pathway (surgical or non-surgical), time to first specialist visit, time to treatment, and procedure. Data is presented through descriptive statistics.

**Results**: 169 patients (208 hips) were referred through our triaging system in the 2023 calendar year. 147 patients were accepted (87% acceptance rate). The most common reason for rejection (11 patients) was due to advanced osteoarthritis requiring arthroplasty. The most common diagnoses were femoroacetabular impingement (83 hips), labral tears (56 hips) and osteoarthritis (49 hips). Mean time from referral to first appointment with a specialist was 104.27 days. Mean time to injection was 156.83 days. Mean time from referral to date of surgery was 271.31 days.

**Discussion/Conclusions**: The development of an interdisciplinary hip pain referral system has streamlined care for patients with non-arthritic hip pain in our province. Future directions include expanding the system’s scope to include pediatric and arthritic hip conditions and establishing a specialized, high-volume hip centre allowing continuing education and research opportunities.

## Low Dose Naltrexone improves pain control and other symptoms in fibromyalgia

Adrienne Junek^1^, Mya Andersen^2^

^1^University of Ottawa, ^2^none

**Introduction**: Fibromyalgia is a chronic pain disorder involving central sensitization, hyperalgesia and other symptoms. Low dose naltrexone (LDN), typically ranging from 1.5 mg to 4.5 mg, is a novel treatment for fibromyalgia and pain disorders involving central sensitization. Its mechanism is proposed to involve hormesis at the opioid receptor, whereas low doses produce a therapeutic effect but higher doses to not.

**Methods**: We performed a chart review of all patients at two integrative health clinics in Ottawa, Ontario who were prescribed LDN for fibromyalgia from 2022-2024. LDN doses were individualized for each patient to optimize therapeutic effects. Data extracted included pain scores from before and after LDN, other health benefits and side effects.

**Results**: LDN produced a marked reduction in pain scores (-3.75, p<0.001) in the 10 patients identified for the chart review. Eight (8) responded to LDN and experienced benefits in pain control, reduction in flares, improved sleep, improved cognitive function, reduced use of other pain medications, and better overall quality of life. Two (2) patients did not benefit from LDN and ceased therapy. Side effects included increased pain, vivid dreams, insomnia and irritability, and resolved with cessation of medication.

**Discussion/Conclusions**: LDN has the potential to produce dramatic reductions in pain for persons with fibromyalgia. Its effects on pain and symptom control may be optimized with individualized dosing and from use in conjunction with comprehensive chronic pain management as provided at an integrative clinic. Its tolerability and potential for pain relief warrant consideration as a therapeutic recommendation for fibromyalgia.

## Immediate effects of virtual reality, gait-like muscle vibration, and transcranial direct current stimulation, alone and combined, on neuropathic pain after spinal cord injury: a pilot study

Pauline Sabalette^1,2^, Cyril Duclos^1^, Diana Zidarov^1,2^, Dorothy Barthélemy^1,2^, Nancy Dubé^2^, Philippe Ménard^2^, Roxane Bouvrette^2^, Élizabeth Arias-Moreno^2^, Mélanie Labelle^2^, Catherine Proulx^3^

^1^School of Rehabilitation, Université de Montréal, Montréal, QC, Canada., ^2^Institut Universitaire sur la Réadaptation en Déficience Physique de Montréal — Centre for Interdisciplinary Research in Rehabilitation, Montréal, QC, Canada, ^3^National Research Council Canada, Ottawa, ON, Canada

**Introduction**: Neuropathic pain is a frequent, disabling consequence of spinal cord injury (SCI), and remains challenging to manage due to unclear mechanisms and treatment-related side effects. Innovative approaches such as virtual reality (VR), muscle vibration (MV), and transcranial direct current stimulation (tDCS) have shown promising results in pain perception. We aim to evaluate, with a quasi-experimental pilot study, the immediate effect of VR, gait-like MV and tDCS combined or alone on neuropathic pain in individuals with SCI.

**Methods**: Four adults with neuropathic pain participated in four weekly sessions. Each session began with a single-blind application of active or sham tDCS for twenty minutes, delivered in a pseudo-randomized order across sessions. Participants then received three ten-minute interventions, also in pseudo-randomized order: MV alone, VR alone using a walking self-avatar, and a combined VR+MV condition designed to enhance the illusion of gait-related movement. Pain intensity was measured immediately before and after each stimulation using a numeric rating scale ranging from zero to ten, with a minimal clinically important difference defined as a reduction of two points or more.

**Results**: The results indicated that participants reported significant reduction of pain in 4/7 stimulations where VR was associated with muscle vibration, in 1/8 for VR-alone stimulations and in 1/7 for MV-only stimulations. Significant change in pain was found in 1/8 sham tDCS, but not after active tDCS.

**Discussion/Conclusions**: Combining VR with gait-like muscle vibration may produce immediate pain relief, warranting further investigation. No change in pain was observed after tDCS, likely due to its once-weekly application.

## Exploring intimacy and chronic pain: An embedded evaluation of the Alberta Virtual Pain Program’s virtual group for women

Elena Lopatina^1,2^, Tina Hoang^1,2^, Magali Robert^1,2^, Lindsey Kaupp^2^

^1^University of Calgary, ^2^Primary Care Alberta

**Introduction**: Intimacy is often affected by chronic pain, yet practical, evidence-based support is rare in publicly funded care. We aimed to describe women’s experiences with the Alberta Virtual Pain Program’s group on intimacy and chronic pain, assess its fit with patient needs, and identify priorities for improvement.

**Methods**: The group was delivered as a single 90-minute Zoom workshop and facilitated by two non-physician clinicians (physiotherapist/occupational therapist). The session combined didactic content on intimacy, relationships, emotional connection, communication with Q&A and discussion. All attendees were invited to complete an anonymous post-session survey; categorical data were summarized descriptively and open-ended responses analyzed thematically. Attendance and participant demographics were obtained from the electronic medical record.

**Results**: Between July and November 2025, the group ran twice; 12 patients registered and 6 attended (mean (SD) age of 46 (12) years). Among survey respondents (N=5), 100% agreed the content was relevant and 80% agreed the format and delivery were appropriate. Four themes emerged: (1) positive experience with evidence-based, practical content and a frank, safe space (including relevance beyond romantic relationships); (2) recommendations for more time (multi-session series), Q&A, and peer support; (3) ongoing needs for skills, resource navigation, and peer-based support; and (4) recognition of the centrality of intimacy challenges and limited non-equitable access to appropriate supports in the publicly funded system.

**Discussion/Conclusions**: A one-session virtual group on intimacy and chronic pain was feasible, acceptable, and perceived as highly relevant, but participants identified a clear need for more intensive and ongoing support.

## L’effet de la neurostimulation périphérique sur la douleur induite par l’exercice chez les femmes vivant avec la fibromyalgia

Mathil Ruel^1,2^, Shayana Lussier Crevier^1^, Nicolas Dehors^3^, Serge Marchand^3^, Isabelle J. Dionne^1,2^, Guillaume Léonard^2,3^

^1^Faculté des sciences de l’activité physique, Université de Sherbrooke, Sherbrooke, Qc, Canada., ^2^Centre de recherche sur le vieillissement, Sherbrooke, CIUSSS de l’Estrie -CHUS, Université de Sherbrooke, Sherbrooke, Canada., ^3^Faculté de médecine et des sciences de la santé, Université de Sherbrooke, Sherbrooke, QC, Canada.

**Aim**: La fibromyalgie, caractérisée par des douleurs persistantes et diffuses, impacte significativement la qualité de vie. L’entraînement contre résistance présente des bénéfices chez les personnes atteintes de fibromyalgie, mais les douleurs exacerbées par le mouvement dans les premières semaines d’un programme d’exercices représentent une barrière majeure à l’adhésion. Cette étude visait à évaluer l’effet à court terme d’une séance de neurostimulation périphérique (TENS), appliquée pendant un exercice contre résistance des membres inférieurs (*leg press)*, sur l’intensité et l’aspect désagréable de la douleur chez les femmes vivant avec la fibromyalgie.

**Méthodologie**: Cette étude randomisée, à double insu, a été menée chez 21 femmes atteintes de fibromyalgie. Les participantes ont réalisé 2 séries de 10 répétitions à l’exercice du *leg press* (60% du 1-RM estimé) combiné à une TENS active conventionnelle (100 Hz, 60 µs; n=10) ou une TENS placebo (stimulation simulée; n=11). La douleur (intensité et aspect désagréable) a été évaluée avec une échelle numérique de 0 à 10 avant l’exercice, immédiatement après la 2^e^ série, 15-min après, en fin de journée, ainsi que à 24h après l’exercice.

**Résultats**: Comparée au placebo, la TENS réelle a réduit l’intensité et l’aspect désagréable de la douleur de l’ensemble du corps immédiatement après l’exercice, en plus de réduire son intensité 15-min après l’exercice (tous les p < 0,01). À 15-min après l’exercice, 90% des participantes de la condition TENS active ont atteint une réduction ≥15 % de l’intensité de la douleur contre aucune pour la condition placebo.

**Discussion/Conclusion**: Ces résultats suggèrent qu’une application de TENS conventionnelle peut réduire de façon cliniquement significative la douleur aiguë induite par une séance d’exercice contre résistance chez des femmes atteintes de fibromyalgie. L’utilisation de la TENS pendant l’exercice pourrait faciliter l’adhésion à un programme, bien que ses effets à long terme restent à être évaluer.

## Applying an Intersectionality Framework to Understand Inequities in Chronic Pain Care

Karime Mescouto^1^, Jenny Setchell^2,3^

^1^Western University, ^2^The University of Queensland, ^3^Institute for Urban Indigenous Health

**Introduction**: Marginalized groups experience greater pain, worse outcomes, and unequal treatment within pain care. However, the *intersecting* effects of multiple systems of power on these inequities remain underexplored. Intersectionality offers a valuable framework to examine how social categories (e.g., race, class, gender, ethnicity, age) interact with structural forces to shape experiences of oppression. This qualitative study explored how applying an intersectionality framework provides insight into the experiences of people with chronic pain and their interactions with clinicians.

**Methods**: Ten ethnographic observations of clinical encounters, selected from a broader dataset of 39 at a multidisciplinary pain clinic were critically analyzed. The concept of intersectionality as a theoretical lens was used to provide insights into how social categories operate as reciprocally constructing phenomena shaping complex social inequities in pain management.

**Results**: Findings revealed that multiple intersecting identities influence pain care. Being labelled a “pain patient” functioned as a social category that, when intersecting with others—such as being visibly disabled, non-white, or from a different linguistic background—intensified marginalization and stigmatization during clinical encounters. These intersecting disadvantages operated across individual, interpersonal, and systemic levels, shaping both patient experiences and clinician responses.

**Discussion/Conclusions**: Applying an intersectionality lens illuminates how overlapping social categories and power relations reinforce inequities in pain management. Recognizing these dynamics can support clinicians, researchers, and policymakers in developing more equitable, context-sensitive approaches to pain care. Integrating intersectionality into clinical practice and research provides a pathway toward understanding and addressing the complex social realities of people living with pain.

## “A Tale of Two Rhizotomies: Comparing Lumbar Medial Branch vs. Pseudojoint Capsule Ablation in Bertolotti Syndrome”

Virginia McEwen^1,2^, Yuvaraj Kotteeswaran^1,2^

^1^Thunder Bay Regional Health Sciences Centre, ^2^NICHE Pain Care

**Introduction**: Bertolotti syndrome, a congenital lumbosacral transitional vertebra (LSTV) creating an articulation between the L5 transverse process and the sacrum or ilium, is an underrecognized source of low back pain. Conventional radiofrequency ablation (RFA) targets the medial branches innervating lumbar facet joints, yet these nerves may not capture nociceptive input from the anomalous pseudojoint. Consequently, interventional guidance for rhizotomy in this population is lacking. This case-based comparison examines whether direct RFA of the pseudojoint capsule provides superior pain relief to standard medial branch neurotomy in symptomatic Bertolotti syndrome.

**Methods**: Two adults with chronic axial low back pain and imaging-confirmed Bertolotti syndrome (Castellvi type IIa) underwent sequential RFA. Each first received conventional thermal RFA of the L4-L5 and L5-S1 medial branches. Following limited benefit, both underwent image-guided RFA targeting the pseudojoint capsule between the enlarged L5 transverse process and sacral ala after diagnostic block confirmation. Pain intensity and function were assessed at 8 weeks and 2 years post-procedure.

**Results**: Medial branch RFA yielded only partial improvement. Pseudojoint-targeted RFA produced substantially greater and more durable reductions in pain and improved function, including increased standing tolerance and reduced analgesic use. No complications occurred.

**Discussion/Conclusions**: Pain in Bertolotti syndrome appears to originate primarily from the pseudojoint capsule rather than facet-mediated pathways. Direct pseudojoint ablation offered superior and sustained relief. Recognizing this structure as a distinct nociceptive source may refine interventional strategies and warrants further prospective study to optimize technique and outcomes.

## Surgical decompression with or without fusion for degenerative lumbar spondylolisthesis: A systematic review and meta-analysis

Qi Zhou^1^, Jason Busse^2^, Arnav Agarwal^3^, Chunjuan Zhai^4^, Mingdong Yang^1^, Sohail Bajammal^5^, Iliya Khakban^2^, Amy Jing^2^, Layla Bakaa^2^, Saranya Srikanthan^2^, Rachel Couban^2^, Markian Pahuta^2^, Raja Rampersaud^6^, Zhikang Ye^2^

^1^Lanzhou University, ^2^McMaster University, ^3^University of Alberta, ^4^Shandong First Medical University, ^5^Fakeeh Hospital, Saudi Arabia, ^6^University of Toronto

**Introduction**: Although decompression with fusion remains widely used for patients with degenerative lumbar spondylolisthesis, recent evidence suggests comparable outcomes with decompression alone. We conducted a systematic review to inform this issue.

**Methods**: We systematically searched MEDLINE, Embase, the Cochrane Central Register of Controlled Trials (CENTRAL), and Web of Science from inception, with no language restrictions. Eligible randomized controlled trials (RCTs) compared surgical decompression plus lumbar fusion versus decompression alone in patients with degenerative lumbar spondylolisthesis. We converted all measures of pain intensity to a 10‑cm visual analogue scale; and all measures of physical functioning to the 100‑point Oswestry Disability Index (ODI). Meta-analyses were conducted using random-effects models, and results were expressed as mean differences (MD) with 95% confidence intervals (CIs). We assessed the certainty of evidence with the GRADE approach.

**Results**: Eleven publications involving five RCTs with 560 patients were included for review. Compared with decompression alone, decompression plus fusion probably results in little to no difference in back pain at 2 years (MD 0.68, 95% CI 0.30 to 1.07; moderate certainty) or at 5 years (MD 0.34, 95% CI -0.21 to 0.89; moderate certainty). Similarly, no important difference was observed in leg pain at 2 years (MD 0.28, 95% CI -0.05 to 0.62; high certainty) or at 5 years (MD 0.12, 95% CI -0.56 to 0.79; moderate certainty). Physical function scores showed little to no difference at 2 years (MD -0.05, 95% CI -2.63 to 2.54; high certainty;) or at 5 years (MD -1.69, 95% CI -4.23 to 0.85; high certainty). However, decompression plus fusion was associated with longer hospital stay (MD 1.70 days, 95% CI 1.65 to 1.75; high certainty;).

**Discussion/Conclusions**: Moderate to high certainty evidence found decompression plus fusion does not improve back pain, leg pain, or physical function compared with decompression alone for degenerative lumbar spondylolisthesis.

## Spinal decompression alone or with instrumented fusion for adults with degenerative lumbar spondylolisthesis: A clinical practice guideline

Arnav Agarwal^1^, Sohail Bajammal^2^, Jason Busse^3^, Zhikang Ye^3^, Per Vandvik^4^, Thomas Agoritsas^5^, Gordon Guyatt^3^, Øystein Nygaard^6^, Wei Bu^7^, Carlos Tucci^8^, Ian Vlok^9^, Koji Tamai^10^, Rachel Couban^3^, Ian Harris^11^, Manuela Ferreira^12^, Hilde Verbeke^13^, Liza Kirtchuk^14^, Rikke Jensen^15^, Janet Gunderson^16^, Gary Foster^17^, Andrew Thomas^18^

^1^University of Alberta, ^2^Fakeeh Hospital, Saudi Arabia, ^3^McMaster University, ^4^University of Oslo, Norway, ^5^University of Geneva, Switzerland, ^6^Norwegian University of Science and Technology, ^7^Hebei Medical University, ^8^Hospital Israelita Albert Einstein, ^9^University of Stellenbosch and Tygerberg Academic Hospital, ^10^Osaka Metropolitan University, ^11^Ingham Institute for Applied Medical Research, Liverpool, ^12^The George Institute for Global Health, ^13^University Hospitals Leuven, ^14^King’s College London School of Medicine, ^15^University of Southern Denmark, ^16^Patient/Caregiver Partner, ^17^The Canadian Veterans Chronic Pain Centre of Excellence, ^18^Canadian Armed Forces Health Services Centre

**Introduction**: Degenerative lumbar spondylolisthesis commonly leads to surgery when symptoms persist despite conservative care. Although decompression is the standard procedure, instrumented fusion is frequently added despite uncertain benefit, higher costs, and wide international variation in practice.

**Methods**: An international panel including patients, clinicians, and methodologists produced a recommendation following standards for trustworthy guidelines and using the GRADE approach. The panel considered evidence regarding benefits and harms and associated certainty of evidence for spinal decompression with and without the addition of instrumented fusion, values and preferences of typical patients living with degenerative lumbar spondylolisthesis, resource considerations, feasibility, acceptability and equity considerations.

**Results**: A systematic review and meta-analysis of randomized controlled trials found the addition of instrumented fusion results in little or no difference in back and leg pain and physical function (moderate to high certainty), longer index hospital length of stay (high certainty) and worsened emotional role function (moderate certainty) at both 2- and 5-year follow-up. Fusion probably worsens physical role function and social function at two years follow-up, though effects on both outcomes are attenuated at five-year follow-up (all moderate certainty). A systematic review and meta-analysis of cohort studies found the addition of fusion probably results in an increased risk of non-union, pseudo- arthrosis and adjacent stenosis (moderate certainty).

**Discussion/Conclusions**: The guideline panel considered evidence regarding benefits and harms as well as resource considerations (cost and cost- effectiveness) and ultimately issued a **strong recommendation against** spinal decompression with instrumented fusion, compared to spinal decompression alone, for adults living with symptomatic low-grade degenerative lumbar spondylolisthesis.

## The Alberta Virtual Pain Program - a province-wide virtual chronic pain program in primary and community care settings: Uptake, satisfaction, and early outcomes.

Elena Lopatina^1,2^, Tina Hoang^1,2^, Magali Robert^1,2^

^1^University of Calgary, ^2^Primary Care Alberta

**Introduction**: The Alberta Virtual Pain Program (AVPP) is Canada’s first publicly funded, province-wide chronic pain program. It was developed to address gaps in care by integrating evidence-based, non-pharmacologic, multidisciplinary team supports within primary and community care settings.

**Methods**: March 2024-August 2025 intake and attendance data were extracted from electronic medical records. Patient-reported outcome measures (PROMs)—the Brief Pain Inventory-Short Form (BPI-SF) and the Pain Self-Efficacy Questionnaire (PSEQ)—were collected via survey at baseline and end-of-program; end-of-program and 12-month surveys also assessed patient satisfaction and perceived impact on well-being. Referrals and uptake were summarized as counts; attendance as mean (SD) across cohorts for clinician- and peer support worker (PSW)-led sessions, and categorical survey items as percentages. Pre-post changes in PROMs were tested with paired t-tests.

**Results**: A total of 823 referrals; 311 individuals attended group programming. Mean (SD) attendance was 72%(22%) and 50%(24%) for clinician- and PSW-led sessions, respectively. Pre-post analyses showed clinically and statistically significant improvements in PROMs: BPI-SF pain interference −0.84 (95% CI:−1.38 to −0.31;p=0.003) and PSEQ +3.63 (95% CI:+2.20 to +5.07;p<0.0001). At program end, 93% and 96% were satisfied with clinician- and PSW-led sessions, respectively. At 12-months, 92% reported the program helped them feel better.

**Discussion/Conclusions**: AVPP received the 2025 Health Quality Alberta Patient Experience Award for patient-centred care. It is a feasible, effective model for virtual multidisciplinary chronic pain care in primary and community settings. High attendance, clinical improvements, and strong patient satisfaction underscore the need for sustained funding to maintain and scale the impact.

## Multi-Family DBT-A for Adolescents with Chronic Pain

Marijana Jovanovic^1^, Stefan Domaradzki^2^, Michelle Nieuwesteeg^2^, Heidi Eccles^2^

^1^CHEO, uOttawa, ^2^CHEO

**Introduction**: Chronic pain persisting for 3 months or longer is experienced by 11-38% of youth leading to sleep disruption, reduced physical activity, social difficulties, school absenteeism and mental health concerns. Maladaptive emotional regulation is a risk factor, and interventions such as Dialectical Behavior Therapy (DBT) targeting emotional dysregulation and associated impacts may reduce pain and improve quality of life. We examined the effectiveness of the multi-family DBT-A group offered by CHEO Chronic Pain Services.

**Methods**: We prospectively collected pre- and post-intervention measures from youth and their caregivers (N=22) in CHEO’s multi-family DBT-A program to assess functional impairment (Bath Adolescent Pain-Parent Impact Questionnaire [BAP-PIQ]), pain acceptance (Parent Pain Acceptance Measure [PPAQ] or Chronic Pain Acceptance Questionnaire-Adolescent version [CPAQ-A]), pain catastrophizing (Pain Catastrophizing Scale -Child or Parent [PCS-C/P]), emotional regulation (Difficulties in Emotional Regulation Scale-Short Form [DERS-SF]), and quality of life (Pediatric Quality of Life Inventory [PedsQL]). A post-intervention satisfaction survey was distributed to all participants.

**Results**: BAP-PIQ scores decreased post-intervention indicating caregivers perceived improvement in their child’s impairment following DBT-A. Mean PPAQ scores increased from 25 to 30, while PCS-P decreased from 26 to 22, indicating caregivers developed greater acceptance and reduced negative cognitive emotional responses to their child’s pain. The PedsQL showed increased physical functioning following DBT-A, and CPAQ-A scores indicated greater activity engagement and pain willingness. PCS-C scores decreased from 34 to 17.5 and DERS decreased from 58 to 54, indicating decreased pain catastrophizing and improved emotional regulation. Overall, 100% of caregivers and 80% of youth were satisfied with the quality of the DBT-A program.

**Discussion/Conclusions**: This study suggests that multi-family DBT-A may have a positive impact on youth with chronic pain and their caregivers, potentially resulting in decreased pain catastrophizing, and improved emotional regulation and physical functioning.

## Effectiveness and acceptability of remotely delivered psychotherapies for management of chronic non-cancer pain

Shiva Shahabi^1^, Azin Khosravirad^1^, Nora Razoki^2^, João Pedro Lima^1^, Andrea J Darzi^2^, Lawrence Mbuagbaw^1^, Jason W. Busse^2^, Behnam Sadeghirad^2^

^1^Department of Health Research Methods, Evidence, and Impact (HEI), McMaster University, Hamilton, Ontario, Canada, ^2^Department of Anesthesia, McMaster University, Hamilton, Ontario, Canada

**Introduction**: Chronic non-cancer pain (CNCP) is a widespread and debilitating condition affecting millions globally. Our study assesses the effectiveness and acceptability of remotely delivered psychotherapies for CNCP management through a systematic review and network meta-analysis (NMA).

**Methods**: We searched multiple electronic databases for randomized controlled trials that compared remotely delivered psychotherapies (and their combinations with other active treatments) with usual care and active interventions. We conducted a frequentist random-effects NMA to compare interventions across patient-important outcomes. All analyses were conducted at three time points: evaluation after treatment ended, 6-month after the end of treatment, and longest follow-up between 6 to 12 months after treatment ended.

**Results**: A total of 66 trials (8,993 participants) were included. At post-treatment, compared to usual care, remote ACT (r-ACT) and remote CBT (r-CBT) probably result in slight reductions in pain intensity (MD: -0.59, 95% CI: -0.81 to -0.37, and MD: -0.36, 95% CI: -0.50 to -0.23), and slight improvements in quality of life (MD: 0.06, 95% CI: 0.02 to 0.10, and MD: 0.05, 95% CI: 0.03 to 0.08, respectively). r-ACT likely results in a slight improvement in physical function (MD: 5.76, 95% CI: 2.67 to 8.86), and r-CBT may result in a slight improvement in physical function (MD: 3.10, 95% CI: 1.22 to 4.97) compared to usual care. r-ACT and r-CBT likely result in slight improvements in depression and anxiety. For sleep quality, r-ACT probably results in a slight reduction in insomnia (MD: -2.51, 95% CI: -4 to -1.02). The dropout rates likely increased with r-CBT (RR: 1.55, 95% CI: 1.18 to 2.03) and r-ACT (RR: 1.46, 95% CI: 1.18 to 1.81) compared to usual care.

**Discussion/Conclusions**: Remotely delivered psychotherapies, particularly r-ACT and r-CBT, offer slight short-term benefits for chronic pain management with limited durability of benefits at long-term and higher dropout rates.

## User-Centred, Iterative Usability Testing of a Technology-Enabled Joint Protection Program for Hand Osteoarthritis

Dimitra V Pouliopoulou^1^, Victoria D’Alessandro^1^, Nicole Billias^1^, Joy C MacDermid^1^, Yuxin (Monica) Lin^1^, Emily Lalone^1^, Ruby Grewal^1^, Pavlos Bobos^1^

^1^Western University

**Introduction**: Hand osteoarthritis is a leading cause of pain, disability, and reduced quality of life in older adults. Joint protection programs are recommended as a core component of self-management, but traditional delivery is limited by barriers to access. Digital programs can overcome these challenges for some people, but their reach and effectiveness depends on usability.

**Methods**: We conducted a mixed methods usability study of a remotely delivered joint protection program designed for people with hand osteoarthritis. Twenty-three participants took part, recruited through purposeful sampling to ensure inclusion of groups often underrepresented in research. Usability was assessed using predefined task completion, browser-based eye-tracking, participant ratings, and think-aloud protocols, with iterative refinements applied between participants.

**Results**: Routine navigation tasks, such as navigating between different modules, accessing interactive activities, and viewing short videos, were consistently completed with high success. More complex interactive tasks, including drag-and-drop activities, scenario-based modules, and toggling videos to full screen, initially posed challenges. Over successive iterations, however, usability improved markedly, with later participants achieving near-perfect performance. Qualitative analysis revealed that participants valued clear language, short and focused videos, interactive elements, and the ability to proceed at their own pace, while raising concerns about excessive clicking, unclear instructions, and variation in age representation. Iterative refinements, including platform adjustments, clearer instructions and an introductory video, addressed these issues and contributed to improved performance.

**Discussion/Conclusions**: This study demonstrates that a remotely delivered, technology-enabled joint protection program for hand osteoarthritis is usable, accessible, and engaging across a diverse sample. Beyond refining the program itself, the study introduces a practical framework for iterative, equity-informed usability testing that can inform the design of future digital health interventions.

## Cartographie des programmes d’éducation et d’autogestion offerts dans les cliniques de douleur et les centres de réadaptation au Québec.

Orlane Ballot^1,2^, Pascale Marier-Deschênes^1,2^, Yannick Tousignant-Laflamme^3^, Mark Ware^4^, Christina Gentile^5^, Philippe De Grandpré^6^, Anne Marie Pinard^1,2^

^1^CIRRIS, ^2^Université Laval, ^3^Université de Sherbrooke, ^4^Université McGill, ^5^Hopital général de Montréal, ^6^Société québécoise de la douleur

**Introduction**: Au Québec, l’accès aux services spécialisés en douleur chronique demeure limité. Pour favoriser l’autonomie des patient(e)s, plusieurs cliniques ont mis en place des programmes d’éducation et d’autogestion. Cette étude vise à cartographier ces initiatives et à identifier les obstacles et les facilitateurs à leur mise en œuvre.

**Méthodologie**: Parmi 33 cliniques de douleur (CD) et centres de réadaptation (CR), 27 offrent un programme actif. Des entrevues ont été réalisées avec les responsables afin d’explorer 5 dimensions: développement, accès, format et contenu, facilitateurs et obstacles, ainsi que l’évaluation des effets.

**Résultats**: Les programmes ciblent principalement les adultes de 18 à 80 ans, avec quelques initiatives pédiatriques et gériatriques. Offerts majoritairement en groupe et en présentiel, certains sont également accessibles en virtuel. Leur durée varie de séances uniques d’une demi-journée à des séries de dix séances ou plus, d’une durée de deux à trois heures chacune. Le contenu est relativement homogène: bases neurophysiologiques de la douleur, dimensions psychosociales, activité physique, approches thérapeutiques, hygiène de vie et aspects communautaires. Certains intègrent des exercices pratiques, proposent des capsules vidéo et distribuent du matériel éducatif. Les facilitateurs incluent la cohésion d’équipe, l’interdisciplinarité, le soutien des gestionnaires et médecins, les interactions humaines et la motivation des patient(e)s. Les obstacles récurrents sont le manque de locaux, de matériel, de financement, l’absence de corridors de soins, le roulement du personnel, l’absentéisme et certaines résistances organisationnelles. Plusieurs programmes disposent de mécanismes de rétroaction, mais les ressources pour analyser les données sont limitées. La présence d’un proche est généralement autorisée. Ces éléments varient selon les milieux, les ressources et les équipes.

**Discussion/Conclusions**: Bien structurés et appréciés, ces programmes sont confrontés à des contraintes logistiques et à un manque de ressources. Un soutien accru est nécessaire pour assurer leur pérennité et maximiser leurs retombées cliniques, éducatives et systémiques.

## Patients’ Perspectives on Pain Management by Physiotherapists in Primary Care and on Emerging Advanced Practice roles in the Province of Quebec

Maxime Charron^1^, Sonia Zouaoui^1^, Marc-Antoine Bouffard^2^, Cristina Leblanc^3^, Jean-Sébastien Roy^4, 5^, Véronique Lowry^1,2^, Kadjia Perreault^4^, Marie-ève Poitras^6^, François Desmeules^1,2^

^1^Hôpital Maisonneuve-Rosemont Research Center, Université de Montréal Affiliated Research Center, Montreal, Quebec, Canada, ^2^School of Rehabilitation, Faculty of Medicine, Université de Montréal, Montreal, Quebec, Canada, ^3^Centre intégré de santé et de services sociaux de la Montérégie-Est (CISSSME), Quebec, Canada, ^4^School of Rehabilitation Sciences, Faculty of Medicine, Université Laval, Quebec City, Quebec, Canada, ^5^Centre for Interdisciplinary Research in Rehabilitation and Social Integration, Quebec City, Quebec, Canada, ^6^Department of Family Medicine, Université de Sherbrooke, Sherbrooke, Quebec, Canada

**Introduction**: Musculoskeletal disorders (MSKDs) are a leading cause of persistent pain and disability. Integrating physiotherapists (PTs) as first-contact practitioners in Family Medicine Groups (FMGs) and in advanced practice roles has shown promise for improving access, enhancing pain management, and increasing care efficiency. Understanding patients’ perspectives on these emerging models is essential.

**Purpose**: To explore patients’ experiences and perceptions of pain management on receiving physiotherapy care in FMGs including in first-contact advanced practice roles.

**Methods**: A qualitative study was conducted using individual semi-structured interviews with patients who received physiotherapy care in three FMGs. An inductive thematic analysis identified patients’ pain-related expectations, perceptions, and satisfaction levels.

**Results**: Twelve patients with various pain-related MSKDs (8 women, 4 men, mean age: 66 ± 13 years) were interviewed. Five themes emerged: 1) Participants expected pain relief, patient-centred care, advice and education, and home exercises from PTs; 2) They experienced comprehensive, attentive care and felt well supported and understood by treating PTs; 3) Participants perceived PTs as competent experts, trustworthy, and their role as necessary in FMGs; 4) Participants valued easy access to efficient multidisciplinary care from PTs and collaborating health care practitioners; 5) Participants supported the expanding advanced practice roles for PTs.

**Discussion/Conclusions**: Patients valued PTs’ role in addressing pain management comprehensively through education, exercises, and collaborative care, and expressed strong support for the integration of PTs in FMGs.

## Changes in pain catastrophizing and related outcomes following a single-session Empowered Relief intervention delivered by physical therapists in workers with low back pain: A pilot study

Junie Carriere^1^, Marie-France Coutu^1^, Beth Darnall^2^, Guillaume Léonard^1^, Marie-Pier Royer^1^, Mei Yue Li^1^, Martine Bordeleau^1^, Marie-José Durand^1^

^1^université de sherbrooke, ^2^Stanford University

**Aim**: Empowered Relief is a 2-hour single-session pain management skills intervention that has demonstrated efficacy at improving pain outcomes in individuals with chronic low back pain. The objectives were 1) to assess the acceptability of Empowered Relief delivered by physical therapists in a sample of French-Canadian workers with low back pain; and 2) to explore changes in pain catastrophizing and other pain-related outcomes following Empowered Relief and physical therapy.

**Methods**: The study was an uncontrolled prospective pilot trial of Empowered Relief and physical therapy for low back pain. Participants were 63 French-Canadian individuals undergoing physical therapy for subacute and chronic low back pain (<1 year duration). Participants completed baseline measures of demographic and measure of pain catastrophizing, pain intensity, symptoms of anxiety and depression, physical function and pain interference before taking part in a single-session 2-hour Empowered Relief virtual course. Participants then completed a post-class acceptability questionnaire and a 4-week follow-up questionnaire assessing the same measures as baseline.

**Results**: A 2-hour single-session of Empowered Relief demonstrated high acceptability among participants. Pain catastrophizing scores reduced by an average of 28 points, with 90% achieving clinically significant change. Participation in Empowered Relief and physical therapy was associated with reductions in pain intensity, and clinically meaningful improvements in symptoms of anxiety and depression, physical function and pain interference at 4-weeks follow-up. Effect sizes were moderate to large, and the largest changes were found in individuals with subacute low back pain.

**Discussion/Conclusions**: Empowered Relief, when delivered by physical therapists, may offer a rapid, scalable intervention to complement physical therapy and improve early outcomes in workers with low back pain. The results provide evidence to support a future randomized controlled trial evaluating the effect of Empowered Relief delivered by physical therapists for low back pain.

## Primary Care Nurses’ Involvement in Chronic Pain Management: A Focus Group Study of Barriers and Facilitators

Andréanne Bernier^1^, Marie-Eve Poitras^2^, Marie-Dominique Poirier^2^, Sylvie Beaudoin^1^, Anaïs Lacasse^1^

^1^Université du Québec en Abitibi-Témiscamingue, ^2^Université de Sherbrooke

**Aim**: Chronic pain (CP) affects one in five Canadians and incurs nearly CAD 40 billion in costs, with patients often facing limited access to primary care. While primary care nurses are central to chronic disease management, their involvement in CP care remains limited. Understanding the factors that enable effective implementation of CP management in primary care is essential to optimize patient care. This qualitative study explored barriers and facilitators influencing the integration of nursing activities in CP management in primary care.

**Methods**: Twenty-one primary care nurses in Quebec, Canada, participated in four online focus groups. The i-PARIHS framework informed the design of our focus group discussion guide and the thematic analysis of factors shaping nurses’ involvement in CP care.

**Results**: Overlapping with chronic disease care, nurses expressed interest in pain assessment and multimodal management but faced challenges due to limited training, confusion with acute pain, and lack of practical tools. Despite motivation, difficulties in providing adequate follow-up and addressing mental health were further compounded by unclear role boundaries, time constraints, and fact that medication prescribing falls under physicians’ scope of practice. Leadership from primary care managers, peer support, and coordination with specialized services emerged as critical facilitators for implementing change and promoting interdisciplinary collaboration.

**Discussion/Conclusions**: The study highlights professional and organizational barriers to integrating nursing activities for CP. Targeted training, tailored tools, clarification of nursing roles, inter-organizational mentorship, and inclusion of nurses in care planning are essential strategies to strengthen their capacity and enhance CP management in primary care settings

## The impact of chiropractic care on prescription opioid use for non-cancer spine pain: a systematic review and meta-analysis

Peter Emary^1^, Kelsey Corcoran^2^, Brian Coleman^3^, Amy Brown^4^, Carla Ciraco^5^, Jenna DiDonato^6^, Li Wang^1^, Rachel Couban^1^, Abhimanyu Sud^7^, Jason Busse^1^

^1^McMaster University, ^2^Brown University School of Public Health, ^3^Yale School of Medicine, ^4^Private Practice, Cambridge, Ontario, ^5^Private Practice, Vaughan, Ontario, ^6^Private Practice, Ancaster, Ontario, ^7^University of Toronto

**Introduction**: Opioids are commonly prescribed for spine-related pain; however, emerging evidence suggests that access to chiropractic care may reduce reliance on opioids. We conducted a systematic review and meta-analysis to assess the impact of chiropractic care on new or continued prescription opioid use among adults with non-cancer spine pain.

**Methods**: We searched for eligible randomized controlled trials (RCTs) and observational studies indexed in MEDLINE, Embase, AMED, CINAHL, Web of Science, and the Index to Chiropractic Literature to March 20, 2025. Paired reviewers independently assessed risk-of-bias and extracted data. We performed random- and fixed-effects meta-analyses and used GRADE to assess the certainty of evidence.

**Results**: In total, two RCTs (838 participants) and 18 cohort studies (6,035,220 participants) were included in our analyses. We found very low certainty evidence that, compared to standard medical care alone, receipt of chiropractic care may reduce the odds of receiving prescription opioids by 64% (OR = 0.36; 95% CI, 0.25 to 0.52; absolute risk reduction [ARR] 15%). However, we found evidence of a credible subgroup effect based on timing of receipt of chiropractic care. Specifically, we found very low certainty evidence that receiving chiropractic services within the first 30-days of presenting with spine-related pain may decrease the odds of receiving opioids by 67% (OR = 0.33; 95% CI, 0.22 to 0.51; ARR 15%) and by 27% if chiropractic care is received later than 30-days after presentation (OR = 0.73; 95% CI 0.53 to 0.99; ARR 8%; test of interaction, p<0.001).

**Discussion/Conclusions**: Our systematic review found very low certainty evidence that receipt of chiropractic care may be associated with lower odds of receiving prescription opioids or initiating long-term opioid use among adults with non-cancer spine pain, particularly when chiropractic care is provided earlier versus later. Rigorously designed RCTs are needed to confirm these results.

## ANATOMY OF FAILURE: Understanding Clinical, Psychological, and Social Correlates of non-responders to an Interdisciplinary Pain Management program

Angela Mailis^1^, Karen Spivak^1^, Shehnaz Fatima Lakha^1^

^1^Pain & Wellness Centre Vaughan ON

**Introduction**: Pain programs integrating medical, psychological, and physical therapies improve outcomes for many chronic pain patients; however, a subset demonstrates minimal/no improvement. This study explores the demographic, psychological, and functional characteristics of non-responders to an interdisciplinary pain management program, to identify factors associated with failure to improve.

**Methods**: A retrospective cross-sectional analysis obtained data from 352 patients with chronic non-cancer pain who completed a community-based 3-month interdisciplinary pain management program (2019-2025). Non-responders scored minimal or no improvement on the Global Impression of Change scale. Extracted data included demographics, pain characteristics, emotional and functional status (BPI, CES-D, GAD-7, PCS, PSEQ, CPAQ, TSK).

**Results**: Of the 352 patients 23% were non-responders, with 32% of males versus 20% of females having failed the program. Male non-responders were younger (40.2 vs 46.6 years old); more likely to be employed (68 vs 43%); with less BPI pain interference and severity, depression and anxiety scores, and higher self-efficacy. However, they presented with less pain acceptance and higher catastrophizing and kinesiophobia.

**Discussion/Conclusions**: The prevalence of male non-responders to an interdisciplinary pain management program is noteworthy. Preliminary analysis shows that women present with higher pain ratings and greater impact on functioning, but experience much higher rates of improvement, as opposed to men who tended to show more maladaptive cognitive patterns and displayed higher failure rates. These findings suggest that sex-specific treatment approaches may be beneficial - addressing depression and functional restoration in women, and cognitive restructuring around pain beliefs and acceptance of limitations in men.

